# Organic linkers control the thermosensitivity of the emission intensities from Tb(iii) and Eu(iii) in a chameleon polymer†
†Electronic supplementary information (ESI) available: Computational and experimental details, and the Cartesian coordinates. See DOI: 10.1039/c6sc03006h



**DOI:** 10.1039/c6sc03006h

**Published:** 2016-08-25

**Authors:** Miho Hatanaka, Yuichi Hirai, Yuichi Kitagawa, Takayuki Nakanishi, Yasuchika Hasegawa, Keiji Morokuma

**Affiliations:** a Department of Chemistry , Faculty of Science and Engineering , Kindai University , Higashi-Osaka , Osaka 577-8502 , Japan . Email: hatanaka@chem.kindai.ac.jp; b PRESTO , Japan Science and Technology Agency (JST) , 4-1-8 Honcho , Kawaguchi , Saitama 332-0012 , Japan; c Faculty of Engineering , Hokkaido University , Sapporo , Hokkaido 060-8628 , Japan . Email: hasegaway@eng.hokudai.ac.jp; d Fukui Institute for Fundamental Chemistry , Kyoto University , Kyoto 606-8103 , Japan . Email: morokuma@fukui.kyoto-u.ac.jp

## Abstract

Thermometers whose emission color gradually changes with temperature are called chameleon emitters. In this study, we discuss the mechanism of the thermosensitivity of the emission color of polymers that contain two lanthanides (Ln^3+^), *e.g.*, [Tb_0.99_Eu_0.01_(hfa)_3_(linker)]_*n*_, where the Ln^3+^(hfa)_3_ complexes (hfa: hexafluoro acetylacetonato) are connected by a phosphine oxide “linker” molecule. First, the difference in the thermosensitivities of the emissions from Tb^3+^ and Eu^3+^ are discussed. With increasing temperature, the green-emission intensity from Tb^3+^ decreases whereas the red-emission intensity from Eu^3+^ does not change. This was found to originate from the different reaction barriers for the quenching of the Ln^3+^ excited state *via* the intersystem crossing (ISC) between the hfa-centered triplet state and the ground state. Next, the excitation energy transfer (EET) from Tb^3+^ to Eu^3+^ is discussed. Although the direct EET between Ln^3+^ atoms is negligible because of the long distance between them, stepwise EET is found to occur *via* the linker-centered triplet state with a reasonable barrier. Thus, we propose a new idea—thermosensitivity can be controlled by the linker as well as by the ligand (hfa). To confirm the role of the linker, four phosphine oxides were examined. The thermosensitivity dependence on the linker is validated *via* experimental measurements.

## Abstract 2

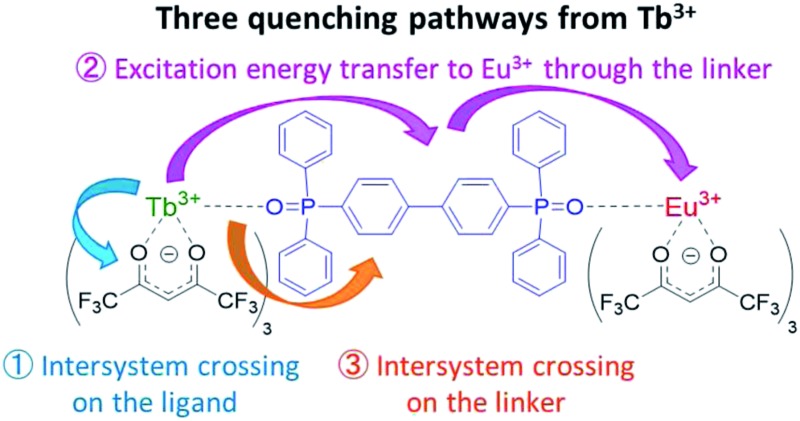
Thermosensitivity of emission intensity in a polymer comprised Tb^3+^ and Eu^3+^ can be controlled by the energy level of the organic linker-centered triplet state as well as that of the ligand-centered triplet state.

## Introduction

Lanthanide (Ln) compounds are widely used as optical materials and sensors because they show bright visible luminescence originating from intra-4f^*N*^ transitions.^1–7^ Typical Ln luminescent materials comprise two parts—photon antenna ligands, which absorb light, and Ln trications (Ln^3+^), which emit light. The energy levels of the Ln^3+^ excited states are independent of the surroundings; therefore, to adjust the emission intensity or intensity dependence, the excited states of the photon antenna need to be adjusted.

**Fig. 1:**
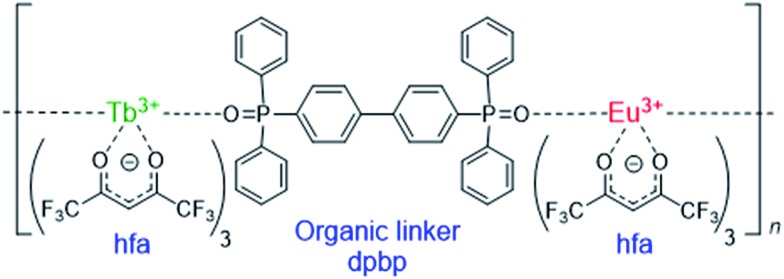
Chameleon thermometer [Ln(hfa)_3_(dpbp)]_*n*_ (Ln = Eu and Tb).

Recently, luminescent materials that comprise more than two Ln^3+^ ions have gained attention as color-tunable light emitters,^8–10^ sensors,^11–14^ and photon up- and down-convertors.^15–17^ One of the most attractive functional materials is the “chameleon” thermometer, whose emission color changes with temperature. Fig. 1 shows a polymeric chameleon thermometer [Tb_0.99_Eu_0.01_(hfa)_3_(dpbp)]_*n*_ (hfa: hexafluoro acetylacetonato, dpbp: 4,4′-bis(diphenylphosphoryl)biphenyl), whose emission color gradually changes from green to yellow to red as temperature increases. Note that [Tb_0.99_Eu_0.01_(hfa)_3_(dpbp)]_*n*_ has drawn attention because of its high quantum yield; thermostability; and wide applicability to fluid dynamics, aeronautical engineering, environment engineering, and energy technology.^18^ The chameleon thermometer comprises Tb^3+^ and Eu^3+^ ions, which emit green and red light, respectively, photon antenna ligands, and phosphine oxide “linker” molecules (dpbp). The hfa ligand has been widely used as a photon antenna for various Ln^3+^ compounds,^19,20^ and Tb^3+^ complexes with hfa ligands have been used in thermometers whose green-emission intensities decrease with increase in temperature.^21–23^

The dependence of emission color on temperature has been attributed to the difference between the emission intensities of Tb^3+^ and Eu^3+^.^18^ The thermosensitivities (the ratios of the decrease in emission intensity per 1 K) of [Tb(hfa)_3_(dpbp)]_*n*_ and [Eu(hfa)_3_(dpbp)]_*n*_ were 0.64% K^–1^ and <0.05% K^–1^, respectively, in the temperature range of 200–300 K.^18^ This means that the emission intensity of Tb^3+^ decreases as the temperature increases whereas that of Eu^3+^ is almost independent of the temperature. Therefore, the chameleon emitter comprising 99% Tb^3+^ and 1% Eu^3+^, *i.e.*, [Tb_0.99_Eu_0.01_(hfa)_3_(dpbp)]_*n*_, shows a green emission from Tb^3+^ at low temperatures. As the temperature increases, the relative intensity of the red emission increases because of the decrease in the green emission.

If there is no interaction between Tb^3+^ and Eu^3+^, the thermosensitivity of the green emission from Tb^3+^ should be the same for [Tb_0.99_Eu_0.01_(hfa)_3_(dpbp)]_*n*_ and [Tb(hfa)_3_(dpbp)]_*n*_. However, the thermosensitivity of the green emission was 0.83% K^–1^ for [Tb_0.99_Eu_0.01_(hfa)_3_(dpbp)]_*n*_; this value is larger than that for [Tb(hfa)_3_(dpbp)]_*n*_ (0.64% K^–1^).^18^ This indicates that excitation energy transfer (EET) takes place from Tb^3+^ to Eu^3+^ (as well as to the hfa ligand) in the chameleon thermometer. In previous theoretical studies, EET between Ln^3+^ atoms has been discussed on the basis of direct EET mechanisms such as the Dexter and Förster mechanisms,^24,25^ and this can occur only at relatively short distances. For instance, Malta reported that the EET rate between two Ln^3+^ (Ln = Yb) ions with 10 Å distance took place mainly through the quadrupole–quadrupole coupling, however, it is an order of magnitude smaller than that of Ln^3+^ f–f emission.^24^ In the case of the chameleon thermometer,^18^ the distance between two Ln^3+^ ions is 13.6 Å; this means that the direct EET from Tb^3+^ to Eu^3+^ does not affect the quenching of Tb^3+^.

To design luminescence materials and sensors that comprise more than two Ln compounds, an understanding of their emission and quenching mechanisms is indispensable. In this study, we discuss the reason for the difference in the thermosensitivities of Tb^3+^ and Eu^3+^ and the mechanism of the EET from Tb^3+^ to Eu^3+^ using computational calculations. On the basis of this theoretical investigation, a new idea to control the thermosensitivity of chameleon thermometers is proposed. This proposed method is then validated *via* experimental measurements.

## Theoretical methods

### Describing the potential energy surfaces (PESs) of Ln^3+^ complexes

**Fig. 2:**
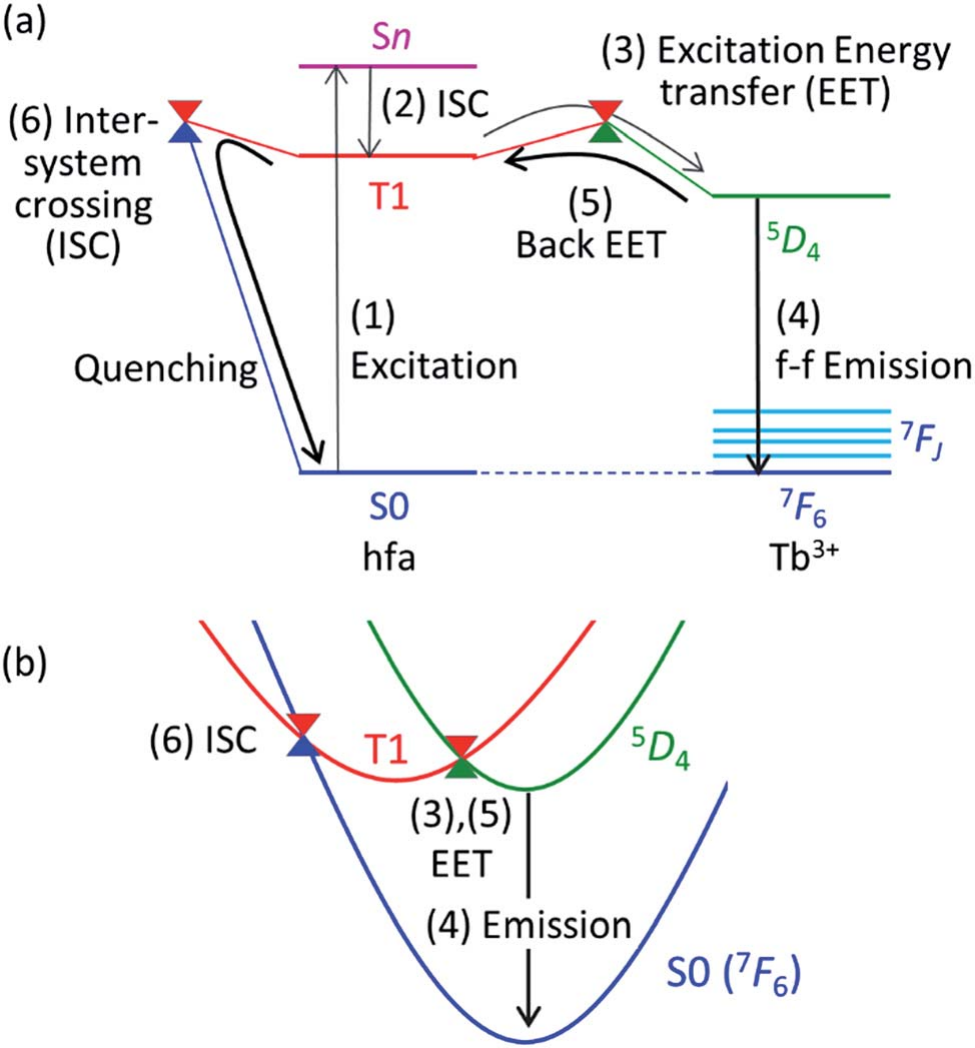
Emission and quenching mechanisms of Tb^3+^ coordinated by hfa ligands shown in the Jablonski diagram (a) and the schematic potential energy surfaces (PESs) (b). S_*n*_ (or T_1_) and ^2*S*+1^L_*J*_ represent the electronic states of hfa and Tb^3+^, respectively.

Theoretical molecular design of luminescent materials on the basis of the information on crossing points (minimal seams of crossing or conical intersections) has been reported for some organic molecules.^26–30^ For Ln^3+^ compounds, however, no study has computed and discussed the crossing points because of the difficulty of *ab initio* calculations for the excited states of Ln^3+^ compounds. To overcome this problem, we applied a reasonable approximation—the energy shift method.^31^ In this section, the approximation is explained on the basis of the Jablonski diagram shown in Fig. 2(a), which shows the possible emission and quenching mechanisms of Ln^3+^ (=Tb^3+^ and Eu^3+^) with hfa ligands. The emission process starts with ligand-centered excitation (1) from the singlet ground state (S_0_) to a singlet excited state (S_*n*_), followed by an intersystem crossing (ISC) (2) from S_*n*_ to the lowest triplet state (T_1_). Next, spin-allowed EET (3) from the ligand T_1_ to the Ln^3+^-centered 4f^*N*^ excited state (^5^D_*J*_) occurs, and then Ln^3+^ emits light *via* an f–f transition (4). The mechanism of quenching of Ln^3+^ can also be understood from Fig. 2(a). The lifetime of the ^5^D_*J*_ state is long because of the parity-forbidden f–f transition. Thus, the quenching process, which starts with a backward EET (5) from the Ln^3+^-centered 4f^*N*^ excited state (^5^D_*J*_) to the ligand T_1_ and is followed by an ISC from ligand T_1_ to S_0_ (6), can take place when the reaction barriers of these two steps are sufficiently low. To evaluate the reaction barrier for the quenching process, the energy levels of the minimal crossing points between two potential energy surfaces (PESs) for both (5) and (6) need to be evaluated.

Thus, we need to describe the three PESs shown in Fig. 2(b)—the ground state, the ligand-centered T_1_ state, and the Ln^3+^-centered excited (^5^D_*J*_) state. To compute these PESs (with a reasonable computational cost), we focus on the character of the intra-4f^*N*^ transition states. The PES of the Ln^3+^-centered excited state (^5^D_*J*_) has a shape nearly identical to that of the ground state, and the excitation energy is independent of the environment because the 4f electrons are shielded from outside by the closed-shell 5s and 5p electrons. Therefore, the PES of ^5^D_*J*_ is described by that of the ground state, corrected by an “energy shift” (for details of the energy shift approximation, see ref. 31). For determining the energy-shift parameters of the ^5^D_*J*_ state, we used the experimental excitation energies of aqueous Ln^3+^, *i.e.*, 580 nm and 490 nm for ^5^D_0_ of Eu^3+^ and ^5^D_4_ of Tb^3+^, respectively.^3^ Using this scheme, the electronic states of Ln^3+^ become identical (^7^F_*J*_) for all the three states. Thus, Ln^3+^ can be described using the Stuttgart–Dresden large-core relativistic effective core potentials (RECP),^36^ in which the 5s, 5p, 5d, and 6s electrons are explicitly considered and the 4f electrons are included in the RECP. Therefore, for the three states shown in Fig. 2(b), only the lowest singlet (S_0_) and the lowest triplet (T_1_) states need to be computed explicitly using a conventional ground-state energy calculation method. This approximation is applicable to model complexes for chameleon polymers that comprise Tb^3+^ and Eu^3+^ (details are given in ESI†).

### Computational details

All the PESs in this study were calculated using the ONIOM method,^32^ in which the high- and low-level regions were described using the density functional theory (DFT) with the ωB97XD functional,^33^ and the molecular mechanics (MM) with the UFF force field parameters,^34^ and the QEq method.^35^ The basis set for the high-level region was the (8s7p6d5f2g)/[6s5p5d3f2g] RECP basis set^36,37^ for Ln^3+^ and cc-pVDZ^38^ for others. For Ln(hfa)_3_(tppo)_2_ (Ln = Eu, Tb; tppo: triphenylphosphine oxide) complexes, we carried out full geometry optimizations of local minima (LMs) and minimum structures on the seams of crossing (MSXs) on and between the ground state, hfa-centered triplet state, and Ln^3+^-centered excited state. For the chameleon-model complexes, the geometry optimizations, with freezing of the position of the surroundings, were applied on the basis of the crystal structures^18,39^ (details are given in ESI†). The energy levels of LMs and MSXs of the chameleon-model complexes were also calculated using different DFT functionals as shown in Tables S2 and S3.† Though their energy levels depended slightly on the functional, the magnitude relation was independent of the functional. All optimizations were performed *via* the Global Reaction Route Mapping (GRRM) program,^40,41^ using the energies and energy derivatives computed using the Gaussian09 program.^42^

## Experimental methods

The linker ligands dpbp, dpb, dppcz, and dpbt (dpb: 1,4-bis(diphenylphosphoryl)benzene, dppcz: 3,6-bis(diphenylphosphoryl)-9-phenylcarbazole, dpbt: 4,4′-bis(diphenylphosphoryl)bithiophene) were prepared as described in ref. 39. The ligands (dpbp: 0.44 g, dpb: 0.38 g, dppcz: 0.51 g, dpbt: 0.45 g, 1 eq.) were dissolved in methanol (30 mL). Tb(hfa)_3_(H_2_O)_2_ (0.65 g, 0.99 eq.) and Eu(hfa)_3_(H_2_O)_2_ (6.5 mg, 0.01 eq.) were dissolved in methanol (30 mL) and were added to each ligand/methanol solution. The mixtures were heated to reflux while stirring for 3 h to give white precipitates of [Tb_0.99_Eu_0.01_(hfa)_3_(dpbp)]_*n*_, [Tb_0.99_Eu_0.01_(hfa)_3_(dpb)]_*n*_, [Tb_0.99_Eu_0.01_(hfa)_3_(dppcz)]_*n*_, and [Tb_0.99_Eu_0.01_(hfa)_3_(dpbt)]_*n*_. The precipitates were filtered, washed with methanol and chloroform several times, and dried *in vacuo* (details are given in ESI.†)

## Results and discussion

### Reason for the difference in the thermosensitivities of the emission intensities of Tb^3+^ and Eu^3+^

**Fig. 3:**
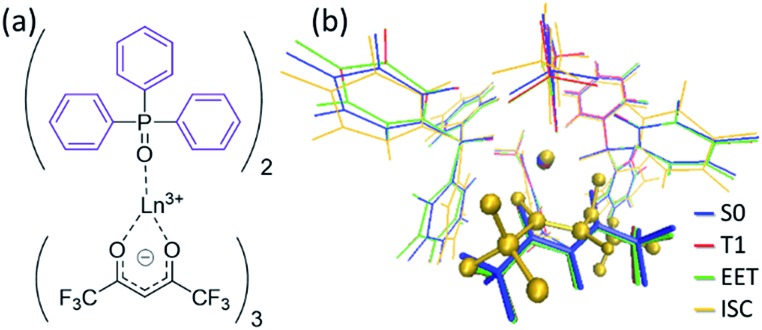
(a) Chemical structure and (b) the optimized geometries of the Tb(hfa)_3_(tppo)_2_ complex. Blue, red, green, and yellow in (b) are the LMs on S_0_, T_1_, and the MSXs for the EET and ISC processes, respectively. Geometrical optimizations were carried out using the ONIOM(ωB97XD:UFF) method, in which the phenyl groups of tppo (purple in (a)) and others (black in (a)) were treated as the low- and high-level regions, respectively.

**Fig. 4:**
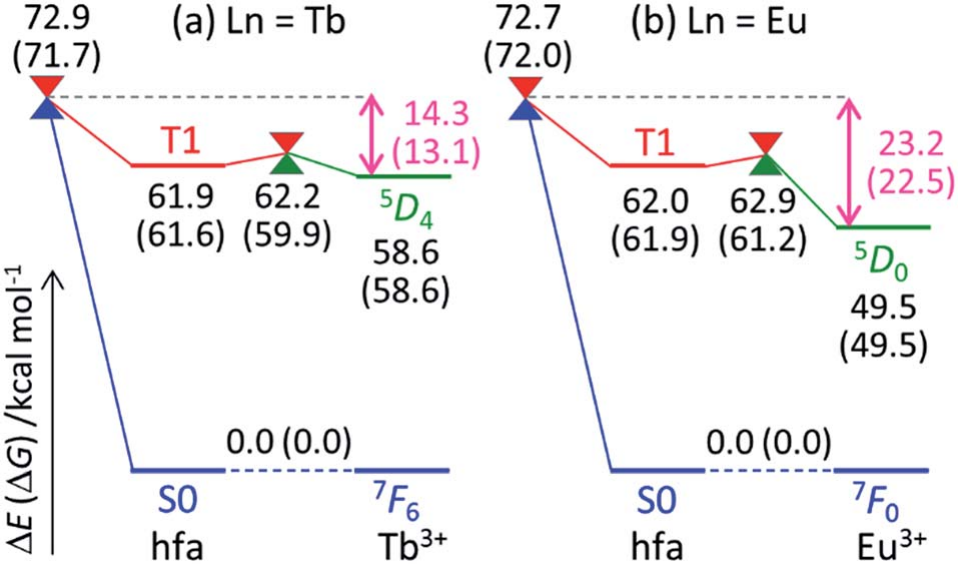
Electronic and Gibbs free energies (in parentheses; kcal mol^–1^) of the LMs and MSXs on and between PESs, respectively, for Ln(hfa)_3_(tppo)_2_ (Ln = (a) Tb, (b) Eu) calculated using the ONIOM(ωB97XD:UFF) level of theory.

To understand the difference in the thermosensitivities of the emission intensities of Tb^3+^ and Eu^3+^, we computed the LMs and MSXs of the model complexes Ln(hfa)_3_(tppo)_2_. Fig. 3 and 4 show the structures and the energy levels of the LMs and MSXs optimized using the ONIOM method. The electronic and Gibbs free-energy levels shown in Fig. 4 are quite similar; therefore, we will discuss the energy levels mainly on the basis of the electronic energies. As shown in Fig. 3(b) and 4(a), the geometry and electronic energy level of the ligand (hfa)-centered T_1_ state (61.9 kcal mol^–1^) are similar to those of the Tb^3+^-centered ^5^D_4_ state (58.6 kcal mol^–1^). This T_1_ energy level is consistent with the experimental energy level of the hfa-centered T_1_ state of Gd(hfa)_3_(H_2_O)_2_ (63.5 kcal mol^–1^).^23^ The MSX between the T_1_ and the ^5^D_4_ states, where the EET between hfa and Tb^3+^ occurs, also has a geometry and energy level similar to those of the abovementioned two states. Thus, forward and backward EET can occur almost without a barrier. Conversely, the electronic energy of the MSX for the ISC from the ligand-centered T_1_ to S_0_ is 14.3 kcal mol^–1^ higher than that of the Tb^3+^-centered ^5^D_4_. Thus, the ISC of the ligand *via* the T_1_/S_0_ MSX is the rate-determining step for the quenching process. Comparing the structures, one of the hfa ligands (bold-faced in Fig. 3(b)) has a bent structure at the T_1_/S_0_ MSX for the ISC, whereas all the hfa ligands are planar at all other LMs and MSX. This means that the excitation is localized on an hfa ligand and that the C=O bending motion of this hfa ligand induces vibrational relaxation.

Next, we focus on the differences between the Tb^3+^ and Eu^3+^ complexes. The geometries of the LMs and MSXs of the Eu^3+^ complex are similar to those of the Tb^3+^ complex, as shown in Fig. S1.† Their energy profiles are also quite similar, except for the energy levels of the Ln^3+^-centered excited states (^5^D_*J*_), as shown in Fig. 4. Thus, the barrier for the quenching process of the Eu^3+^ complex is 8.9 kcal mol^–1^ higher than that of the Tb^3+^ complex; this makes quenching the red emission from Eu^3+^ difficult. To compare the timescales for the emission and quenching processes, their rate constants were estimated. The rate constant of the Tb^3+^ emission was estimated using the inverse of the experimental emission lifetime, *i.e.*, 0.8 ms, measured for Tb(hfa)_3_(tppo)_2_ at 80 K.^23^ The rate constants of the quenching processes were evaluated *via* the Gibbs free activation energies (13.1 and 22.5 kcal mol^–1^ for the Tb^3+^ and Eu^3+^ complexes, respectively) using transition state theory.^43–45^ The rate constants of emission are comparable to those of quenching for the Tb^3+^ and Eu^3+^ complexes at 300 K and 500 K, respectively (see Table S1†). These values are consistent with the experimental facts that emission intensity decreases for the Tb^3+^ complex and does not change for the Eu^3+^ complex as the temperature increases in the range of 200–300 K.^18,21–23^

### Mechanism of the EET from Tb^3+^ to Eu^3+^ in the chameleon thermometer

**Fig. 5:**
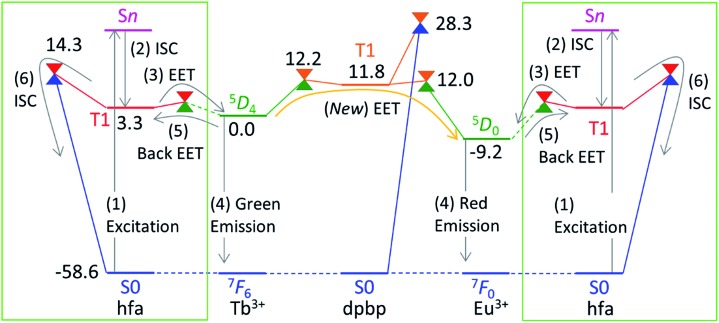
Electronic energy levels (in kcal mol^–1^) of the LMs and MSXs for the model complex of the chameleon thermometer comprising the dpbp linker calculated at the ONIOM(ωB97XD:UFF) level of theory. The energy levels of the hfa-centered T_1_ and MSXs (in the green box) are obtained from Fig. 4. For convenience, the energy zero (^5^D_4_) is shifted from zero (the ground state) in Fig. 4.

Next, we focus on the thermosensitivity of the luminescence of the chameleon emitter.^18^ As mentioned above, direct EET from Tb^3+^ to Eu^3+^ is negligible.^24^ Thus, in order to consider an alternative pathway for the EET from Tb^3+^ to Eu^3+^, we focus on the phosphine oxide “linker” (dpbp in Fig. 1). Fig. 5 shows the electronic energy levels of the LMs and MSXs of the model complex, which were constructed on the basis of the crystal structure and were optimized (see the computational details in ESI†). The early stage of the emission process is the same as that of Tb^3+^ and Eu^3+^ complexes with hfa ligands; the hfa ligand absorbs the light and the EET from hfa-centered T_1_ state to Tb^3+^- or Eu^3+^-centered ^5^D_*J*_ excited state takes place as shown in Fig. 5. Compared with the Tb^3+^-centered ^5^D_4_ reference, the LMs on the linker-centered T_1_ state is 11.8 kcal mol^–1^ higher, and the MSXs between linker-centered T_1_ and Tb^3+^- and Eu^3+^-centered ^5^D_*J*_ are 12.2 and 12.0 kcal mol^–1^ higher, respectively. Thus, the stepwise EET from Tb^3+^ to Eu^3+^ *via* the linker-centered T_1_ state can occur with a reasonable reaction barrier, which is lower than the barrier (at 14.3 kcal mol^–1^) for the quenching *via* an ISC on the hfa ligand. Additionally, we obtained a MSX for the ISC between the linker-centered T_1_ state to the ground state, which is 28.3 kcal mol^–1^ higher than the Tb^3+^-centered ^5^D_4_ state. Toward the ISC, one of the C–H bonds in the phenyl group bends, as shown in Fig. S5.† The effect of the ISC *via* linker T_1_ is almost negligible due to the too high barrier. Thus, there exist two comparable quenching pathways from Tb^3+^—the ISC on hfa and the EET to Eu^3+^.

### Computational prediction of the thermosensitivity of chameleon thermometers comprising different linker molecules

As shown in Fig. 5, the linker-centered T_1_ state is thoroughly involved in the quenching of Tb^3+^. This suggests that the barrier for the quenching of Tb^3+^, *i.e.*, the thermosensitivity of the emission from Tb^3+^, could be controlled by changing the linker molecule. In other words, we may be able to design a thermometer with the desired thermosensitivity by optimizing the energy level of the linker-centered T_1_ state and the ISC on the linker.

**Fig. 6:**
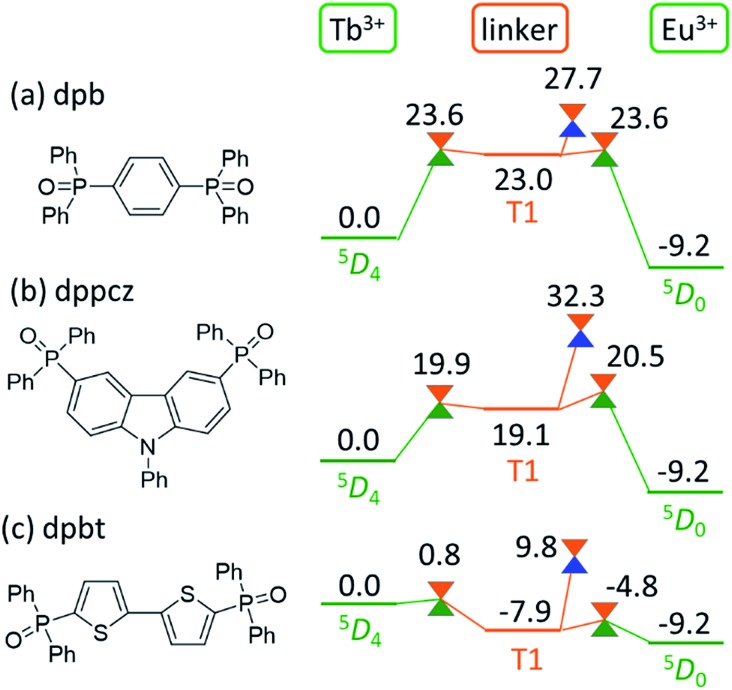
Chemical structures of the linker molecules and electronic energy levels (in kcal mol^–1^) of the LMs and MSXs for the model complexes involving (a) dpb, (b) dppcz, and (c) dpbt. Ln^3+^-centered excited ^5^D_*J*_ states, the linker-centered T_1_ states, and the ground states are shown in green, orange, and blue, respectively.

To confirm this new quenching mechanism, other polymers that comprise three different linker molecules, *i.e.*, [Ln(hfa)_3_(dpb)]_*n*_, [Ln(hfa)_3_(dppcz)]_*n*_, and [Ln(hfa)_3_(dpbt)]_*n*_, were examined. Fig. 6 shows the energy levels of the LMs and MSXs of the model complexes, each involving a linker (dpb, dppcz, or dpbt), Eu(hfa)_3_, Tb(hfa)_3_, and surroundings, whose structures were constructed on the basis of crystal structures and were optimized (as shown in Fig. S6†).^39^ The energy level of the linker-centered T_1_ state is the highest for dpb, followed by dppcz, dpbp, and dpbt, and they are 23.0, 19.1, 11.8, and –7.9 kcal mol^–1^ higher, respectively, than the energy level of the Tb^3+^-centered ^5^D_4_ state. The energy levels and geometries of the MSXs of the EET between Ln^3+^ and the linker are similar to those of the linker-centered T_1_ states, especially when the linker-centered T_1_ state is less stable than ^5^D_4_. Thus, the barrier for the EET from Tb^3+^ to Eu^3+^ is essentially determined by the linker-centered T_1_ state. In the case of the dpbt model complex, the energy levels of the linker-centered T_1_ state and the MSXs for the EET between Ln^3+^ and dpbt are lower than that of ^5^D_4_. Thus, the EET from Tb^3+^ to Eu^3+^ should be an almost barrierless process.

Next, we focus on the MSXs for the ISC from the linker-centered T_1_ state to the ground state. The order of their energy levels is different from that of the T_1_ states. The energy level of the MSX on the linker is the highest for dppcz, followed by dpbp, dpb, and dpbt, and they are 32.3, 28.3, 27.7, and 9.8 kcal mol^–1^ higher, respectively, than the energy level of the Tb^3+^-centered ^5^D_4_ state. These barriers are too high to affect the quenching rate of Tb at room temperature, except for dpbt. The MSX for the ISC on dpbt is lower than that for the ISC on hfa (14.3 kcal mol^–1^). Thus, the quenching of Eu^3+^ and Tb^3+^ in this polymer should occur much faster than in other polymers. Note that the structural changes from the linker-centered T_1_ minimum to the ISC are similar for the model complexes comprising dppcz, dpbp, and dpb; one of the C–H bonds in the phenyl group bends (shown in Fig. S7†). The reason for the instability of the ISC in dppcz can be attributed to the packing effect from the surroundings, because the stability of the ISC for isolated linker molecules is similar (see Fig. S8†).

### Experimental confirmation of the EET mechanism *via* the linker-centered triplet state

**Fig. 7:**
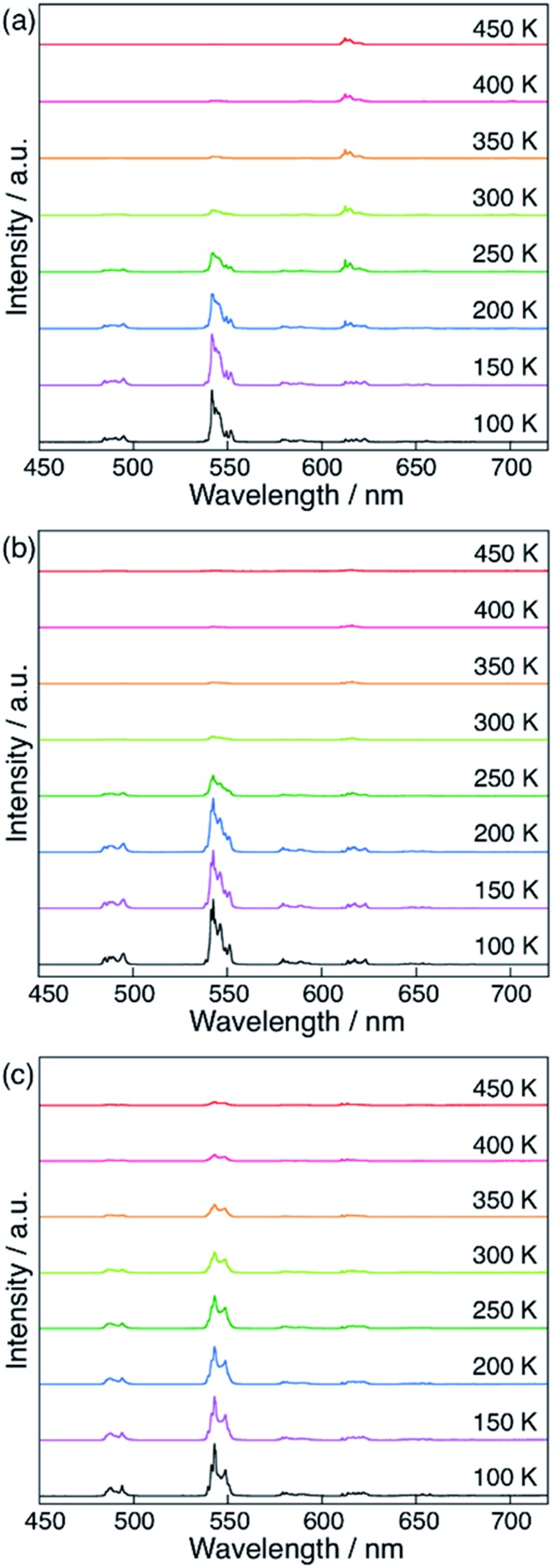
Temperature-dependent emission spectra of (a) [Tb_0.99_Eu_0.01_(hfa)_3_(dpbp)]_*n*_, (b) [Tb_0.99_Eu_0.01_(hfa)_3_(dpb)]_*n*_, and (c) [Tb_0.99_Eu_0.01_(hfa)_3_(dppcz)]_*n*_ in solid state (*λ*_ex_ = 380 nm). The spectra for [Tb_0.99_Eu_0.01_(hfa)_3_(dpbt)]_*n*_ are not shown because they have very weak emission intensities.

Finally, we compared the computational predictions with the experimental thermosensitivities. The three polymers, [Tb_0.99_Eu_0.01_(hfa)_3_(X)]_*n*_ (X = dpb, dppcz, and dpbt), were synthesized and their temperature-dependent emission spectra in the temperature range 100–450 K in the solid state were observed. Fig. 7 shows these spectra for the polymers along with that of the original chameleon thermometer, [Tb_0.99_Eu_0.01_(hfa)_3_(dpbp)]_*n*_. The emission spectrum of [Tb_0.99_Eu_0.01_(hfa)_3_(dpbt)]_*n*_ is not shown in Fig. 7 because no emission was observed. This is consistent with the computational result, which showed barrierless quenching for Tb and a low barrier for the quenching of Eu^3+^ *via* dpbt. The other polymers exhibited different temperature sensitivities. The sensitivities in the temperature range of 200–300 K were 0.83% K^–1^, 0.82% K^–1^, and 0.45% K^–1^ for [Tb_0.99_Eu_0.01_(hfa)_3_(X)]_*n*_, in which X = dpbp, dpb, and dppcz, respectively. Compared to the original chameleon thermometer [Tb_0.99_Eu_0.01_(hfa)_3_(dpbp)]_*n*_, [Tb_0.99_Eu_0.01_(hfa)_3_(dppcz)]_*n*_ showed an inferior temperature sensitivity; this can be attributed to the higher barrier for the EET from Tb^3+^ to Eu^3+^ *via* the linker-centered T_1_ state. [Tb_0.99_Eu_0.01_(hfa)_3_(dpb)]_*n*_ had a slightly inferior temperature sensitivity than the original chameleon thermometer even though the barrier for the EET from Tb^3+^ to Eu^3+^ was 11.3 kcal mol^–1^ higher. The reason of the discrepancy between experiment and theoretical prediction (*i.e.* the high sensitivity of [Tb_0.99_Eu_0.01_(hfa)_3_(dpb)]_*n*_) could be attributed by the mixing of charge transfer to the hfa-centered triplet states. To examine the characters of the triplet excited states, the excited energies of the Franc–Condon region of the model complexes comprising dpbp, dpb and dppcz were calculated using the full-QM time-dependent DFT (TDDFT) method as shown in Fig. S9 and Table S4.† The six lower and the seventh triplet states are the hfa-centered and the linker-centered excited states, respectively. The linker-centered triplet states of the model complexes comprising dpbp, dpb and dppcz are well localized on the linker as shown in Fig. S10,† which confirms the adequacy of our ONIOM calculation scheme for Fig. 5 and 6. Moreover, the hfa-centered triplet states of the model complex comprising dpb are localized on each hfa and the excitation energies are close to the results in Fig. 4. On the other hand, the six lower excited states of the model complexes comprising dpbp and dppcz are about 2–4 kcal mol^–1^ destabilized due to the mixing of charge transfer component from hfa to the linker. Therefore, the high thermosensitivity of [Tb_0.99_Eu_0.01_(hfa)_3_(dpb)]_*n*_ could be attributed to the faster quenching *via* the ISC on hfa ligands.

As shown above, it was confirmed that thermosensitivity depends on the linker molecules. This phenomenon was mainly explained by the difference in the energy levels of the linker-centered T_1_ state. Although the numerical estimation of the temperature sensitivities is difficult by our calculation scheme due to the mixing of the charge transfer to the hfa-centered excited states, we can accelerate the development of the chameleon thermometers by focusing on the linkers whose T_1_ energies are higher than the energy level of the emissive state of Tb^3+^ (^5^D_4_). The effect of the Tb/Eu ratio was also not considered in our computational models shown in Fig. 5 and 6, however, we expect that the effect of the EET from Tb^3+^ to Eu^3+^ could increase as the amount of Eu^3+^ increases. In fact, the thermosensitivity of [Tb_(1–*n*)_Eu_*n*_(hfa)_3_(dpbp)]_*n*_ with a different Tb^3+^/Eu^3+^ ratio was observed in a previous study.^46^ The effect of the EET from Tb^3+^ to Eu^3+^ increased as the amount of Eu^3+^ increased.

## Conclusions

In this study, we observed that there are three quenching pathways from the Tb^3+^-centered excited state in chameleon-type polymers. Moreover, we found that the thermosensitivity of the emission from Tb^3+^ can be controlled by changing the reaction barriers for the three quenching pathways. The first quenching pathway is through an ISC from the β-diketone ligand-centered T_1_ state to the ground state.^31^ The second is through the stepwise EET from the excited state in Tb^3+^ to that in Eu^3+^; this pathway is determined mainly by the linker-centered T_1_ state. The third is through an ISC from the linker-centered T_1_ state to the ground state. The experimental linker-dependence of thermosensitivity confirms the contributions of the newly proposed linker-centered T_1_ quenching pathway. Our proposed strategy to use linker T_1_ for designing a chameleon thermometer should be applicable not only to polymers but also to other materials, such as metal–organic frameworks and nanoclusters.^11–14^
