# Supplementary material for: Organic linkers control the thermosensitivity of the emission intensities from Tb(iii) and Eu(iii) in a chameleon polymer
Source: Chem Sci. 2016 Aug 25;8(1):423–9. doi: 10.1039/c6sc03006h (PMC5365065; doi:10.1039/c6sc03006h)
Supplement: Supplementary file 1 [file SC-008-C6SC03006H-s001.pdf]

Supporting Information for:

Organic linkers control the thermosensitivity of the emission intensities from Tb(III) and Eu(III) in a chameleon polymer

Miho Hatanaka,[*,1,2] *Yuichi Hirai,*[3] *Yuichi Kitagawa,*[3] *Takayuki Nakanishi,*[3]

Yasuchika Hasegawa,[*,3] *Keiji Morokuma*[*,4]

[1] Department of Chemistry, Faculty of Science and Engineering, Kindai University, Higashi-Osaka, Osaka 577-8502, Japan.

[2] PRESTO, Japan Science and Technology Agency (JST), 4-1-8 Honcho, Kawaguchi, Saitama 332-0012, Japan.

[3] Faculty of Engineering, Hokkaido University, Sapporo, Hokkaido 060-8628, Japan.

[4] Fukui Institute for Fundamental Chemistry, Kyoto University, Kyoto 606-8103, Japan.

*Email: hatanaka@chem.kindai.ac.jp (MH); hasegaway@eng.hokudai.ac.jp (YH); morokuma@fukui.kyoto-u.ac.jp (KM)

Contents

1. Computational details S2
   1.1. Optimized geometries of $Eu(hfa)_3(tppo)_2$
   1.2. Rate constants for emission and quenching
   1.3. Model complex for the chameleon $[Ln(hfa)_3(dpbp)]_n$ (Ln = Eu and Tb)
   1.4. Model complexes with different linker molecules (dpb, dppcz, dpbt)
2. Experimental details S9
   2.1. Analyses of the synthesized polymers
   2.2. Optical measurements
3. Cartesian coordinates S10

1. Computational details

1.1. Optimized geometries of Eu(hfa)$_3$(tppo)$_2$

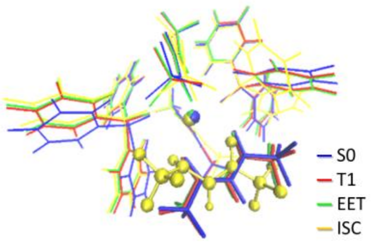

Figure S1. The geometries of the critical points of the Eu(hfa)$_3$(tppo)$_2$ complex obtained by the ONIOM(ωB97XD:UFF) method. Blue, red, green, and yellow are the local minima on (S0, $^7F_0$ or $^5D_0$), (T1, $^7F_0$), and the minimum energy crossing points for the EET and ISC processes, respectively.

1.2. Rate constants for emission and quenching

According to the transition state theory, the rate constant k can be written

$$k = \gamma \frac{k_B T}{h} \exp\left(-\frac{\Delta G^{\ddagger}}{RT}\right) \quad (1)$$

where k_B is Boltzmann's constant, h is Plank's constant, T is temperature, R is the gas constant, $\Delta G^{\ddagger}$ is the free energy of activation, and γ is the transmission coefficient which is often taken as $\gamma = 1$. The spin-orbit effect needs to be considered because the spin multiplicity changes at the T1/S0 crossing point ((6) in Fig. 2). The rate constant (1), however, can be approximately treated like a spin-allowed transition because Ln^{3+} has a large spin-orbit coupling constant. Table S1 shows the quenching rate constants estimated for $\gamma = 1$ and $\Delta G^{\ddagger}$ = 13.1 and 22.5 kcal mol^{-1} for Tb^{3+} and Eu^{3+} complexes, respectively.

Table S1. Rate constants [s^{-1}] of quenching for Ln^{3+}(hfa)$_3$(tppo)$_2$

Temperature [K]	Tb^{3+} $\Delta G^{\ddagger}$ = 13.1 [kcal mol^{-1}]	Eu^{3+} $\Delta G^{\ddagger}$ = 22.5 [kcal mol^{-1}]
100	5×10^{-17}	1×10^{-37}
200	2×10^{-2}	1×10^{-12}
300	2×10^{3}	3×10^{-4}
400	6×10^{5}	4×10^{0}
500	2×10^{7}	5×10^{3}

The rate constant for Tb^{3+} emission is 1×10^3 s^{-1}, which is estimated from the experimental lifetime of Tb(hfa)$_3$(tppo)$_2$ at 80 K (0.8 ms). That for Eu^{3+} is 1×10^3 s^{-1}, which is estimated from the experimental lifetime of [Eu(hfa)$_3$(dpbp)] at 300 K (0.85 ms). (Note that the barrier for quenching in Eu^{3+} with hfa is large enough, thus the lifetime at 300 K is applicable to estimate k.)

As shown above, the quenching rate constants at 300 K for Tb^{3+} and 500 K for Eu^{3+} are comparable to the emission rate constants.

1.3. Model complex for the chameleon [Ln(hfa)$_3$(dpbp)]$_n$ (Ln = Eu and Tb)

The shapes of the PESs of the ground state and Ln^{3+}-centered excited states for a system including two Ln^{3+} are nearly identical when the distance between Ln^{3+} are enough long to ignore their interaction. Thus, in the case of the chameleon polymer, in which the distance between Tb^{3+} and Eu^{3+} is 13.6 Å, the PESs of Tb^{3+}- and Eu^{3+}-centered excited states can be described by that of the ground state corrected by the energy shift parameters, 580 and 490 nm, respectively. Figure S2 shows the schematic image of the PESs of the ground and excited states related to the EET from Tb^{3+} to Eu^{3+}.

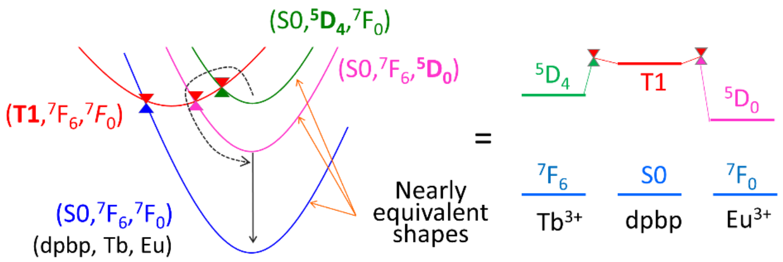

Figure S2. Schematic illustration of the PESs for the ground and excited states in the chameleon polymer. The ground state and Tb^{3+}-, Eu^{3+}-, dpbp-centered excited states are in blue, green, pink, and red, respectively. The notation (S0, $^7F_6$, $^7F_0$) represents the electronic states of the dpbp, Tb, and Eu, are S0, $^7F_6$, and $^7F_0$, respectively.

The chemical structure of the chameleon model complex is shown in Figure S3. The initial structure was obtained by the crystal structure (CCDC: 855863). The electronic energies were evaluated by two ONIOM schemes. The high-level regions of the ONIOM schemes (I) and (II) are shown in green and red, respectively. The density functional theory (DFT) method with the same basis set in the section 1.1 and the MM method with the UFF force parameters[S2] and the QEq charges[S3] were used for the high- and low-level calculations. The atoms in low-level region were fixed during the geometry optimization.

Figure S4 shows the electronic energy levels of the critical points calculated by the ONIOM(B3LYP:UFF) schemes (I) and (II) as well as the ONIOM(ωB97XD:UFF)-(I). (Figure 4 in the main text shows the results of the ONIOM(ωB97XD:UFF)-(I). Their optimized structures are shown in Figure S5.) The energy differences among the critical points by the ONIOM (I) reproduced those by the ONIOM (II). Thus, only the linker molecule was treated as the high-level region for the calculations of other model complexes.

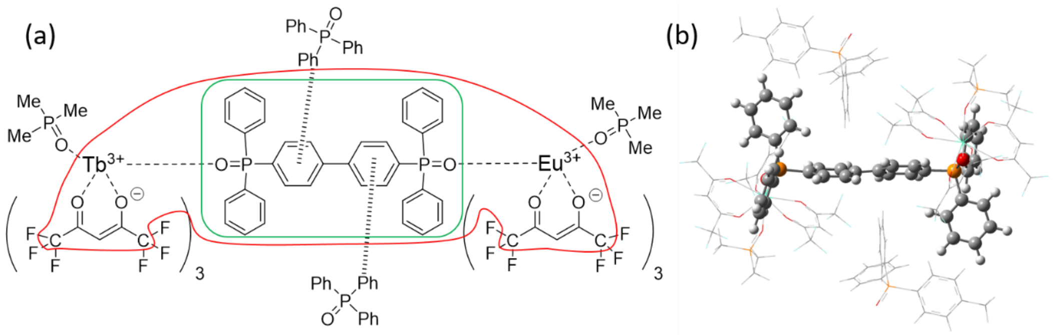

Figure S3. (a) Chemical structure and the ONIOM scheme for the model complex of the Chameleon polymer. The high-level regions of the ONIOM schemes (I) and (II) are in green and red, respectively. (b) The optimized structure on the ground state (S0) by the ONIOM(ωB97XD:UFF)-(I) scheme. The high- and low-level regions are shown in ball/stick and in line, respectively.

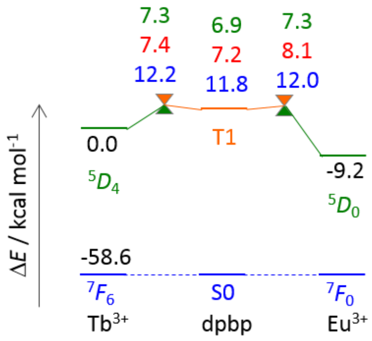

Figure S4. Electronic energy levels (kcal mol^{-1}) of the critical points of the model complex show in Figure S2. Electronic energies calculated by the ONIOM(B3LYP:UFF)-(I) , (B3LYP:UFF)-(II), and (ωB97XD:UFF)-(I) are shown in green, red, and blue, respectively.

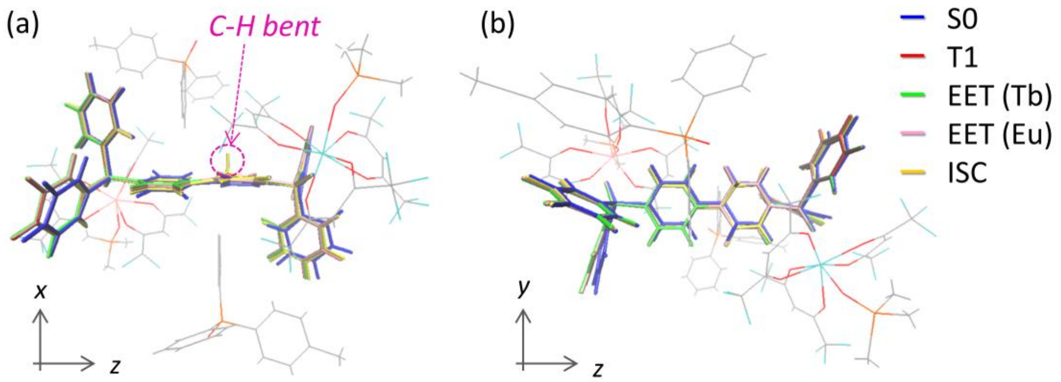

Figure S5. (a) Top and (b) side views of the critical points of the Chameleon model complex calculated by the ONIOM(ωB97XD:UFF) method. Blue, red, green, pink, and yellow are the local minima on the ground state, dpbp-centered T1, and the minimum energy crossing points for the EET between T1 and $^5D_4$ of Tb, the EET between T1 and $^5D_0$ of Eu, and ISC processes, respectively.

1.3. Model complexes with different linker molecules (dpb, dppcz, dpbt)

The chemical structures of the model complexes with the linker molecules (dpb, dpppcz, and dpbt) are shown in Figure S6. The initial structures were obtained by the crystal structures (CCDC: 855862, 855865, and 855864).[34] The electronic energies were evaluated by the ONIOM(ωB97XD:UFF) scheme where the linker molecule and others are treated as high- and low-level regions, respectively. The geometrical optimizations of the critical points (shown in Figure S7) were carried out with fixing the atoms in the low-level regions.

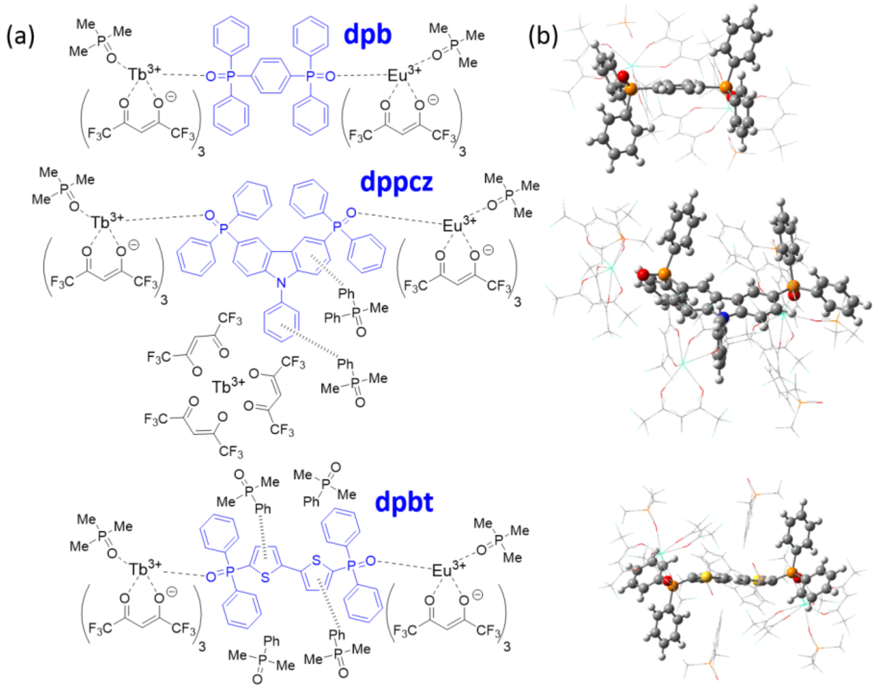

Figure S6. (a) Chemical structures and (b) the optimized structures on the ground states for the model complexes with the linker molecules, dpb, dppcz, and dpbt. The high-level regions for the ONIOM(ωB97XD:UFF) calculations are shown in (a) blue and in (b) ball/stick.

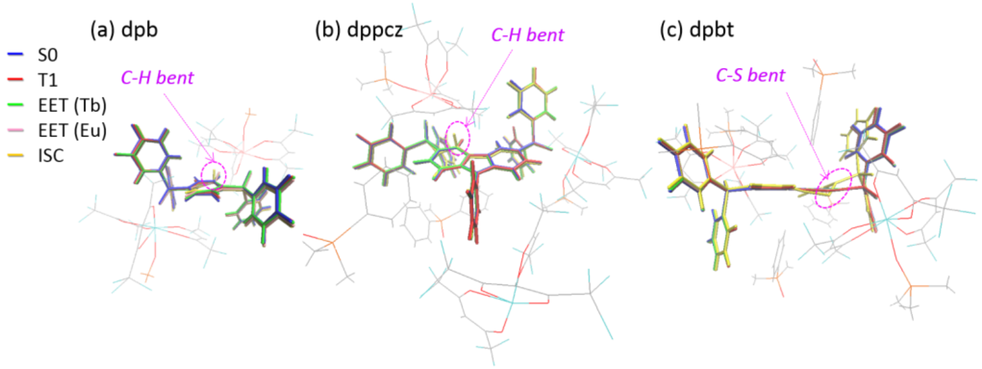

Figure S7. Structural changes of the linker molecules in the model complexes shown in Figure S6. Blue, red, green, pink, and yellow are the local minima on the ground state, the linker-centered T1, and the minimum energy crossing points for the EET between T1 and $^5D_4$ of Tb, the EET between T1 and $^5D_0$ of Eu, and ISC processes, respectively.

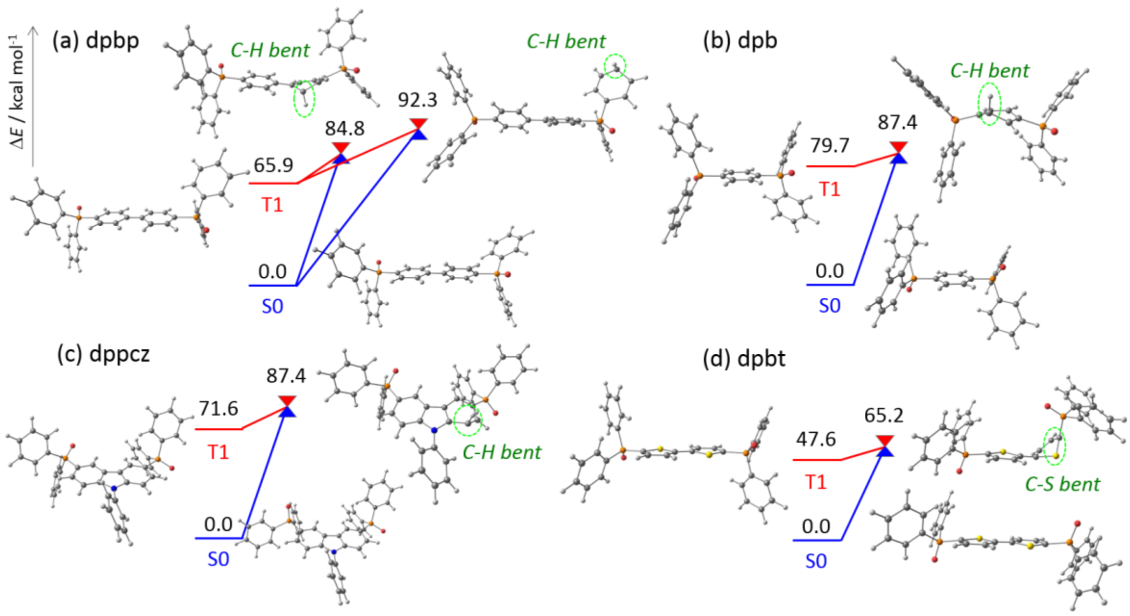

Figure S8. Potential energy profiles (in kcal mol^{-1}) of the isolated linker molecules calculated by the ωB97XD/cc-pVDZ level of theory. Blue and red are S0 and T1 states, respectively.

Table S2. Energy levels (kcal mol^{-1}) of the linker-centered T1 of model complexes optimized at ONIOM(DFT:UFF) level using different DFT functionals (The energy zero is $^5D_4$. See Figure 5)

functionals	dpbp-model	dpb-model	dppcz-model	dpbt-model
B3LYP	6.9	21.0	14.0	-11.5
BH&HLYP	7.7	19.2	15.3	-11.8
LC-ωPBE	9.9	18.9	15.8	-8.2
CAM-B3LYP	9.9	21.8	17.3	-9.3
ωB97XD	11.8	23.0	19.1	-7.9
M11	14.3	24.9	20.8	-4.7
M062X	15.2	27.1	22.3	-3.3

Table S3. Energy levels (kcal mol^{-1}) of the MSXs between the ground state and the linker-centered T1 of model complexes optimized at ONIOM(DFT:UFF) level using different DFT functionals (The energy zero is $^5D_4$. See Figure 5)

functionals	dpbp-model	dpb-model	dppcz-model	dpbt-model
B3LYP	27.0	27.1	32.3	3.3
BH&HLYP	22.9	23.0	28.1	2.8
LC-ωPBE	21.7	21.1	24.8	4.1
CAM-B3LYP	26.5	26.4	31.1	8.5
ωB97XD	28.3	27.7	32.3	9.8
M11	28.9	27.8	32.0	11.2
M062X	31.4	30.8	35.2	12.5

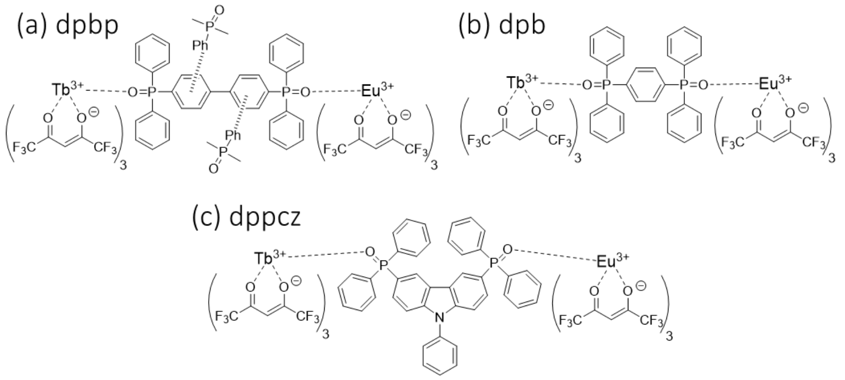

Figure S9. Chemical structures of the model complexes for the full-QM TDDFT calculations comprising dpbp (a) dpb (b) and dppcz (c).

Table S4. Energy levels (kcal mol^{-1}) of the *N*-th triplet states of the model complexes in Figure S9 calculated with TDDFT method.[1] (The energy zero is $^5D_4$. See Figure 5)

N-th triplet state	dpbp	dpb	dppcz
1	5.7	2.1	4.3
2	5.9	2.2	4.6
3	5.9	2.7	5.2
4	6.0	2.8	5.8
5	6.1	4.0	6.8
6	6.2	4.1	7.3
7	12.4	23.0	15.6

[1] Full-QM TDDFT calculations were carried out with ωB97XD method. The large-core RECP and (8s7p6d5f2g)/[6s5p5d3f2g] RECP basis set were used for Tb^{3+} and Eu^{3+} and cc-pVDZ were used for others. The structures are based on the optimized structure of the ground state with ONIOM(ωB97XD:UFF) shown in Figures 5 and 6.

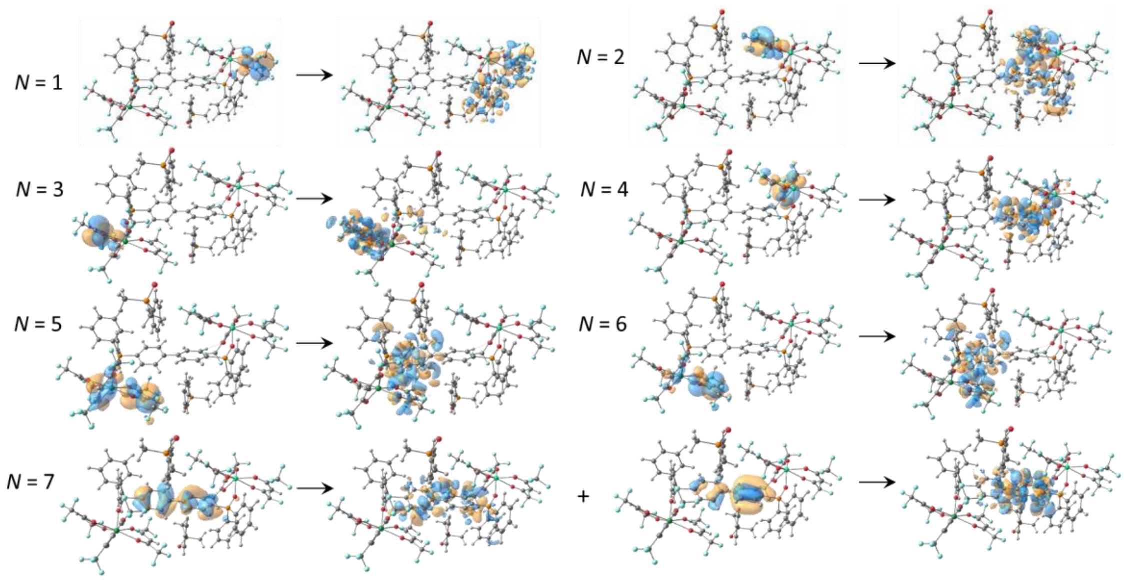

Figure S10(a). Natural transition orbitals of the *N*-th (*N* = 1-7) triplet states calculated using the full-QM TDDFT method for the model complex comprising dpbp shown in Figure S9(a). (isovalue = 0.03)

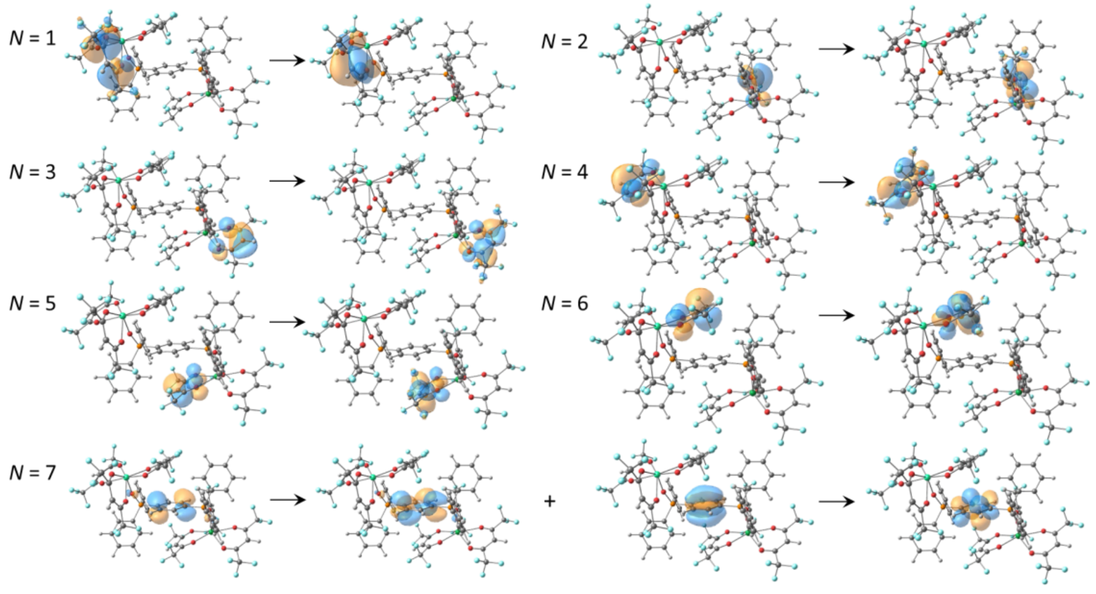

Figure S10(b). Natural transition orbitals of the *N*-th (*N* = 1-7) triplet states calculated using the full-QM TDDFT method for the model complex comprising dpb shown in Figure S9(b). (isovalue = 0.03)

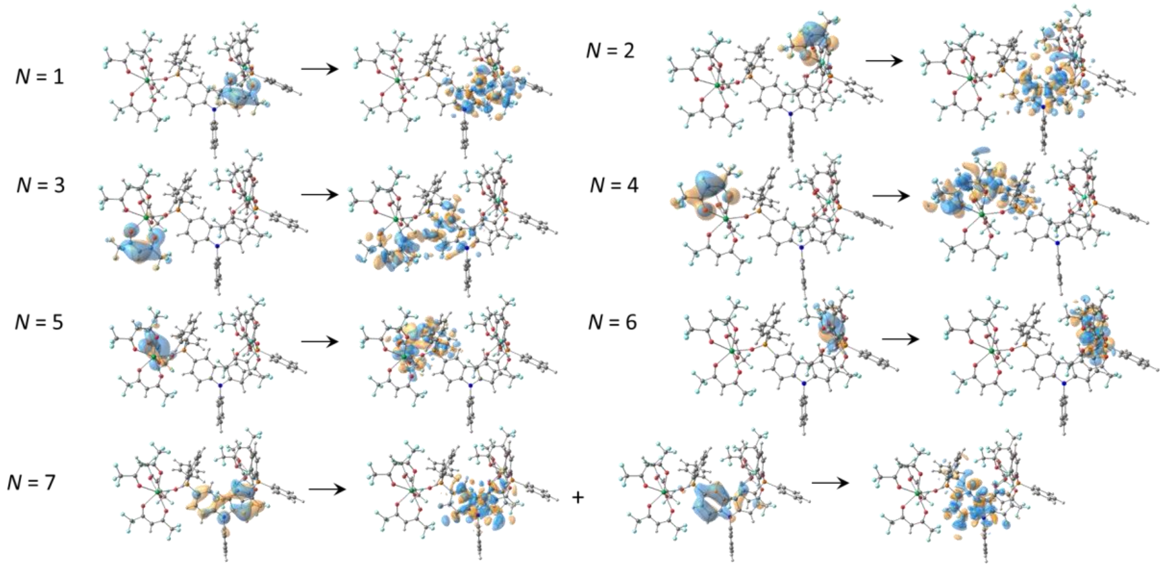

Figure S10(c). Natural transition orbitals of the *N*-th (*N* = 1-7) triplet states calculated using the full-QM TDDFT method for the model complex comprising dppcz shown in Figure S9(c). (isovalue = 0.03)

2. Experimental details

2.1. Analyses of the synthesized polymers.

Infrared spectra were measured using a JASCO FT/IR–4600 spectrometer. Elemental analyses were performed on an Exeter Analytical CE440. ESI-MS and FAB-MS spectra were recorded on a JEOL JMS-T100LP and a JMS-700TZ. (Note that the experimental data for $[Tb_{0.99}Eu_{0.01}(hfa)_3(dpbp)]_n$ has been already published in Ref. 18. The X-ray structures of $[Eu(hfa)_3(L)]_n$ (L = dpb, dppcz, dpbt, and dpbp) have been published in Ref. 39.)

$[Tb_{0.99}Eu_{0.01}(hfa)_3(dpb)]_n$: Yield 0.46 g (46%; for monomer). IR (KBr) 3070 (st, C-H), 1656 (st, C=O), 1258-1147 (st, C-F), 1128 (st, P=O) cm^{-1}. ESI-Mass (*m*/*z*) = 1051.01 $[Tb_{0.99}Eu_{0.01}(hfa)_2(dpb)]^+$, 2309.03 $[(Tb_{0.99}Eu_{0.01})_2(hfa)_5(dpb)]^+$. Anal. Calcd for $[C_{45}H_{27}Tb_{0.99}Eu_{0.01}F_{18}O_8P_2]_n$: C, 42.95; H, 2.16%. Found: C, 42.90; H, 2.00%.

$[Tb_{0.99}Eu_{0.01}(hfa)_3(dppcz)]_n$: Yield 0.28 g (25%; for monomer). IR (KBr) 3064 (st, C-H), 1656 (st, C=O), 1256-1147 (st, C-F), 1128 (st, P=O) cm^{-1}. FAB-Mass (*m*/*z*) = 1216.1 $[Tb_{0.99}Eu_{0.01}(hfa)_2(dppcz)]^+$. ESI-Mass (*m*/*z*) = 2640.12 $[(Tb_{0.99}Eu_{0.01})_2(hfa)_5(dppcz)]^+$. Anal. Calcd for $[C_{57}H_{34}Tb_{0.99}Eu_{0.01}F_{18}NO_8P_2]_n$: C, 48.09; H, 2.41%. Found: C, 47.89; H, 2.22%.

$[Tb_{0.99}Eu_{0.01}(hfa)_3(dpbt)]_n$: Yield 0.66 g (61%; for monomer). IR (KBr) 3070 (st, C-H), 1656 (st, C=O), 1256-1142 (st, C-F), 1125 (st, P=O) cm^{-1}. ESI-Mass (*m*/*z*) = 1139.58 $[Tb_{0.99}Eu_{0.01}(hfa)_2(dpbt)]^+$, 2486.89 $[(Tb_{0.99}Eu_{0.01})_2(hfa)_5(dpbt)]^+$. Anal. Calcd for $[C_{47}H_{27}Tb_{0.99}Eu_{0.01}F_{18}O_8P_2S_2]_n$: C, 41.92; H, 2.02%. Found: C, 41.78; H, 1.83%.

2.2. Optical measurements.

Temperature-dependent emission spectra from 100 K to 450 K in solid state for the Chameleon polymers (Figure 7) were recorded on a HORIBA Fluorolog-3 spectrofluorometer with a cryostat (Thermal Block Company, SA-SB245T) and a temperature controller (Oxford, Instruments, ITC 502S), and corrected for the response of the detector system.

3. Cartesian coordinates

3.1. $Tb(hfa)_3(tppo)_2$

S0

Electronic energy = -3694.795016392650 (hartree)

Tb	-5.879256825298	-2.597253030223	-0.601662175228
P	-4.224266080549	-2.072677885012	2.680146712868
C	-2.756534757978	-1.346968438674	1.911623743856
C	-2.226288287076	-1.930738642875	0.752262453210
C	-1.088776140480	-1.392128659840	0.145742421551
C	-0.465315499559	-0.271752246702	0.700374603936
C	-0.982154105158	0.311750153309	1.860360483100
C	-2.124063328890	-0.222166851988	2.466140214533
C	-4.759298866612	-1.007015293732	4.034614424760
C	-4.213581731812	-1.142850200273	5.320342135098
C	-4.639606898300	-0.303953746864	6.355068628293
C	-5.608513416034	0.673834385697	6.111107153539
C	-6.150267899486	0.816177655930	4.830569555618
C	-5.725183457239	-0.020016293372	3.794395712166
C	-3.747586727597	-3.662270519979	3.389988295178
C	-2.420757530195	-3.915432221499	3.771168117251
C	-4.720539207160	-4.653477001879	3.573601395495
H	-2.693321978384	-2.799712460371	0.314458392385
H	-0.689649575924	-1.845044394676	-0.752636910280
H	-0.498939064965	1.179898084307	2.289492906049
H	-2.505837743021	0.246322023501	3.362408434842
H	-3.457604161902	-1.888941949011	5.523859124851
H	-4.217282888462	-0.410879179032	7.345834739771
H	-5.936830407142	1.322548755244	6.912941436045
H	-6.897367771764	1.575747465757	4.639862333822
H	-6.144977722583	0.104209464791	2.805281740712
H	-1.657055249318	-3.158660232404	3.649418351002
H	-5.747903844893	-4.466693353540	3.292416318351
C	-2.073978709786	-5.152258163494	4.324146258811
C	-3.047905175296	-6.139900712408	4.498678381373
C	-4.370980471807	-5.890054357951	4.123691777793
H	-1.050148143686	-5.345151184528	4.617114447852
H	-2.777634862551	-7.097479971752	4.924615582722
H	-5.125742291001	-6.653800861314	4.259239160669
O	-5.368992530484	-2.250451283862	1.679597460299
O	-4.061120898164	-2.913825619885	-2.108772316481
C	-3.416145182377	-3.923710581617	-2.460613947208
C	-3.260494812173	-5.129167984070	-1.761300133525
C	-3.836324855394	-5.292679445923	-0.498214313217
O	-4.607619478912	-4.510423452805	0.104901058289
H	-2.632278735962	-5.912986189028	-2.172032751333
C	-2.723569510710	-3.768028445062	-3.824584023621
F	-1.838166336435	-2.767377956714	-3.791446356352
F	-3.633216277282	-3.487197842824	-4.765500773136
F	-2.073345881341	-4.877983510361	-4.210050760873
C	-3.454538323248	-6.537737695906	0.318163209660
F	-2.910216686093	-7.508301457700	-0.429565291846
F	-2.550777270053	-6.194548934364	1.254244012408
F	-4.514910524377	-7.049918159745	0.944365438038
O	-4.737468588179	-0.537427309215	-0.442418453680
C	-4.936616087422	0.592092132694	-0.949112769194
C	-5.745760799407	0.919769559987	-2.035448633202
C	-6.493130082791	-0.053322047540	-2.716731581441
O	-6.593003811021	-1.273024248330	-2.469389476302
H	-5.793547218393	1.951441356937	-2.369723276456
C	-4.155711313288	1.714535504641	-0.249301265018
F	-2.842039337941	1.584028647615	-0.478480455133
F	-4.517504556571	2.940931003051	-0.643411453952
F	-4.342870392291	1.643221708150	1.079294244657
C	-7.357908187270	0.429390887458	-3.895103694610
F	-6.900288380364	1.562398846084	-4.447084956500
F	-7.424066665961	-0.494581925985	-4.856201619377
F	-8.606873536919	0.668675630124	-3.464233663596
O	-7.646333556539	-1.124837056343	0.088269012683
C	-8.416757918142	-1.135210596299	1.070217519114
C	-8.771826873859	-2.235412337688	1.864184958020
C	-8.346963268611	-3.518748330097	1.509198446630
O	-7.584310366677	-3.843442970820	0.568151083283

H	-9.450853782686	-2.102304832003	2.700487513437
C	-9.057420386238	0.230316973850	1.370094404119
F	-9.977846834724	0.518065908260	0.440890850982
F	-8.134151911189	1.195544181936	1.350044311408
F	-9.661047003990	0.267974198139	2.568913632750
C	-8.923859984992	-4.712640440267	2.288154155067
F	-9.473900231418	-4.354458159711	3.457526670454
F	-7.981871934642	-5.622011514938	2.541914561179
F	-9.883095929903	-5.309771196225	1.558127298083
P	-8.021623535337	-4.792948997605	-2.622805135464
O	-6.694469108705	-4.196748104812	-2.143070501209
H	0.416865420854	0.144588791677	0.231524342326
C	-9.301069365597	-3.518565406157	-2.714515542184
C	-9.463552144598	-2.767490867312	-3.888183058058
C	-10.100970927937	-3.230262644913	-1.598545503361
C	-10.428315323213	-1.757392877773	-3.949961493528
H	-8.841568718965	-2.957570443334	-4.753337001939
C	-11.053913719159	-2.209971109678	-1.657302583799
H	-9.993655273500	-3.793632907645	-0.684599055879
C	-11.219833268820	-1.475077274941	-2.833603574732
H	-10.552287317045	-1.183809883300	-4.859355414816
H	-11.665479903678	-1.991048839202	-0.791606181555
H	-11.959594631365	-0.686355905760	-2.879627024718
C	-7.835125668347	-5.529375966493	-4.259028019451
C	-6.555320918924	-5.718788385434	-4.799846465574
C	-8.962502394076	-5.928005792388	-4.992378177783
C	-6.405432971792	-6.293099193870	-6.065834537150
H	-5.674670179143	-5.425245380096	-4.243804371005
C	-8.809715071367	-6.501960897893	-6.258252148683
H	-9.956924757298	-5.788473337534	-4.589538516289
C	-7.531989153349	-6.684044420142	-6.795236555166
H	-5.416035958902	-6.435810725987	-6.480689410739
H	-9.681815315831	-6.805123983457	-6.822951607865
H	-7.414955393197	-7.128530209665	-7.775187693773
C	-8.584410320891	-6.135282204171	-1.555307059030
C	-9.920473284444	-6.562508676483	-1.583487278678
C	-7.668729941859	-6.786174432700	-0.716948244809
C	-10.338007830579	-7.616247308933	-0.764465184523
H	-10.638805101796	-6.080078846439	-2.233019723795
C	-8.088434210600	-7.838819186235	0.101389146206
H	-6.631778954855	-6.480121580455	-0.697034717632
C	-9.423168819379	-8.252787992556	0.079385024300
H	-11.370739209311	-7.939215168961	-0.783990449709
H	-7.378755230009	-8.333426828598	0.751861386928
H	-9.747821610716	-9.067334459556	0.713857826822

T1

Electronic energy = -3694.696355916378 (hartree)

Tb	-5.871043070829	-2.562365935828	-0.614496979733
P	-4.185869326776	-2.106409318763	2.660611643403
C	-2.719953631294	-1.385704151246	1.883557814640
C	-2.165618663128	-2.003947585758	0.753417737208
C	-1.040165770936	-1.459533021975	0.129775923222
C	-0.452948508143	-0.298268923979	0.637658909518
C	-0.992564013653	0.319034129554	1.769252832941
C	-2.123098768594	-0.220095727735	2.391415940995
C	-4.674640072646	-1.077765196002	4.060819827324
C	-4.010218123735	-1.178594826685	5.292688237704
C	-4.397151121968	-0.371054301738	6.366863985389
C	-5.446102987027	0.540615942149	6.216213784948
C	-6.107602354814	0.647696831965	4.989683204146
C	-5.722138468522	-0.157653031961	3.914300896097
C	-3.728613936295	-3.721103741975	3.325114653206
C	-2.403750436316	-4.002485944154	3.692901728635
C	-4.716042294543	-4.700725012132	3.494838464316
H	-2.605297141119	-2.903634864965	0.350557765681
H	-0.622726066005	-1.939149112617	-0.746141384581
H	-0.537882886113	1.219370893232	2.161709200538
H	-2.527397547944	0.280244061703	3.260066058068
H	-3.191051746295	-1.872745158644	5.422203888483
H	-3.882354080662	-0.450698985571	7.315556162233
H	-5.743997108650	1.165474861961	7.048296519777
H	-6.917137974135	1.356233693681	4.870993305832
H	-6.236775072715	-0.059787822424	2.968554659635

H	-1.629277173020	-3.255013158004	3.581504386451
H	-5.742188410764	-4.493440233887	3.222563331671
C	-2.073331077907	-5.254718994074	4.220570238667
C	-3.061781989171	-6.229926359471	4.382853115045
C	-4.382790661725	-5.952575767843	4.020100923659
H	-1.051036294492	-5.469030015950	4.503820709067
H	-2.804274129694	-7.199358935491	4.789426665045
H	-5.148559059028	-6.707011031661	4.145660652623
O	-5.349179990947	-2.237326014942	1.672969877563
O	-4.068576554083	-2.884093295549	-2.135356320888
C	-3.438556062389	-3.897904619355	-2.499743777549
C	-3.295248607310	-5.112487278847	-1.812376147755
C	-3.863231402130	-5.281694125343	-0.547323527771
O	-4.619886594718	-4.496204883399	0.071882026427
H	-2.679622560141	-5.900179255623	-2.234565159102
C	-2.749713113235	-3.739334403396	-3.865568618803
F	-1.848842414606	-2.753050372972	-3.826721822865
F	-3.658978835918	-3.435791087365	-4.799575887361
F	-2.118412320499	-4.855635510368	-4.264508690663
C	-3.484443562901	-6.540037731194	0.250065370063
F	-2.979915165428	-7.514927186099	-0.519736578240
F	-2.548525038839	-6.221825813680	1.162809690602
F	-4.536358082357	-7.037657729475	0.901851136492
O	-4.636652826340	-0.548089034336	-0.478435201711
C	-4.989873257763	0.644608602904	-0.788754977557
C	-5.968442451382	0.955019506375	-1.781496629557
C	-6.700193430039	0.026775698453	-2.581630695638
O	-6.660779725721	-1.246741829731	-2.448481640978
H	-6.173823402675	2.012937754866	-1.951789756982
C	-4.349050425424	1.746134653122	0.009086344154
F	-3.023187503925	1.832306044428	-0.203321195712
F	-4.875957270182	2.953028811727	-0.264182980770
F	-4.502803582069	1.533001978465	1.335426292064
C	-7.714214037702	0.570424440538	-3.555644415673
F	-7.465024386922	1.854755723992	-3.883872775150
F	-7.743859455026	-0.141991455262	-4.689178431701
F	-8.962808268729	0.540945564322	-3.042542182450
O	-7.501293279712	-0.936476121909	0.128263675383
C	-8.316817874951	-0.920415407301	1.070252487278
C	-8.779553054703	-2.014018921838	1.818773051817
C	-8.429083745267	-3.313746128795	1.451022707039
O	-7.670997097924	-3.674630022130	0.516910228194
H	-9.474512713563	-1.852201018320	2.636756090692
C	-8.887841410655	0.475188538241	1.374134049445
F	-9.817469039864	0.793874363054	0.463853765819
F	-7.925090148036	1.400387553127	1.319913342855
F	-9.458608033806	0.547761486256	2.586231232251
C	-9.073914770512	-4.482511110190	2.213393889904
F	-9.700894519350	-4.095463256816	3.333301637050
F	-8.155044900607	-5.389309806135	2.554323893322
F	-9.982510555521	-5.093335107579	1.433684294576
P	-7.983850263289	-4.809111902858	-2.648433342593
O	-6.686705452686	-4.173801298894	-2.139731138469
H	0.419655209529	0.122814734255	0.155282332133
C	-9.268916717865	-3.558435684873	-2.881089682463
C	-9.416308685046	-2.917648471903	-4.120765787926
C	-10.101741955606	-3.189660996317	-1.813832691268
C	-10.408862004795	-1.948959979421	-4.298759360161
H	-8.765083991151	-3.164161709261	-4.949288064689
C	-11.083913449659	-2.211468867414	-1.990192431954
H	-10.000578102148	-3.662205048602	-0.848681694986
C	-11.242933703881	-1.596414486382	-3.234363386430
H	-10.523476299508	-1.462982820694	-5.258963798387
H	-11.724836638243	-1.933791166972	-1.163488433862
H	-12.005859414242	-0.841055719631	-3.371470326857
C	-7.708399668974	-5.644493993790	-4.224056296582
C	-6.402381697619	-5.831758300296	-4.699175203748
C	-8.792719278225	-6.125215630021	-4.973114112712
C	-6.184005156992	-6.484186463146	-5.916330661332
H	-5.553757491399	-5.477113181985	-4.129677434474
C	-8.571479888059	-6.777295720771	-6.190109290359
H	-9.806559898426	-5.989622253267	-4.620544971306
C	-7.267920886137	-6.956185174896	-6.662129720362
H	-5.174521158434	-6.624780666541	-6.280424193232

H	-9.410567068472	-7.143632726800	-6.767240137640
H	-7.097849077205	-7.461220153611	-7.604255366189
C	-8.592947226680	-6.089879994134	-1.531795527431
C	-9.928500571925	-6.516237767627	-1.588369952993
C	-7.712240524732	-6.697142307439	-0.626371706804
C	-10.379923092838	-7.526138096429	-0.732812364242
H	-10.620226489130	-6.067933574801	-2.289202121759
C	-8.165554675401	-7.705987304184	0.228357638563
H	-6.676450986680	-6.391194835105	-0.583237591661
C	-9.499610268076	-8.119466166587	0.176722739078
H	-11.412043567589	-7.848874434089	-0.775205855834
H	-7.482349380903	-8.167146031148	0.929786758281
H	-9.850386211378	-8.900233559929	0.839140397594

EET

Electronic energy = -3694.695945786131 (hartree)

Tb	-5.887106171695	-2.557787827177	-0.599814690576
P	-4.191714173121	-2.105695516282	2.669702663394
C	-2.729425426097	-1.382915435267	1.887656782422
C	-2.178534508716	-1.999603940970	0.755031399348
C	-1.055090680181	-1.454248679379	0.128629179786
C	-0.466234595616	-0.293731290735	0.636328724113
C	-1.002641335258	0.322110859269	1.770259506685
C	-2.131338922625	-0.217758878661	2.395068706623
C	-4.677479934004	-1.074747021992	4.069137919337
C	-4.023680031888	-1.186157665945	5.305789561520
C	-4.408635788858	-0.375881290857	6.378613333222
C	-5.444658270863	0.549430014358	6.221663177810
C	-6.094849364685	0.667806506349	4.990087720242
C	-5.711148857234	-0.140212503821	3.916056715496
C	-3.729907137326	-3.719509635538	3.333191431055
C	-2.403284830317	-3.998893622388	3.696127642502
C	-4.715015417905	-4.701073053130	3.505236919562
H	-2.618710700944	-2.899203660252	0.352919636984
H	-0.640193979562	-1.932793714370	-0.749093755389
H	-0.546748539745	1.221898572564	2.162564579321
H	-2.532761186320	0.281064709206	3.265926463050
H	-3.214571639381	-1.891033545078	5.440469683243
H	-3.902232960890	-0.463805640612	7.331091719485
H	-5.741107800989	1.176361827612	7.052713336872
H	-6.894274085641	1.386905262682	4.866595609242
H	-6.216099036051	-0.033413003236	2.965886336869
H	-1.630481159276	-3.249976473990	3.582854296677
H	-5.742472308021	-4.495471755039	3.236458318746
C	-2.068877324479	-5.251003759454	4.221547108723
C	-3.055018721592	-6.228189413997	4.386201383455
C	-4.377715058736	-5.952846263022	4.028034655644
H	-1.045260088596	-5.463850305889	4.501126348339
H	-2.794362814084	-7.197591517706	4.790820046829
H	-5.141661604889	-6.708819664321	4.155460094617
O	-5.358482803899	-2.238531755952	1.686679632304
O	-4.078809234717	-2.869336209660	-2.118018277584
C	-3.449533708444	-3.881760419183	-2.488031938448
C	-3.305984379125	-5.099649915414	-1.806919812765
C	-3.873241528921	-5.274670742624	-0.542056946366
O	-4.629782854202	-4.492284615905	0.080735802986
H	-2.690837674601	-5.885439377284	-2.233359896075
C	-2.762178784576	-3.716768639629	-3.853823765395
F	-1.860163360109	-2.731557967822	-3.811039441792
F	-3.672275199527	-3.407183269974	-4.785164989798
F	-2.132621065498	-4.831582466059	-4.259604557807
C	-3.493186113089	-6.536114354174	0.249938494362
F	-2.991769521992	-7.508724461746	-0.524866261385
F	-2.554146963373	-6.221950496471	1.160980541254
F	-4.543430716502	-7.035063301465	0.903257260163
O	-4.666869187787	-0.536425145436	-0.458580864478
C	-4.987069268895	0.645159488214	-0.817154067188
C	-5.933338869960	0.961513585663	-1.828421480848
C	-6.683845078085	0.023984865420	-2.588645688481
O	-6.672201801910	-1.240846364511	-2.433291190508
H	-6.097829371797	2.017354056887	-2.042232101114
C	-4.304476517849	1.755947924893	-0.054237661733
F	-2.980293754172	1.788227079093	-0.282555167670
F	-4.792987180776	2.969905378376	-0.358540009821

F	-4.458883108869	1.582185325240	1.275390614847
C	-7.677780604265	0.558174909015	-3.598051745244
F	-7.405733352909	1.825753875338	-3.963343138904
F	-7.701867637280	-0.189388760410	-4.708122161143
F	-8.928255885721	0.559332042025	-3.094250937599
O	-7.531467042163	-0.954774044023	0.147790399996
C	-8.341992077482	-0.947520928991	1.094095234819
C	-8.791974081760	-2.047345108999	1.841473356056
C	-8.432174146237	-3.342647561124	1.467465806030
O	-7.673149811751	-3.693915961784	0.530391187606
H	-9.485709066359	-1.894549814883	2.662209580537
C	-8.921954657088	0.442615073515	1.406204661967
F	-9.845007315031	0.766190481412	0.490969329833
F	-7.963030588266	1.372200974547	1.370611534269
F	-9.504257443093	0.500833554279	2.613970665489
C	-9.068708590291	-4.518994118257	2.224762616078
F	-9.688879741410	-4.142452071196	3.352092905839
F	-8.146118049568	-5.426930675629	2.551860443836
F	-9.981755194881	-5.124596273562	1.445910985833
P	-7.986236319591	-4.804717597066	-2.644805008167
O	-6.690172765564	-4.170090425346	-2.132601110225
H	0.404903018446	0.127962999289	0.151879338889
C	-9.271127948064	-3.553263598789	-2.873256833697
C	-9.419695680350	-2.909705625293	-4.111335834002
C	-10.100076949262	-3.184104584670	-1.803087212612
C	-10.409123589468	-1.936997490641	-4.284667487608
H	-8.771014948346	-3.156214819795	-4.941840234022
C	-11.079118075138	-2.201973909830	-1.974946913032
H	-9.997848343686	-3.658958172584	-0.839079214956
C	-11.238816545315	-1.583324088539	-3.217243791770
H	-10.524378480536	-1.448666759372	-5.243610320786
H	-11.716791893181	-1.923587721729	-1.145951776532
H	-11.999185233598	-0.824771754779	-3.350843283070
C	-7.709211152495	-5.635196527354	-4.222706384254
C	-6.402693066991	-5.822152296120	-4.696581954293
C	-8.792800024570	-6.114729459499	-4.973535015400
C	-6.183162680497	-6.473444975233	-5.914138034347
H	-5.554603989323	-5.468901454974	-4.125428864949
C	-8.570386185265	-6.765572082197	-6.190968801008
H	-9.806960196010	-5.979928058952	-4.621495632417
C	-7.266321946032	-6.944572096674	-6.661596983611
H	-5.173271535727	-6.614340491232	-6.277015601100
H	-9.408862895685	-7.131454269077	-6.769280066764
H	-7.095291438175	-7.448931914455	-7.603897373700
C	-8.595349403189	-6.090439411420	-1.534027416892
C	-9.931986102422	-6.513434844182	-1.589580436205
C	-7.713066052705	-6.706405602227	-0.636099240025
C	-10.382885422059	-7.528737662601	-0.740188585752
H	-10.624786301102	-6.058942324536	-2.285373341027
C	-8.165839455765	-7.720709391330	0.212425314591
H	-6.676336630475	-6.403394548487	-0.594636416371
C	-9.500966497409	-8.130924328367	0.161971218651
H	-11.415734532516	-7.849218776620	-0.781772935835
H	-7.481271221816	-8.188650975822	0.908016710299
H	-9.851158902687	-8.915917734086	0.819667701704

ISC

Electronic energy = -3694.678852992704 (hartree)

Tb	-5.802614062876	-2.551239401981	-0.581258551295
P	-4.253962172463	-1.985489920243	2.693725909128
C	-2.772673750143	-1.320658323738	1.895781243296
C	-1.990796805190	-2.149737057998	1.076557074046
C	-0.875303508406	-1.635512125222	0.410262886332
C	-0.530029970334	-0.290190472197	0.559936645967
C	-1.300228140953	0.541182612268	1.377223740631
C	-2.418718725343	0.030090895429	2.042768691802
C	-4.712433368255	-0.918107788109	4.074125372947
C	-3.786092917308	-0.631513112071	5.087810907011
C	-4.148009726007	0.193524856751	6.157194683194
C	-5.436426710824	0.731872787270	6.221755862090
C	-6.364835481918	0.444242753555	5.217262599668
C	-6.005745108403	-0.380498273219	4.146999185496
C	-3.830542388556	-3.577724387611	3.430677214248
C	-2.550422288449	-3.812286279340	3.955924541355

C	-4.800418478196	-4.585365295482	3.501449938531
H	-2.243412087819	-3.192247230026	0.950468918125
H	-0.278935787356	-2.280145251832	-0.222343738062
H	-1.033885928533	1.584008110155	1.491184287391
H	-3.009643053962	0.694228380580	2.658473901265
H	-2.784317482711	-1.037916562132	5.046440764866
H	-3.429339608067	0.415902905635	6.935279568409
H	-5.715251817643	1.370594877311	7.049927266596
H	-7.362938024090	0.859741822289	5.267833620425
H	-6.735683787032	-0.597162123928	3.378395717346
H	-1.790270208884	-3.043072082608	3.919340670299
H	-5.792836438359	-4.410486954500	3.112935296173
C	-2.244192103987	-5.049252205872	4.532204579208
C	-3.214199505357	-6.054207985536	4.591272714339
C	-4.492744677942	-5.821413245802	4.077068512093
H	-1.254887252505	-5.228518879348	4.932702963530
H	-2.975791462902	-7.011454894237	5.036550202583
H	-5.245654727967	-6.597507514033	4.124443347223
O	-5.420748401197	-2.126022713819	1.706582723109
O	-4.210209824735	-3.006916176637	-2.331603818520
C	-3.511737180438	-3.994642480677	-2.617281704051
C	-3.239513972997	-5.116966997207	-1.812335336978
C	-3.719644913750	-5.184487930380	-0.507324391167
O	-4.443289776587	-4.352163409364	0.099182089854
H	-2.604987281246	-5.910882115109	-2.192883742209
C	-2.910793989769	-3.936872427645	-4.032987160061
F	-2.295768662502	-2.769905357523	-4.236827561307
F	-3.889860546761	-4.051661145616	-4.942398512049
F	-2.025165524501	-4.916949885783	-4.268481143229
C	-3.312149052018	-6.381195107190	0.364735702828
F	-2.671034081561	-7.338970354749	-0.319378574045
F	-2.493062563842	-5.970337410784	1.347921617173
F	-4.385705600949	-6.936419250888	0.934139175230
O	-4.273247349019	-0.748586174290	-0.587930247782
C	-5.091393576263	0.270716604669	-0.836451936516
C	-5.032483192520	0.485207903413	-2.302478367918
C	-5.813927094807	-0.367297509701	-3.138513086391
O	-6.457478765675	-1.331800390866	-2.708948216032
H	-4.281356008485	1.137385186560	-2.760358837152
C	-5.117298709934	1.441463335245	0.075374642158
F	-3.972519021449	2.177467167827	0.071587781105
F	-6.107165179730	2.286418238061	-0.263106143913
F	-5.308158233310	1.066694179837	1.356684469343
C	-5.866995060386	-0.135776316496	-4.654514036520
F	-5.240171599225	0.993701111270	-5.010998501776
F	-5.289560465077	-1.152153922487	-5.298118404425
F	-7.136946694267	-0.053180625407	-5.060427577600
O	-7.760632540213	-1.268409220950	-0.141451008704
C	-8.579550530546	-1.252241872829	0.806344667489
C	-8.903896681143	-2.304591784466	1.668856301197
C	-8.328955446921	-3.568653765833	1.479973296882
O	-7.453951318347	-3.889966609194	0.647464391392
H	-9.659321395883	-2.160152922035	2.435300691478
C	-9.267875270781	0.105609789238	1.023477264227
F	-9.552280816196	0.694397341204	-0.137722109515
F	-8.450342608414	0.914580535111	1.712098848561
F	-10.410093348687	-0.002736980572	1.723263956063
C	-8.882114980193	-4.734769281499	2.322412144140
F	-9.397260953587	-4.322261247651	3.490109487974
F	-7.942777762213	-5.640672213295	2.585014405549
F	-9.867442702528	-5.356902280683	1.646502607639
P	-8.093723912202	-4.711681755009	-2.487340176317
O	-6.722052886356	-4.171945863580	-2.069644955377
H	0.334205611345	0.107627514121	0.043880137349
C	-9.348225545963	-3.413473149576	-2.376111738667
C	-9.347473676320	-2.374684886603	-3.318637829309
C	-10.285946759805	-3.393119935071	-1.332463390901
C	-10.269193404119	-1.328753748819	-3.217125226949
H	-8.628925281912	-2.372389976351	-4.128577780613
C	-11.207545914549	-2.346807231319	-1.233150793025
H	-10.301992921590	-4.173451087632	-0.587875209522
C	-11.198836992380	-1.314351754204	-2.174202209474
H	-10.260981126413	-0.528651000777	-3.945867533960
H	-11.924593351476	-2.333337497129	-0.422577186929

H	-11.910549808691	-0.502792868781	-2.094330107019
C	-8.052180706109	-5.294374619413	-4.193928850678
C	-6.837697565640	-5.711471594921	-4.755616893334
C	-9.226328720570	-5.354845169920	-4.959762096354
C	-6.794166022822	-6.168774479287	-6.076265557509
H	-5.927391905521	-5.686838070927	-4.170930117329
C	-9.180195414748	-5.813223021128	-6.279976114950
H	-10.174599224838	-5.047200203908	-4.538600737095
C	-7.964605075068	-6.218174134347	-6.838869187682
H	-5.853640383418	-6.486699058687	-6.507079748321
H	-10.087104172237	-5.855289840654	-6.869194122206
H	-7.930238270157	-6.572958961714	-7.860871786578
C	-8.589755938702	-6.144810097548	-1.508501778310
C	-9.870771606849	-6.697559751362	-1.653561541383
C	-7.675627359022	-6.740986364954	-0.627962084877
C	-10.242891577719	-7.814901352776	-0.899723978534
H	-10.582323616479	-6.259634375260	-2.341296126577
C	-8.049452983466	-7.858742652701	0.123572583405
H	-6.676077958140	-6.341627510772	-0.520592822030
C	-9.333919391312	-8.393447065552	-0.009119247177
H	-11.235489724548	-8.232673454805	-1.006717112864
H	-7.343294725425	-8.309619981168	0.808789325872
H	-9.623282628642	-9.257789441355	0.574539333504

3.2. $Eu(hfa)_3(tppo)_2$

S0

Electronic energy = -3693.607914004479 (hartree)

Eu	-5.845969338057	-2.624662906134	-0.650015551256
P	-4.230879028801	-2.076799334903	2.683498168486
C	-2.753521987379	-1.355273225915	1.930442569498
C	-2.242823474618	-1.908710667716	0.747803653196
C	-1.095385313746	-1.375725925162	0.155224226126
C	-0.442721299431	-0.291518817329	0.747045682612
C	-0.940304846965	0.261395354120	1.930287842286
C	-2.091427945769	-0.267622957159	2.522833091443
C	-4.800719323259	-0.987848088490	4.004843998851
C	-4.302894681048	-1.112104414284	5.311065649476
C	-4.758240538037	-0.256423358685	6.319234156638
C	-5.709386444583	0.726008649737	6.028551637706
C	-6.204152633456	0.856049206336	4.727866820573
C	-5.749505322557	0.003255226372	3.718147404567
C	-3.753984303523	-3.649560554525	3.429574216723
C	-2.434517425225	-3.880885691395	3.848375875409
C	-4.718543402425	-4.651218278384	3.599355062008
H	-2.735591212600	-2.746650782593	0.278523842681
H	-0.712041125654	-1.804208432883	-0.761851103256
H	-0.434087744204	1.101043850271	2.388598272075
H	-2.456616378083	0.174081907361	3.439431653070
H	-3.561797288636	-1.862113283531	5.551338764433
H	-4.372674736684	-0.354220470835	7.325794174738
H	-6.060627822425	1.387511099333	6.809958380245
H	-6.938026367887	1.618608695193	4.501191175711
H	-6.133937722190	0.117079818810	2.713429955791
H	-1.677374979955	-3.116060979339	3.736767767824
H	-5.740125933272	-4.481059645624	3.288775790815
C	-2.086750910684	-5.106467413734	4.425345048047
C	-3.052206693260	-6.104751517266	4.585921791202
C	-4.367930753345	-5.876702841740	4.173085530873
H	-1.068657813313	-5.282496905103	4.747453579893
H	-2.781134341623	-7.053620337374	5.030448223513
H	-5.116238399106	-6.648581525764	4.297915942604
O	-5.354472729780	-2.283116684287	1.664306279364
O	-4.012927069460	-2.973107627839	-2.170393848627
C	-3.310042567162	-3.959071395046	-2.475195090894
C	-3.115379984077	-5.136543536741	-1.738037444685
C	-3.733762165341	-5.308698213998	-0.496386586239
O	-4.557697804244	-4.551692429198	0.068086452135
H	-2.435925781833	-5.896991934633	-2.109430842503
C	-2.611994994800	-3.820501382092	-3.838561266793
F	-1.951805195556	-2.664179167462	-3.920883007670
F	-3.533653952893	-3.843186045722	-4.812709690101
F	-1.740341529509	-4.811217466229	-4.085813159438
C	-3.342191554830	-6.531842652567	0.348086261795

F	-2.711450088136	-7.480022640815	-0.358760686952
F	-2.511280620125	-6.142820082933	1.332977382796
F	-4.413370344559	-7.088684249823	0.915570998858
O	-4.699270614112	-0.542101106212	-0.466829198956
C	-4.888778357076	0.595229865130	-0.961048055687
C	-5.693837248016	0.941128596610	-2.043968271242
C	-6.441930951989	-0.020059122521	-2.743284675525
O	-6.537860784019	-1.244188077617	-2.520900414659
H	-5.739067610043	1.978259673565	-2.361444902890
C	-4.099627327299	1.703316669996	-0.247396566540
F	-2.786806802580	1.564308425746	-0.476535131290
F	-4.450600332641	2.937321950315	-0.626880918065
F	-4.288117398883	1.618158404857	1.080618593915
C	-7.313170971229	0.488856254582	-3.906476163632
F	-6.837817948974	1.614961346823	-4.457952695463
F	-7.415921377439	-0.426581290085	-4.871910265888
F	-8.549350524079	0.754056978128	-3.454051094928
O	-7.654068229349	-1.150763126014	0.032989603081
C	-8.428215648662	-1.162191391718	1.011912844765
C	-8.777117921158	-2.260191665452	1.812473622321
C	-8.343364668013	-3.544123842187	1.469970339874
O	-7.583310753503	-3.873686519512	0.528405591969
H	-9.456541477515	-2.124671801921	2.648095706652
C	-9.080512568500	0.200187177907	1.303014763924
F	-9.996509542468	0.478549998567	0.366608142923
F	-8.163716972921	1.171836226968	1.285788606550
F	-9.692466750566	0.238387665000	2.497589835753
C	-8.903078837862	-4.733317866933	2.269789907566
F	-9.448306879745	-4.364200429760	3.438021845005
F	-7.949333842053	-5.628752533881	2.529753520257
F	-9.860738541223	-5.352586840018	1.556434463953
P	-8.052098682805	-4.809311331932	-2.644748996136
O	-6.703020224781	-4.222462808458	-2.214225129878
H	0.446958822846	0.120692449345	0.288853852100
C	-9.325617303975	-3.525997081605	-2.668831602481
C	-9.462835470154	-2.699320194964	-3.793622197986
C	-10.140536851511	-3.302441229224	-1.548870720369
C	-10.414826240061	-1.675440003273	-3.803528698895
H	-8.830715544751	-2.843131973854	-4.660562012201
C	-11.080775144839	-2.268697765208	-1.554855675067
H	-10.052822771613	-3.923434266310	-0.671177580667
C	-11.219563840521	-1.456100079434	-2.682558202495
H	-10.519624587868	-1.043747459479	-4.676093640250
H	-11.702756853305	-2.098729436996	-0.685577634167
H	-11.949368580997	-0.656833663206	-2.687671107008
C	-7.939258095623	-5.519996755143	-4.298969852636
C	-6.682365560505	-5.771926432969	-4.866815153649
C	-9.100566979749	-5.837083433124	-5.019169133366
C	-6.588034543292	-6.327604490558	-6.146483634316
H	-5.777100194319	-5.541007685209	-4.320563735211
C	-9.003368146419	-6.392647372390	-6.298683781081
H	-10.078232878924	-5.649184709294	-4.595396261078
C	-7.747885196216	-6.637391237313	-6.862581570553
H	-5.615984374939	-6.518933999439	-6.582211862275
H	-9.901275206185	-6.633132558897	-6.853011087104
H	-7.673889589260	-7.067661759421	-7.853013336808
C	-8.573385459925	-6.166519750093	-1.575049517027
C	-9.897014463277	-6.630646541855	-1.600735529417
C	-7.638451169229	-6.791149051900	-0.737401198812
C	-10.284405501180	-7.693635518180	-0.778929879481
H	-10.629248190119	-6.168133770593	-2.249179153437
C	-8.028194430143	-7.853261241183	0.083554636280
H	-6.609865612159	-6.457053897123	-0.718510165029
C	-9.351251454538	-8.303305433393	0.064668816504
H	-11.308038668374	-8.044487698207	-0.795991732439
H	-7.304379455281	-8.327121641783	0.733895622481
H	-9.652643940169	-9.125050040064	0.701328494791

T1

Electronic energy = -3693.508817779012 (hartree)

Eu	-5.856137045767	-2.561387253793	-0.632886060205
P	-4.180953403021	-2.104082362940	2.672597233203
C	-2.718116221262	-1.379156565201	1.894065673347
C	-2.173105579036	-1.987081595715	0.753800487912

C	-1.050468282657	-1.439045039317	0.128248710404
C	-0.457027688562	-0.284413602271	0.643999454279
C	-0.987678520633	0.322711837095	1.785315418616
C	-2.115222380279	-0.220130117285	2.409671169543
C	-4.674167193797	-1.076421888331	4.071900478817
C	-4.007787388218	-1.171588035543	5.303149009032
C	-4.398415976732	-0.364971658514	6.376693378600
C	-5.453342392214	0.539808138048	6.226137880753
C	-6.117328034259	0.640701513059	5.000429584794
C	-5.728222087430	-0.163728666563	3.925668031350
C	-3.717662969172	-3.716954799290	3.336967192752
C	-2.392341385381	-3.991995963521	3.707733693758
C	-4.700233454089	-4.702412645254	3.501414928702
H	-2.619057145578	-2.880424388041	0.343321205842
H	-0.640386147586	-1.910385984522	-0.755611414568
H	-0.528199158313	1.217928990458	2.183830197712
H	-2.512493269941	0.272214619047	3.286062827884
H	-3.184141323820	-1.860459064350	5.432534601733
H	-3.881895484424	-0.440021218887	7.324818098541
H	-5.754104433562	1.163954186089	7.057723159213
H	-6.931771269230	1.343622953068	4.881917889089
H	-6.245507539999	-0.071045063992	2.980766439001
H	-1.621566748289	-3.240212976569	3.599517111556
H	-5.726677979450	-4.500519798655	3.225938606401
C	-2.056658534447	-5.243421287766	4.233968116973
C	-3.040282149826	-6.224278970532	4.391588250945
C	-4.361669842155	-5.953458885552	4.025238186587
H	-1.034028855461	-5.452781875675	4.519693456494
H	-2.778693737610	-7.193072631903	4.797069746586
H	-5.123600878599	-6.712398165466	4.146922416796
O	-5.342723163064	-2.239613277838	1.683230936582
O	-4.034487274236	-2.917273514671	-2.165119292923
C	-3.407172523214	-3.937003809510	-2.517525288827
C	-3.264319949627	-5.143763864162	-1.815955894102
C	-3.833453085593	-5.303988042553	-0.550062463554
O	-4.591190541150	-4.515483625700	0.063945328223
H	-2.648788630231	-5.935464568288	-2.230732230234
C	-2.723973830843	-3.798735064500	-3.888739629426
F	-1.843615000226	-2.793921223797	-3.877879355022
F	-3.642767730743	-3.537125376653	-4.826945439757
F	-2.071692274846	-4.911512908885	-4.262681030899
C	-3.457337040091	-6.558482769559	0.255074372808
F	-2.940537429944	-7.534282857548	-0.505161709527
F	-2.533138638150	-6.233176895587	1.177113602250
F	-4.514854942232	-7.058045105165	0.896951335746
O	-4.615439779707	-0.522896272972	-0.494996874785
C	-4.985699054021	0.671067948942	-0.780217688811
C	-5.974068326969	0.988407777862	-1.762200520252
C	-6.698718774266	0.067974629741	-2.578175483896
O	-6.643096416723	-1.207356020009	-2.472524631139
H	-6.194388623465	2.046853425526	-1.909286049951
C	-4.358285199668	1.764695677485	0.039069389392
F	-3.032537114669	1.865955009546	-0.166842117832
F	-4.895228636751	2.971286338812	-0.214682294620
F	-4.514207022129	1.527473349615	1.361533061780
C	-7.725346852932	0.619403913419	-3.535036906105
F	-7.493708619349	1.913045303542	-3.838444247026
F	-7.753711984472	-0.070365019627	-4.682354410226
F	-8.970242600348	0.564142904781	-3.014808970541
O	-7.502308328325	-0.911186623086	0.122366466522
C	-8.323001768745	-0.896712451340	1.059384109264
C	-8.797747739320	-1.992189611019	1.798882776087
C	-8.454752454910	-3.293476008808	1.430443670155
O	-7.699137185768	-3.659681163627	0.495857952849
H	-9.493569308269	-1.828481676790	2.615771097700
C	-8.885867933695	0.500754320919	1.372101079073
F	-9.811845705393	0.831525135956	0.462372051674
F	-7.917018188780	1.420190334934	1.325615664057
F	-9.458145610664	0.569227466022	2.583660876045
C	-9.098349797907	-4.459241062875	2.198871032188
F	-9.748269048484	-4.065764748640	3.303194122081
F	-8.171519376084	-5.347883536451	2.567688624762
F	-9.985184840419	-5.094917661419	1.414532841843
P	-7.990193230094	-4.826065609563	-2.667109447188

O	-6.687414888513	-4.189504763660	-2.173412244602
H	0.413322715959	0.139526460688	0.160059091267
C	-9.273715187007	-3.572610951403	-2.891760000154
C	-9.402174567970	-2.907028617847	-4.120331527297
C	-10.120116048356	-3.222575979695	-1.828880766988
C	-10.389386235361	-1.931967441818	-4.292779876405
H	-8.739992101631	-3.139710381466	-4.944278931930
C	-11.096881640345	-2.237894693873	-1.999306182505
H	-10.032609364591	-3.713019440265	-0.871387568171
C	-11.236864857424	-1.597742485340	-3.233081524667
H	-10.489474278864	-1.426909343230	-5.244724641412
H	-11.748050602824	-1.974274493354	-1.176006903906
H	-11.995583152504	-0.837346029463	-3.365621108975
C	-7.731180253927	-5.664746747211	-4.243590982533
C	-6.428449505024	-5.886909826500	-4.712639536577
C	-8.824648384502	-6.113371173586	-4.999204595223
C	-6.222143119957	-6.541888110630	-5.930569333803
H	-5.573190917354	-5.557267760417	-4.137701856582
C	-8.615427974200	-6.768118337556	-6.216883618160
H	-9.836138027821	-5.950896910629	-4.651238515313
C	-7.314982544209	-6.981736964445	-6.682999852350
H	-5.215147843642	-6.709403581791	-6.290107644060
H	-9.461444224214	-7.109618926808	-6.799107688188
H	-7.154254677756	-7.488789662100	-7.625670905383
C	-8.589359430740	-6.103228257227	-1.541229844568
C	-9.918072530922	-6.549243921595	-1.604475010561
C	-7.708201805571	-6.686820050219	-0.620589866933
C	-10.362814226153	-7.555260994450	-0.740888065016
H	-10.609794356050	-6.118347071822	-2.316128954134
C	-8.154897788534	-7.691858879200	0.242089578009
H	-6.677334627957	-6.365282252502	-0.570996155212
C	-9.482379066322	-8.125141417226	0.183423952730
H	-11.389863472899	-7.893078033785	-0.788435577444
H	-7.471696712030	-8.134702617605	0.955227473851
H	-9.828029399913	-8.902911549816	0.852035293285

EET
Electronic energy = -3693.507025977779 (hartree)

Eu	-5.816474268374	-2.552564477513	-0.676614781144
P	-4.163222325147	-2.099310347114	2.646244402971
C	-2.688599068125	-1.384490467891	1.881245568484
C	-2.134140331588	-2.001325031326	0.750249835533
C	-0.999847154197	-1.464302197785	0.136321708114
C	-0.405103035392	-0.311025807994	0.653653000344
C	-0.946399164899	0.306021734043	1.784576442947
C	-2.085354295889	-0.225935860530	2.397464277541
C	-4.658484957045	-1.082935772108	4.053604328892
C	-3.948170561990	-1.140589297671	5.262432923185
C	-4.341523479853	-0.349592296309	6.346467340748
C	-5.444684161934	0.500925162725	6.229725233906
C	-6.154732682825	0.562562508774	5.027491069515
C	-5.762911164677	-0.226322232572	3.942214290358
C	-3.716492430075	-3.717415395777	3.309514394657
C	-2.396179578541	-4.002352006907	3.690672511073
C	-4.708394637987	-4.693899026800	3.470569103410
H	-2.581526975697	-2.893844337635	0.338578682934
H	-0.581651612632	-1.943336891743	-0.739548111761
H	-0.486308725291	1.200646792062	2.183741667461
H	-2.491228888187	0.274802731605	3.264973928824
H	-3.088026557412	-1.788279538399	5.365114495127
H	-3.790114926104	-0.395076257306	7.276646172402
H	-5.747760501241	1.112901846526	7.069475243397
H	-7.007599089150	1.222604966848	4.935599260428
H	-6.318571143710	-0.164629671266	3.017115678516
H	-1.618311761984	-3.257420939780	3.586061756349
H	-5.731193870256	-4.483996047890	3.188078551973
C	-2.074709136230	-5.254559398435	4.223792513771
C	-3.067724446635	-6.226379567744	4.378217155111
C	-4.384185687250	-5.945857358801	4.001432940073
H	-1.055971202082	-5.471280742000	4.517815787876
H	-2.817233397160	-7.195526753249	4.789843956495
H	-5.153301203678	-6.697807835200	4.121097506446
O	-5.316162497060	-2.221288042085	1.644587993377
O	-4.000437110501	-2.944873990031	-2.201378439804

C	-3.376319805490	-3.970766188525	-2.540463072215
C	-3.246099679365	-5.172152056934	-1.827120287397
C	-3.822208668706	-5.315980084831	-0.562848477089
O	-4.576138156894	-4.515026251398	0.040437687970
H	-2.633914735815	-5.971684843528	-2.231571642341
C	-2.680770780262	-3.848627005448	-3.907060442670
F	-1.795339286266	-2.848551212760	-3.897499027394
F	-3.589740473381	-3.591532252426	-4.855678903357
F	-2.030843682367	-4.968427724219	-4.264209533120
C	-3.459625510538	-6.566563860499	0.254400556961
F	-2.949314678998	-7.553557320841	-0.495549941060
F	-2.535471338735	-6.241307523597	1.176338881075
F	-4.523498429702	-7.051335754452	0.897302257312
O	-4.541183283387	-0.529052400852	-0.565850025770
C	-4.977965678233	0.668519783440	-0.746988385422
C	-6.085691680205	0.966598191852	-1.615234829915
C	-6.742208536455	0.083875058801	-2.544814862674
O	-6.622841151751	-1.200309803302	-2.499789728484
H	-6.474922222499	1.986508244939	-1.564027790448
C	-4.420562848891	1.725555376560	0.155538083801
F	-3.094477285557	1.885409069324	-0.003379831021
F	-4.996631297754	2.925674210853	-0.041680814255
F	-4.597227433935	1.412109679855	1.464232666424
C	-7.871477656233	0.639812200630	-3.358535335368
F	-7.731115538292	1.967764052804	-3.564916968841
F	-7.960069547024	0.045712335974	-4.555668633080
F	-9.074781624554	0.476637126292	-2.757905614544
O	-7.420421533997	-0.833145197496	0.099165842582
C	-8.268627850950	-0.809430470712	1.011438899442
C	-8.776676695340	-1.898096062270	1.738043278414
C	-8.449767016067	-3.205597214490	1.377929517741
O	-7.694385376475	-3.586638366384	0.449376156780
H	-9.482596843559	-1.721126397293	2.543501386804
C	-8.834832761909	0.590348262599	1.309716978434
F	-9.814061400497	0.877453598606	0.442540228817
F	-7.886434082187	1.524064802446	1.180996117709
F	-9.340719043777	0.686971104346	2.547772575443
C	-9.109704412969	-4.358376690410	2.153061620437
F	-9.771818824959	-3.946779245284	3.243323297546
F	-8.190215492651	-5.244469961433	2.546266291520
F	-9.988467037317	-5.002794937842	1.367511072328
P	-7.979103928690	-4.821553101170	-2.688552398296
O	-6.677413626664	-4.175655523407	-2.203981284146
H	0.474184343180	0.104368324929	0.178553641234
C	-9.267332079694	-3.577071426449	-2.938140612370
C	-9.400538417295	-2.938222500252	-4.180422198685
C	-10.121032345812	-3.215660331262	-1.884987710223
C	-10.401232771427	-1.981257671587	-4.376187137376
H	-8.733708537604	-3.180066542094	-4.997924704973
C	-11.112068014362	-2.249817075644	-2.079171312929
H	-10.029902755773	-3.684742494209	-0.917424623591
C	-11.258238520469	-1.638796922376	-3.326902679011
H	-10.505665111306	-1.498009644818	-5.338914220127
H	-11.769908344512	-1.978769777548	-1.263633589963
H	-12.028103278168	-0.893182035441	-3.477911066598
C	-7.716989703489	-5.687127555165	-4.250177866226
C	-6.413755424961	-5.908872442895	-4.717997597028
C	-8.808893860440	-6.155803400866	-4.995992235032
C	-6.205432172288	-6.583935378063	-5.924554179851
H	-5.559597199952	-5.563181627690	-4.150957240923
C	-8.597586321255	-6.830728828620	-6.202197835371
H	-9.820841571809	-5.993057978359	-4.649654791693
C	-7.296683385993	-7.044267094357	-6.667008496597
H	-5.198116333826	-6.750981897476	-6.283410946687
H	-9.442526401418	-7.187773318021	-6.776664762571
H	-7.134581257801	-7.566888761927	-7.600880466488
C	-8.577405593828	-6.080239808267	-1.541321475794
C	-9.904296636777	-6.532546466600	-1.600393674828
C	-7.697600414979	-6.642555046861	-0.606303980961
C	-10.348132357549	-7.523597169575	-0.719224018137
H	-10.595374043888	-6.117720872294	-2.322082376030
C	-8.143358303710	-7.632706469958	0.273853345314
H	-6.668384676528	-6.316063422661	-0.559159987438
C	-9.468893838064	-8.072316971819	0.218829145957

H	-11.373702210711	-7.866312123116	-0.763953754220
H	-7.460992403008	-8.059061681508	0.997765481053
H	-9.813868260825	-8.838433697339	0.901099656916

ISC

Electronic energy = -3693.491349817377 (hartree)

Eu	-6.265131589340	-2.381645596376	-0.400765079201
P	-4.161189372157	-2.146221652935	2.658804293293
C	-2.747890498249	-1.730334203971	1.609585411971
C	-1.912300499360	-2.730715426888	1.089929462131
C	-0.864693253137	-2.396353427227	0.227076126237
C	-0.645452292796	-1.062080882758	-0.123804973110
C	-1.473046807109	-0.060511459401	0.390043512502
C	-2.520918387919	-0.392698115238	1.253545198405
C	-4.208449817642	-0.973294318282	4.028683400641
C	-3.022042833180	-0.490307821645	4.601089953803
C	-3.074513669123	0.417053568280	5.663862543235
C	-4.309617924602	0.840568595045	6.163326381367
C	-5.493885935424	0.353174535460	5.603241434776
C	-5.444163009053	-0.554315785172	4.540933353550
C	-3.905062467370	-3.761349116247	3.421234521104
C	-2.692688530984	-4.063365917834	4.058349969801
C	-4.936505305336	-4.711164835825	3.411242199095
H	-2.073161988525	-3.768719884241	1.337497052997
H	-0.226961041691	-3.172856513713	-0.174969637759
H	-1.304668039706	0.973232293679	0.117336816194
H	-3.160157666886	0.391825638828	1.638431194506
H	-2.059415960809	-0.810993369549	4.225076109316
H	-2.157678499877	0.791615131944	6.100251794834
H	-4.348944353831	1.543125735208	6.985740168915
H	-6.450227116337	0.676803843512	5.992807649678
H	-6.367628406139	-0.931571480977	4.121492249410
H	-1.891891843369	-3.336075214317	4.085997392380
H	-5.884634635886	-4.487350845535	2.940779431009
C	-2.505337876203	-5.314385015460	4.653903602615
C	-3.531042065014	-6.263773767049	4.627057443714
C	-4.747364881941	-5.960647906861	4.008806714019
H	-1.565270624264	-5.547421608137	5.136746272795
H	-3.384643953409	-7.232082295455	5.087888345647
H	-5.543589175866	-6.693589156567	3.991537252106
O	-5.463254195975	-2.088135069886	1.856016333479
O	-4.162599825773	-2.414524293129	-1.568384079689
C	-3.418344201523	-3.334283797105	-1.982479975101
C	-3.313240285072	-4.636502716087	-1.484142091573
C	-4.057935778214	-5.026561531195	-0.361510575314
O	-4.915078570227	-4.359647223652	0.254436235899
H	-2.598724461595	-5.324809328209	-1.925204546903
C	-2.552183923616	-2.946957690871	-3.192876617005
F	-2.052777016899	-1.718458795583	-3.054725635862
F	-3.300971258240	-2.970421805742	-4.303676002884
F	-1.524003997499	-3.790787295064	-3.383182242768
C	-3.742052231495	-6.402192973816	0.257757791479
F	-3.265640711099	-7.272932666464	-0.644437435413
F	-2.805153504598	-6.263730595618	1.216270814161
F	-4.818356066220	-6.944484108491	0.823260874968
O	-5.249398314362	-0.018883611978	-0.209594130548
C	-5.751583932196	0.930609756722	-0.816688846822
C	-6.494225223571	0.774741947777	-2.030577566560
C	-6.600725783003	-0.584787225090	-2.614104932577
O	-7.596363885583	-1.216685639629	-2.007058057133
H	-7.106266047958	1.610129806365	-2.388532403325
C	-5.547070754272	2.335366784484	-0.229425367223
F	-4.523262477852	2.940435325552	-0.849791524935
F	-6.638151235193	3.091633639104	-0.403156416252
F	-5.272918627083	2.278590394602	1.069024246078
C	-6.356483680276	-0.790872321202	-4.064252685899
F	-5.190954692883	-0.231469144890	-4.435658206190
F	-6.311298839194	-2.101181749499	-4.376022767493
F	-7.310107420494	-0.251030907969	-4.870390823076
O	-7.752149583173	-0.891506900003	0.845797205158
C	-8.492784364228	-1.077485572680	1.826221650952
C	-8.959631455507	-2.304350921442	2.336347807822
C	-8.662900489812	-3.503626748972	1.694005197832
O	-7.947229706710	-3.685368120415	0.674638712298

H	-9.603975594542	-2.310246112483	3.209676515831
C	-8.939299124935	0.223584291822	2.517120962257
F	-9.767268564105	0.910749067754	1.721615418360
F	-7.876956007450	0.998121080463	2.770199133743
F	-9.571717719362	0.009852737536	3.681007621787
C	-9.279093441557	-4.801967913710	2.236487250047
F	-9.962421953080	-4.627846961786	3.376252589263
F	-8.326046144100	-5.710493788304	2.468667604751
F	-10.126524401582	-5.320546350974	1.332431307575
P	-8.003745594182	-4.689764266603	-2.675094535353
O	-6.771192831160	-4.090640437786	-1.983610240494
H	0.163928724142	-0.804684647374	-0.794762931026
C	-9.308194158506	-3.457428214204	-2.898826835516
C	-9.444223785967	-2.772465688054	-4.116790159394
C	-10.154612854325	-3.129855285742	-1.828225922653
C	-10.413998036909	-1.775359229112	-4.259110113844
H	-8.794116474514	-2.995127597536	-4.952145810867
C	-11.122032899283	-2.131841170596	-1.972997817634
H	-10.066502154299	-3.642206364666	-0.881300356277
C	-11.251668480271	-1.454148245508	-3.187784851823
H	-10.512105544926	-1.247639883123	-5.198911016763
H	-11.770046095133	-1.882803058130	-1.142658541109
H	-12.000556570901	-0.680664176248	-3.298896116620
C	-7.564507247806	-5.370532955298	-4.287035769199
C	-6.225706480733	-5.378701232534	-4.704858090314
C	-8.554787289195	-5.901233081738	-5.126771034437
C	-5.882755522532	-5.906092674090	-5.953578446595
H	-5.447891958043	-4.977723961909	-4.068223077578
C	-8.208926635373	-6.428043645352	-6.374894619082
H	-9.592430011474	-5.899404539662	-4.820801323417
C	-6.873707803777	-6.430347042289	-6.788548829182
H	-4.848900451070	-5.908463116681	-6.273711483813
H	-8.976171605574	-6.833464633005	-7.021685292260
H	-6.607225689896	-6.838204664498	-7.755094976408
C	-8.675015176238	-6.072476041324	-1.728885609524
C	-10.007697463848	-6.479734299172	-1.893900901402
C	-7.848144665621	-6.763565286599	-0.834048122134
C	-10.508141044036	-7.558597572822	-1.158071668651
H	-10.659742724510	-5.963584493520	-2.586116743610
C	-8.350001938909	-7.841435377763	-0.099022375291
H	-6.816607226563	-6.468168534254	-0.708636917234
C	-9.680390729484	-8.238274371514	-0.259672464833
H	-11.537598802601	-7.867618263636	-1.284424508820
H	-7.707302350227	-8.368409618932	0.594284011934
H	-10.068961793387	-9.072581309271	0.309883258864

3.3. Model complex with dpbp linker

S0

Electronic energy = -2220.042341401596 (hartree)

Geometry of the high level region.

P	-4.758532768813	-0.004193970504	2.053818394296
C	-3.106860499785	-0.169360107089	1.278283476730
C	-2.953764698552	0.322450870317	-0.019684339026
C	-1.725122469309	0.316831265707	-0.655601876809
C	-0.584961623294	-0.148911206768	0.004020020081
C	-0.747190781176	-0.702801069195	1.282414905641
C	-1.988227740026	-0.713782449504	1.913493249561
C	-4.799867039919	1.735392221083	2.613043955985
C	-6.046759891651	2.365650359123	2.673575604691
C	-6.141969877233	3.675868488875	3.137376295475
C	-4.994576123747	4.361862105943	3.534735926864
C	-3.746385443824	3.748027413892	3.445762079940
C	-3.648021667491	2.437468763994	2.983721623083
C	-4.672866098367	-1.016227093532	3.577416760275
C	-4.095657792217	-0.548979829952	4.763730572210
C	-5.200349608341	-2.311467658726	3.534409942277
H	-3.785696994185	0.765227185627	-0.544428115012
H	-1.645335739639	0.764153174640	-1.647475593793
H	0.106301232665	-1.142559888422	1.800186073935
H	-2.074880617541	-1.152387649346	2.909671330299
H	-6.937959849224	1.825524176947	2.350843982767
H	-7.116065482544	4.166069452303	3.186974979487
H	-5.070782431955	5.385982588642	3.905352927654

H	-2.845795019306	4.293961925844	3.732138318724
H	-2.668029539568	1.961929228715	2.906126117401
H	-3.699297515074	0.466741297615	4.821984773232
H	-5.669959086831	-2.681122799255	2.624272138891
C	-4.031011311393	-1.373733647840	5.885363000162
C	-4.547721476016	-2.667578886777	5.832215518998
C	-5.134198237844	-3.133713238168	4.656760425696
H	-3.579687371984	-1.000498157259	6.806446724167
H	-4.498231282042	-3.312501250407	6.711729434105
H	-5.547713052910	-4.143203866358	4.611857138769
P	5.055556768063	-0.054029882342	-2.314568332732
O	6.023905948805	0.369934368757	-1.225799223399
O	-5.920600212173	-0.360227190781	1.145842282533
C	3.325338315660	0.041894929460	-1.750982557290
C	3.157472662730	0.036662545011	-0.368921943489
C	1.893166958885	0.025305832543	0.195553049562
C	0.754619126475	-0.017027690138	-0.615642938149
C	0.926817774473	0.040439551339	-2.008045210974
C	2.197044869062	0.076084574457	-2.573832954760
C	5.279063689592	-1.780045058935	-2.877332459377
C	6.580764551304	-2.180639880210	-3.204825047690
C	6.826862210465	-3.481887017244	-3.631482275824
C	5.775630201615	-4.394229518554	-3.731763911949
C	4.481677722128	-4.006868270876	-3.390734119318
C	4.232545999245	-2.703975808476	-2.960287078377
C	5.129263502705	0.958715441035	-3.833226944638
C	4.911186815188	0.417065385491	-5.104307414080
C	4.945843662198	1.238290233161	-6.231010099142
C	5.202501522368	2.600952695774	-6.093399558770
C	5.425760556573	3.144119120165	-4.828100785897
C	5.392259444562	2.327574203833	-3.701259259588
H	4.044716720328	0.048760965010	0.252634484204
H	1.792364855844	0.040689869329	1.281063997704
H	0.058641717224	0.015328076698	-2.668817159806
H	2.301978022618	0.111423554935	-3.660838484966
H	7.402688761109	-1.467395614757	-3.124242442770
H	7.844107288360	-3.787774089155	-3.883415267202
H	5.968220719876	-5.414525327344	-4.069289904203
H	3.659472798984	-4.722623371525	-3.452660954342
H	3.215967867951	-2.416475728879	-2.686839490323
H	4.722610506168	-0.652431018393	-5.221333019912
H	4.777981265025	0.808772313391	-7.220343115621
H	5.231779677851	3.243218776648	-6.975819679912
H	5.630606396519	4.210801976049	-4.717110097973
H	5.573728444804	2.760075229850	-2.717177358116

Geometry of the low level region (not optimized)

Tb	-6.700265	-1.068515	-0.615751
Eu	6.700945	1.067689	0.615132
P	-1.482279	7.424229	0.157970
O	-0.513161	8.491706	0.561851
C	-3.144299	7.760162	0.760580
C	-3.542528	9.079939	0.899582
H	-2.926786	9.778249	0.714833
C	-4.825151	9.389665	1.304312
H	-5.069709	10.300549	1.420217
C	-5.762831	8.401975	1.543974
C	-5.344254	7.085373	1.433193
H	-5.959590	6.386251	1.618114
C	-4.047863	6.763339	1.059568
H	-3.782143	5.852733	1.009359
C	-7.170304	8.753192	1.891281
H	-7.213711	9.771278	2.217651
H	-7.792155	8.628066	1.029571
H	-7.514678	8.112174	2.675757
C	-1.598091	7.305662	-1.631569
C	-0.520052	7.680780	-2.397802
H	0.270958	8.003699	-1.980827
C	-0.586713	7.589415	-3.787603
H	0.157066	7.854043	-4.316335
C	-1.719763	7.119165	-4.392955
H	-1.755513	7.051371	-5.340525
C	-2.795456	6.749370	-3.646093
H	-3.580840	6.424718	-4.073472

C	-2.747993	6.845185	-2.263936
H	-3.503405	6.595748	-1.745997
C	-0.994536	5.825936	0.813691
C	-1.273394	4.647711	0.161231
H	-1.699256	4.657514	-0.688872
C	-0.924218	3.442072	0.756949
H	-1.109783	2.626154	0.305901
C	-0.320203	3.412920	1.977875
H	-0.077822	2.580431	2.367366
C	-0.065135	4.577224	2.642464
H	0.335149	4.555299	3.503234
C	-0.390532	5.793680	2.058006
H	-0.198219	6.605469	2.513917
P	1.482959	-7.425055	-0.158589
O	0.513841	-8.492532	-0.562470
C	3.144980	-7.760988	-0.761199
C	3.543208	-9.080764	-0.900201
H	2.927467	-9.779075	-0.715451
C	4.825832	-9.390490	-1.304930
H	5.070390	-10.301374	-1.420835
C	5.763512	-8.402800	-1.544592
C	5.344935	-7.086198	-1.433812
H	5.960271	-6.387077	-1.618733
C	4.048543	-6.764165	-1.060186
H	3.782824	-5.853559	-1.009978
C	7.170985	-8.754017	-1.891899
H	7.792836	-8.628891	-1.030189
H	7.214392	-9.772103	-2.218269
H	7.515358	-8.112999	-2.676375
C	1.598771	-7.306488	1.630950
C	0.520732	-7.681606	2.397184
H	-0.270276	-8.004525	1.980209
C	0.587394	-7.590241	3.786984
H	-0.156385	-7.854868	4.315717
C	1.720443	-7.119990	4.392337
H	1.756194	-7.052198	5.339906
C	2.796136	-6.750196	3.645474
H	3.581520	-6.425544	4.072853
C	2.748674	-6.846011	2.263317
H	3.504085	-6.596574	1.745378
C	0.995216	-5.826762	-0.814310
C	1.274074	-4.648537	-0.161849
H	1.699937	-4.658340	0.688253
C	0.924899	-3.442899	-0.757567
H	1.110464	-2.626980	-0.306520
C	0.320884	-3.413746	-1.978493
H	0.078504	-2.581256	-2.367984
C	0.065816	-4.578049	-2.643082
H	-0.334467	-4.556124	-3.503853
C	0.391212	-5.794506	-2.058624
H	0.198899	-6.606294	-2.514536
P	-8.296998	-3.981966	-2.275110
O	-7.327880	-2.914489	-1.871229
C	-8.412810	-4.100532	-4.064649
H	-7.438587	-3.990341	-4.493191
H	-8.814891	-5.054945	-4.333586
H	-9.053903	-3.326901	-4.432610
C	-9.959019	-3.646032	-1.672500
H	-9.998888	-2.655633	-1.269487
H	-10.655271	-3.730572	-2.480575
H	-10.211134	-4.352146	-0.909125
C	-7.809256	-5.580259	-1.619389
H	-7.456004	-5.461548	-0.616383
H	-8.651465	-6.240221	-1.625149
H	-7.029571	-5.991694	-2.225783
P	8.297678	3.981140	2.274492
O	7.328561	2.913663	1.870610
C	9.959700	3.645206	1.671881
H	10.655952	3.729746	2.479956
H	9.999569	2.654807	1.268868
H	10.211815	4.351320	0.908506
C	8.413491	4.099706	4.064030
H	8.815572	5.054119	4.332967
H	7.439268	3.989515	4.492572

H	9.054584	3.326075	4.431991
C	7.809936	5.579433	1.618771
H	8.652146	6.239395	1.624531
H	7.456684	5.460723	0.615765
H	7.030251	5.990868	2.225164
O	-4.930331	-1.216462	-2.253022
C	-3.997448	-2.015253	-2.477260
C	-3.508199	-3.026733	-1.653283
C	-4.055044	-3.236166	-0.396561
O	-5.033041	-2.661967	0.127091
H	-2.794415	-3.577122	-1.954105
C	-3.297724	-1.801651	-3.827069
F	-2.763097	-0.575942	-3.881767
F	-4.149290	-1.886445	-4.826430
F	-2.316554	-2.659176	-4.057983
C	-3.351793	-4.255943	0.518640
F	-2.517363	-5.065109	-0.118074
F	-2.646346	-3.626150	1.436531
F	-4.218984	-5.018336	1.146549
O	-5.975274	1.179875	-1.097468
C	-6.247702	2.047851	-1.940251
C	-7.233604	2.017749	-2.915925
C	-8.030393	0.893315	-3.076001
O	-7.966391	-0.185532	-2.467827
H	-7.364045	2.772570	-3.478489
C	-5.355320	3.298314	-1.840543
F	-4.080222	2.975836	-1.893452
F	-5.587457	4.200719	-2.760513
F	-5.514583	3.903938	-0.673062
C	-9.145572	0.932099	-4.136576
F	-9.268396	2.083743	-4.747476
F	-8.934646	0.027520	-5.081247
F	-10.310924	0.619508	-3.610354
O	-8.637292	0.046347	0.300884
C	-9.397318	-0.155134	1.270620
C	-9.513737	-1.301106	2.054578
C	-8.737639	-2.418289	1.786576
O	-7.836585	-2.547240	0.931082
H	-10.129516	-1.317840	2.778074
C	-10.336569	1.017333	1.587399
F	-11.127162	1.272815	0.538174
F	-9.660980	2.120445	1.827814
F	-11.131341	0.796238	2.622202
C	-9.043365	-3.698858	2.585809
F	-9.773405	-3.493763	3.672733
F	-7.940609	-4.299604	2.973839
F	-9.710395	-4.550958	1.833602
O	4.931012	1.215637	2.252403
C	3.998129	2.014427	2.476642
C	3.508880	3.025907	1.652665
C	4.055724	3.235341	0.395943
O	5.033722	2.661141	-0.127710
H	2.795096	3.576297	1.953487
C	3.298406	1.800826	3.826450
F	2.763777	0.575116	3.881148
F	2.317236	2.658350	4.057365
F	4.149971	1.885620	4.825812
C	3.352473	4.255117	-0.519258
F	4.219666	5.017510	-1.147168
F	2.518044	5.064283	0.117456
F	2.647028	3.625325	-1.437149
O	8.637973	-0.047173	-0.301503
C	9.397999	0.154308	-1.271239
C	9.514418	1.300280	-2.055198
C	8.738320	2.417463	-1.787195
O	7.837266	2.546414	-0.931701
H	10.130196	1.317014	-2.778693
C	10.337249	-1.018159	-1.588018
F	11.127843	-1.273640	-0.538792
F	11.132022	-0.797065	-2.622821
F	9.661661	-2.121271	-1.828433
C	9.044047	3.698033	-2.586428
F	9.774086	3.492938	-3.673352
F	9.711075	4.550132	-1.834221

F	7.941289	4.298778	-2.974457
O	5.975955	-1.180700	1.096849
C	6.248383	-2.048677	1.939631
C	7.234284	-2.018574	2.915306
C	8.031074	-0.894141	3.075382
O	7.967071	0.184706	2.467209
H	7.364726	-2.773396	3.477870
C	5.356001	-3.299139	1.839925
F	4.080902	-2.976661	1.892834
F	5.588138	-4.201545	2.759895
F	5.515264	-3.904763	0.672444
C	9.146253	-0.932925	4.135957
F	9.269077	-2.084569	4.746857
F	8.935326	-0.028346	5.080629
F	10.311605	-0.620334	3.609735

Linker-centered T1
Electronic energy = -2219.930190433919 (hartree)
Geometry of the high level region. That of the low level region is the same as S0.

P	-4.882796519004	0.011692697594	2.172968858699
C	-3.203411548360	-0.174210334986	1.505421951925
C	-3.054819059902	0.076428106465	0.114517141307
C	-1.842689640560	0.086315470859	-0.493567020364
C	-0.617888874627	-0.109061707320	0.272650576409
C	-0.805675444687	-0.491847538726	1.678553186033
C	-2.043073612261	-0.509577369096	2.256890641556
C	-4.952124965514	1.758070744599	2.715822486522
C	-6.209739081974	2.361631472893	2.819414032761
C	-6.316608118868	3.677959727419	3.261868695284
C	-5.169562963410	4.399557302342	3.593085473800
C	-3.912319938375	3.813594436239	3.459490437997
C	-3.802664641653	2.495227923714	3.021843519105
C	-4.899024444342	-0.982251199571	3.708407271039
C	-4.525817804981	-0.452283271954	4.948191018812
C	-5.283044257557	-2.325779142374	3.620663565385
H	-3.938912912959	0.259786171323	-0.475288171404
H	-1.789720069468	0.278907643202	-1.562506459941
H	0.056271341817	-0.768859843161	2.283181678432
H	-2.139791572803	-0.789190078158	3.308549213931
H	-7.102675811118	1.796641069998	2.548559570470
H	-7.299360828619	4.145715399688	3.346359214967
H	-5.254891984667	5.429038253210	3.946446715739
H	-3.011705323864	4.385365039720	3.691458855337
H	-2.813941493802	2.044504265773	2.915296120137
H	-4.245074150325	0.599434655948	5.035336067635
H	-5.592036762047	-2.748006059744	2.664914411766
C	-4.521553951836	-1.261181136372	6.083951725260
C	-4.894992071070	-2.600517448787	5.988708417798
C	-5.277700275574	-3.130588663537	4.756798019934
H	-4.230621791068	-0.840033049524	7.047962025446
H	-4.892726375892	-3.233107789172	6.878597283292
H	-5.576613507895	-4.177816913293	4.678657858097
P	4.881447960829	-0.016768109102	-2.192395978711
O	5.963238858935	0.348778044795	-1.191631026314
O	-5.963419897862	-0.356782633254	1.171708769929
C	3.204260983723	0.172502719925	-1.521689617994
C	3.058640592765	-0.073000979949	-0.129163499624
C	1.847542746913	-0.082031776193	0.480832362354
C	0.621370669324	0.110745668326	-0.284807738197
C	0.807143864013	0.492433019548	-1.691701689693
C	2.043178379511	0.507650919685	-2.272182017787
C	4.947518378514	-1.763015543383	-2.736155350443
C	6.203861079428	-2.369900606047	-2.836490315646
C	6.307999019355	-3.687925615361	-3.274604376627
C	5.159600729686	-4.407937687635	-3.604552283771
C	3.903663095869	-3.818340257268	-3.474804600829
C	3.796655552989	-2.498263846080	-3.041642327985
C	4.897662937046	0.980995724236	-3.725015877582
C	4.524950092774	0.455608596198	-4.966821398710
C	4.520675372612	1.269087704756	-6.099390744936
C	4.893576162887	2.608178432101	-5.998636748704
C	5.275751165571	3.133649322946	-4.764548079269
C	5.281086886763	2.324355465959	-3.631661562589
H	3.945473608657	-0.251463152186	0.458435132656

H	1.795608098284	-0.270312554276	1.550699032029
H	-0.055320148052	0.770141836107	-2.295133125317
H	2.138596086616	0.785491896271	-3.324409820182
H	7.097949401612	-1.806581980252	-2.565819301204
H	7.289741164894	-4.158303676758	-3.356247054211
H	5.242896020573	-5.439039886675	-3.953681287090
H	3.002023147840	-4.388774538345	-3.706020000515
H	2.808754642115	-2.045180189125	-2.937348156361
H	4.244424920461	-0.595863028753	-5.058013668991
H	4.230036073513	0.851784197794	-7.065160500052
H	4.891150021880	3.244358772900	-6.885970593182
H	5.574086294900	4.180727564818	-4.682219865198
H	5.589407473459	2.742686799706	-2.673929943661

ISC between the ground state and linker-centered T1
Electronic energy = -2219.903902212561 (hartree)
Geometry of the high level region. That of the low level region is the same as S0.

P	-4.868223201069	-0.028462500165	2.141548435473
C	-3.196525226632	-0.259250568451	1.450589089057
C	-3.008819676841	0.099410777649	0.112544114641
C	-1.747975620127	0.115567930508	-0.451316492740
C	-0.617082468614	-0.215607244537	0.313769681985
C	-0.820230078776	-0.673952646596	1.633099431921
C	-2.088784349412	-0.684818085755	2.194699517562
C	-4.879090646911	1.721626846331	2.674977956875
C	-6.117670411583	2.367656590688	2.744835288186
C	-6.192076594310	3.686944285001	3.185632211697
C	-5.030738041124	4.368189965951	3.549604873345
C	-3.790998727736	3.739513589617	3.449815657849
C	-3.713825205930	2.418689924681	3.012589209511
C	-4.887930975227	-1.010672239496	3.685970633278
C	-4.523599055924	-0.471347464835	4.924334686768
C	-5.276617793708	-2.353769319541	3.608912977837
H	-3.849170051188	0.412549955747	-0.491119130415
H	-1.613200571848	0.462118137664	-1.478908511330
H	0.026297758726	-1.000036201766	2.238506702106
H	-2.214438085818	-1.017393823044	3.227439606565
H	-7.020654847646	1.832405419500	2.447909465450
H	-7.160384598573	4.187595877343	3.243352485901
H	-5.089700941428	5.399681799586	3.902621587068
H	-2.879381631492	4.280823956776	3.709229248288
H	-2.739423822021	1.932859057972	2.930124544709
H	-4.238572971258	0.579560599160	5.004365637692
H	-5.580294568289	-2.785428518681	2.655721309118
C	-4.532532220779	-1.269247203866	6.067846289093
C	-4.909578202387	-2.608030739802	5.982605528702
C	-5.283254282240	-3.148093209700	4.752263013624
H	-4.248629235413	-0.839157117873	7.030026351143
H	-4.917174806524	-3.231790080531	6.878684083361
H	-5.585263232542	-4.194973307332	4.681410008747
P	4.923673597683	-0.104674763014	-2.232428291941
O	5.944739247402	0.328451437023	-1.191377296233
O	-5.985199277666	-0.362444769594	1.172238286182
C	3.211296640923	0.127312466915	-1.620323605007
C	3.105091222151	0.079081464132	-0.181994386171
C	1.883788051959	-0.054959484010	0.484023967017
C	0.695458662767	0.048276872315	-0.225166591541
C	0.917850258149	0.731898057445	-1.543019832616
C	2.077414630139	0.272693661288	-2.358519417358
C	5.039989283900	-1.855778646025	-2.742012241004
C	6.317395998469	-2.390674310418	-2.945849082504
C	6.460885920696	-3.697636821865	-3.403380980983
C	5.331737036090	-4.478761614313	-3.653217889662
C	4.059258563941	-3.962461682525	-3.418171219330
C	3.912949017665	-2.653555338143	-2.962390878144
C	4.993849692333	0.853385955562	-3.785809821362
C	4.696448964631	0.288151082094	-5.029701114761
C	4.728899292812	1.074639353975	-6.181016987869
C	5.063502262727	2.424594090509	-6.095019642199
C	5.369468875529	2.989696329626	-4.856733667313
C	5.338117516680	2.208306191520	-3.705213369506
H	4.032010759764	-0.007524006869	0.365094714512
H	1.892881025867	-0.326781947477	1.541409277099
H	0.969516658025	1.827454243037	-1.387868888350

H	2.036284001065	0.169994093622	-3.445617134197
H	7.198419666301	-1.778425134058	-2.748415363307
H	7.457990091928	-4.111234321100	-3.565802775782
H	5.445571142588	-5.500190105637	-4.021771475147
H	3.174802253699	-4.580299412066	-3.585256357004
H	2.914666683695	-2.253451453251	-2.779464091232
H	4.448040790857	-0.772566670183	-5.107638884452
H	4.498655262681	0.627458896428	-7.149661092321
H	5.089908907742	3.039033944572	-6.997118357338
H	5.636224576686	4.046156591943	-4.786466038147
H	5.583232753835	2.655623776718	-2.741724404496

EET between Tb $^5D_4$ and linker-centered T1
Electronic energy = -2219.929482738095 (hartree)
Geometry of the high level region. That of the low level region is the same as S0.

P	-4.888292877005	0.014574390887	2.176058823204
C	-3.206132287974	-0.165805925349	1.511151782110
C	-3.058118650049	0.076297336171	0.123833057242
C	-1.840392075828	0.092447877114	-0.482127643826
C	-0.624685517796	-0.096233655143	0.281019058176
C	-0.805577124629	-0.448126004881	1.684472707911
C	-2.045761183283	-0.473955330304	2.265838853868
C	-4.963111768767	1.760827621419	2.719477390712
C	-6.223960847627	2.355892245144	2.833718521218
C	-6.336338375486	3.671469458043	3.276926471814
C	-5.191644089335	4.401087859077	3.598673016844
C	-3.931629050882	3.823645347144	3.454870900888
C	-3.816464555078	2.506051121588	3.016270618197
C	-4.906258380397	-0.978022389346	3.712159407618
C	-4.546752679268	-0.442962006690	4.953743558863
C	-5.280262568058	-2.324417652479	3.624113739549
H	-3.941368018565	0.252657058411	-0.469517434197
H	-1.789051084414	0.285839783524	-1.551034358136
H	0.058730165790	-0.696877284306	2.297883851975
H	-2.136642074890	-0.734101628013	3.322979476324
H	-7.115316596581	1.784754981651	2.570697332269
H	-7.321637028348	4.132299772860	3.369516978081
H	-5.281351188365	5.429974195823	3.952716589502
H	-3.032999483419	4.401431182857	3.679597550278
H	-2.825573207464	2.062338970769	2.901924995821
H	-4.274458611272	0.610997961366	5.040775908035
H	-5.579044981324	-2.750772145843	2.666943146254
C	-4.546144721868	-1.249466072017	6.091271499673
C	-4.909493929695	-2.591498574310	5.995770048974
C	-5.278340895955	-3.126846256338	4.761863268625
H	-4.266650277953	-0.824168606161	7.056798441259
H	-4.910259496366	-3.222164814154	6.887042235993
H	-5.569267800639	-4.176301561375	4.683534536215
P	4.887302897571	-0.018555953303	-2.196043898912
O	5.965593980862	0.349977681364	-1.192895840674
O	-5.965495640576	-0.356917631465	1.172475271518
C	3.207204661202	0.164495882149	-1.528093163523
C	3.062315849043	-0.071594702267	-0.139148281687
C	1.845417839580	-0.088267381690	0.468572869331
C	0.628303270179	0.095856859604	-0.294189596779
C	0.806843910616	0.444690468965	-1.698973814160
C	2.045734529310	0.469361526996	-2.282353301695
C	4.959291119961	-1.764704227806	-2.740271173307
C	6.219179042934	-2.362316745057	-2.852579593557
C	6.329257291024	-3.679459091934	-3.291707873540
C	5.183343966490	-4.408249635100	-3.610987349382
C	3.924363938126	-3.828014123611	-3.469585772168
C	3.811428758199	-2.508781067434	-3.035323659659
C	4.905108171550	0.977369357470	-3.729702785647
C	4.546492393253	0.445609034766	-4.972894579502
C	4.545939298279	1.255515279086	-6.108031408484
C	4.908466771337	2.597462018552	-6.008325037447
C	5.276243250368	3.129543190325	-4.772629714237
C	5.278021565370	2.323831437313	-3.637214755897
H	3.948636741160	-0.241314376087	0.452013774751
H	1.795200004155	-0.276405727018	1.538597519724
H	-0.058493329280	0.690559397501	-2.311937858033
H	2.134903938573	0.726017402172	-3.340499310142
H	7.111672180603	-1.792400198257	-2.590580102330

H	7.313739820940	-4.142340708722	-3.382649924625
H	5.271247249828	-5.438672907097	-3.960992643828
H	3.024743019436	-4.404947023078	-3.692499050977
H	2.821186408582	-2.063285339692	-2.922202528770
H	4.274705740416	-0.608249559054	-5.062947667624
H	4.267052399259	0.833019437334	-7.074968652407
H	4.909313114437	3.230768915948	-6.897718584463
H	5.566346250216	4.178988195936	-4.691136423472
H	5.575504381015	2.747486589249	-2.678351872923

EET between Eu $^5D_0$ and linker-centered T1
Electronic energy = -2219.929815748300 (hartree)
Geometry of the high level region. That of the low level region is the same as S0.

P	-4.876632115206	0.011502479289	2.167708986437
C	-3.200251878751	-0.182450249408	1.496781222667
C	-3.050787984132	0.079206515619	0.103230617939
C	-1.843762548891	0.079088180000	-0.507271568564
C	-0.612695757290	-0.126999349307	0.262107540167
C	-0.808018811349	-0.549946956108	1.665283189422
C	-2.042743915388	-0.554965810598	2.242259276232
C	-4.938762510214	1.758448098640	2.708450819626
C	-6.192631202931	2.370722876599	2.804607547602
C	-6.292654741341	3.687435976656	3.247629412647
C	-5.142455140803	4.400310362231	3.586595845629
C	-3.888571505146	3.805565807995	3.460141644976
C	-3.785748387162	2.486801673056	3.022054030967
C	-4.893351269639	-0.980625856336	3.704471914481
C	-4.517706853476	-0.450484653783	4.943417644317
C	-5.281961012738	-2.322961780000	3.618653648268
H	-3.934574369718	0.273935949743	-0.483144543764
H	-1.787655396429	0.272055554478	-1.576041690258
H	0.049834286787	-0.865389860382	2.256943939316
H	-2.146957627709	-0.859642241438	3.286221558219
H	-7.087772878925	1.812178049313	2.527765298138
H	-7.272497569922	4.162174735961	3.326581088384
H	-5.222523186241	5.430014748443	3.940556957128
H	-2.985602015494	4.370904299294	3.698427264591
H	-2.799783223376	2.028422919897	2.921404230060
H	-4.233158483602	0.600270362630	5.029268643374
H	-5.593056050932	-2.745338360858	2.663697797934
C	-4.515751245068	-1.257963272278	6.080157251140
C	-4.893607658314	-2.596177980107	5.986792178069
C	-5.278593124342	-3.126487673070	4.755708593486
H	-4.223189019354	-0.836469339362	7.043491697072
H	-4.893102539271	-3.227547552097	6.877531536105
H	-5.581106816170	-4.172809301166	4.678921832391
P	4.874457190998	-0.015344524654	-2.187048709604
O	5.960613446205	0.347662627818	-1.189957677990
O	-5.961255478710	-0.354917961658	1.169881442569
C	3.200361357827	0.181949806671	-1.512660828585
C	3.053915590572	-0.075735877892	-0.117600729596
C	1.847841519574	-0.075379500962	0.494669250533
C	0.615420833746	0.128617080267	-0.274061243774
C	0.808756570974	0.551425293807	-1.677979946310
C	2.042143224899	0.554656061586	-2.256999395818
C	4.932851249904	-1.762114675039	-2.728787103177
C	6.185304762238	-2.377804750552	-2.822479815210
C	6.282263937281	-3.696022161230	-3.261771253354
C	5.130483292021	-4.407072879455	-3.599210075119
C	3.878086839494	-3.808587513238	-3.475892356087
C	3.778257793919	-2.488289186753	-3.041691821782
C	4.891080959986	0.980070129072	-3.721371202984
C	4.515046114139	0.453839681116	-4.961790745025
C	4.513487625975	1.264918254220	-6.096009372485
C	4.892135396215	2.602616023027	-5.998413613288
C	5.277257580034	3.129042194428	-4.765676442955
C	5.280168858382	2.322020480463	-3.631098687170
H	3.940398494284	-0.266199029797	0.466523330322
H	1.792627432898	-0.265290270526	1.564152974554
H	-0.049608131590	0.867892449545	-2.268246333565
H	2.145158183144	0.858486306360	-3.301335392130
H	7.081827185821	-1.821148611036	-2.546076565935
H	7.260970930469	-4.173444241971	-3.338641253201
H	5.208138789025	-5.438284201036	-3.949323849634

H	2.973899586479	-4.372300012539	-3.713322437659
H	2.793318296538	-2.027376801189	-2.942738454243
H	4.229761724966	-0.596494024180	-5.050744539164
H	4.220453609402	0.846760009657	-7.060668843414
H	4.892009178532	3.236703598436	-6.887221060615
H	5.580134546481	4.175006490010	-4.685743630544
H	5.591133138856	2.741058326311	-2.674524378718

3.4. Model complex with dpb linker

S0

Electronic energy = -1989.474599482902 (hartree)

Geometry of the high level region.

O	21.467659852717	14.677202207293	15.742271708431
O	25.872451036860	20.778169966841	12.557719036538
P	25.203732663882	20.616625205536	13.907182693361
P	22.439723430925	14.806780574189	14.594993315182
C	21.662280741341	14.392343400881	12.997207592120
C	20.265627517641	14.431287395171	12.953234217202
C	22.387672196881	14.038188604876	11.855029455783
C	23.204060662636	17.124877051244	13.145883616472
C	25.135500815389	13.933303257766	14.230926991654
C	21.718696644243	13.749916449350	10.668206910898
C	20.325166202384	13.804084689273	10.621939054542
C	26.143964365631	12.980148723381	14.367154814207
C	19.599483319544	14.138822390776	11.764439231172
H	19.714799053479	14.680827761843	13.862303100553
H	23.478590595205	13.981979983097	11.887482200167
H	25.349334745675	14.879452313002	13.728473017227
H	22.289263988717	13.483882921723	9.776116953270
H	19.804303736785	13.577680502497	9.689774845254
H	27.138857788205	13.178478215267	13.965894497525
H	18.508885398411	14.168646121271	11.730801805977
H	22.769955314630	16.661951729458	12.258059581584
C	23.887275109665	21.848251812437	14.177486188818
C	24.385025459307	18.992237172586	14.145160898450
C	23.801594631303	18.375863652714	13.037407033552
C	21.937590687109	23.815710090495	14.484748310003
C	23.919899057781	22.993938797227	13.377977971297
C	22.944264300155	23.976339153389	13.533486043272
H	21.173777685264	24.586497848954	14.605569035556
H	24.705987130919	23.094781401870	12.628443304310
H	22.966760219548	24.869205896873	12.905864316628
H	23.817865728071	18.861413972722	12.069097086924
C	23.863885540889	13.677403043669	14.752809668180
C	23.171176635301	16.469641451768	14.377281235563
C	23.719310869891	17.104090762265	15.496069828006
C	25.887312173060	11.777586078950	15.024010708458
C	23.615238833355	12.478426431944	15.429146142216
C	24.623812664681	11.528296261724	15.560017821405
H	26.681416911521	11.036305717889	15.130135268967
H	22.625620924738	12.309986083125	15.856554771519
H	24.428032748945	10.594288651556	16.089678210975
H	23.675400630748	16.624412917495	16.467568461701
C	26.348883615681	20.733825700756	15.327813279144
C	27.592780279975	20.101499902101	15.203480148609
C	26.026834719249	21.393729967886	16.517659253438
C	24.334885380576	18.344856796318	15.383297199893
C	22.865703155562	21.679969231645	15.117408584521
C	26.931760249323	21.405645855500	17.578831667113
C	28.161990295102	20.763513684072	17.454807466532
C	21.897037344265	22.667575142368	15.276508086629
C	28.492290905462	20.113857759494	16.265123205701
H	27.860543961689	19.594281893679	14.275443341256
H	25.068872157596	21.905885679160	16.622118907345
H	22.822557905754	20.769811778580	15.718273734135
H	26.674537020339	21.921419972504	18.505668823066
H	28.869151371971	20.770456789827	18.286402451790
H	21.105043048356	22.541149127608	16.016194785600
H	29.456200007885	19.611643484338	16.162658073605
H	24.791959664811	18.796140329317	16.265923875968

Geometry of the low level region (not optimized)

Tb	21.258037	15.384348	18.169857

Eu	26.357012	20.274616	10.565185
F	19.477503	20.390475	15.848381
F	24.295142	19.366775	19.133228
F	22.852295	20.832126	19.115987
F	20.108458	18.876501	14.493812
F	18.368259	18.586801	15.690770
F	31.314698	21.895906	11.453059
F	23.360363	20.586886	5.641551
F	21.671920	22.264993	10.999630
F	21.435110	20.177571	11.367583
F	20.699904	21.027251	9.540034
F	26.540403	25.112542	9.064038
F	25.474938	25.062125	10.886271
F	25.438373	20.702688	5.699165
F	24.522299	18.876195	6.114817
F	27.436320	25.906470	10.848915
F	30.376001	21.269380	13.346815
F	30.603010	23.413106	12.885400
F	24.754670	16.309123	8.136327
F	28.137545	15.268490	12.886661
F	24.762754	14.826838	9.619055
F	23.319907	16.292190	9.601814
F	27.506591	16.782463	14.241231
F	29.246790	17.072163	13.044272
P	18.509843	16.253138	20.551302
P	29.105205	19.405827	8.183740
O	24.002810	20.780731	10.630385
O	25.530028	20.514012	8.317214
O	25.234530	18.218660	10.047639
O	27.227714	18.494150	11.960818
O	27.997604	19.401187	9.190760
O	26.524119	22.604205	10.210997
O	28.505837	21.071934	11.451489
O	23.612238	14.878234	18.104657
O	22.085021	15.144953	20.417828
O	22.380519	17.440305	18.687404
O	20.387334	17.164815	16.774225
O	19.617445	16.257778	19.544283
O	21.090929	13.054760	18.524045
O	19.109211	14.587031	17.283553
C	28.027447	16.590484	13.045709
C	24.597149	16.077667	9.392049
H	26.423729	15.496889	11.218160
C	26.356500	16.442361	11.156380
C	25.463034	17.014948	10.261284
C	27.153207	17.251801	11.963835
C	24.402513	20.476487	7.801564
C	23.081466	20.812749	9.787155
C	23.167886	20.633578	8.416494
C	24.415125	20.169209	6.297284
C	21.705113	21.076551	10.416453
H	22.375134	20.618876	7.893516
C	28.985404	22.191274	11.679358
C	27.239093	23.526240	10.665011
C	28.424994	23.436128	11.366146
C	26.680759	24.919553	10.366166
C	30.361429	22.192452	12.361815
H	28.867457	24.230506	11.640566
C	22.152015	18.644016	18.473759
C	23.212536	15.182478	20.933478
C	18.629645	13.467691	17.055684
C	20.375956	12.132724	18.070031
C	20.461842	18.407164	16.771207
C	21.258548	19.216604	17.578662
C	19.587602	19.068481	15.689333
C	23.017900	19.581298	19.342994
H	21.191320	20.162076	17.516882
C	19.190055	12.222837	17.368896
C	17.253620	13.466512	16.373227
C	20.934290	10.739412	18.368876
H	18.747592	11.428459	17.094477
C	24.533583	14.846216	18.947887
C	24.447163	15.025387	20.318548
C	23.199924	15.489756	22.437758

C	25.909936	14.582414	18.318589
H	25.239915	15.040088	20.841526
F	16.368256	13.824298	17.085856
F	24.254686	15.072079	23.093491
F	25.943129	13.393972	17.735412
F	26.179939	15.481393	17.367459
F	26.915145	14.631713	19.195008
F	21.074646	10.546422	19.671004
F	22.140111	10.596839	17.848771
F	22.176676	14.956276	23.035878
F	23.092749	16.782770	22.620225
F	20.178728	9.752494	17.886127
F	17.198015	14.276573	15.426407
F	16.930180	12.416793	15.834445
F	22.860378	19.349841	20.598715
H	18.918404	16.884375	21.672515
H	17.435373	16.891890	20.041392
H	18.176888	14.978964	20.848157
H	29.935674	18.368828	8.423441
H	28.585909	19.289007	6.943101
H	29.792899	20.563829	8.276520

Linker-centered T1
Electronic energy = -1989.344500144439 (hartree)
Geometry of the high level region. That of the low level region is the same as S0.

O	21.471836097672	14.669089292331	15.734981227905
O	25.897479882950	20.785087247213	12.556063415290
P	25.252849497368	20.602015085079	13.916139202090
P	22.442467269044	14.797314986100	14.581551043717
C	21.666160484821	14.355741829482	12.992060806409
C	20.269597898645	14.307033223723	12.970411848233
C	22.395016802772	14.058384762381	11.836381601089
C	23.023916208176	17.192977189256	13.168563362531
C	25.137166385182	13.937721217214	14.229496349606
C	21.727251196519	13.735578061318	10.658019474678
C	20.332238854279	13.699345426263	10.634204884922
C	26.154746379494	12.994798719055	14.371390209672
C	19.604575378895	13.980254148537	11.789682535142
H	19.720131176343	14.517645986132	13.889996964049
H	23.487459943940	14.074067256767	11.852920410675
H	25.335759357043	14.878031870964	13.708718789914
H	22.298901436138	13.512418395106	9.754992333556
H	19.812209269739	13.445107888692	9.708732619541
H	27.145428567132	13.194105055520	13.960650195573
H	18.513894824130	13.939918740570	11.773111844828
H	22.475341119360	16.790166501860	12.314290574467
C	23.910036308030	21.807374944402	14.185930020659
C	24.498508520788	18.979525109878	14.157233697326
C	23.677521118282	18.390342195361	13.032983326810
C	21.919187907018	23.729514136742	14.509277998440
C	23.893461844862	22.939075124898	13.366145769446
C	22.898144406963	23.899678227246	13.530790988826
H	21.140287892263	24.483883851451	14.637419201470
H	24.659846383031	23.047101772737	12.597313061386
H	22.883375850836	24.782957835398	12.889455106914
H	23.644490956499	18.905131526016	12.081005094044
C	23.872138525961	13.678777595563	14.765344747770
C	23.129208938679	16.443989611026	14.352413322437
C	23.856212744644	17.085488389170	15.511201660828
C	25.910664414385	11.797410864581	15.042232715459
C	23.638057119039	12.488191151939	15.460873946661
C	24.653185440607	11.545210200913	15.591636823013
H	26.710224059906	11.062082299487	15.148868554426
H	22.652861529443	12.319860489583	15.898235092223
H	24.467954708770	10.614761474329	16.131296923284
H	23.791300577166	16.621026930699	16.487483025420
C	26.408939168656	20.752188758290	15.323783559430
C	27.660294478478	20.136237529191	15.190738665189
C	26.092051296039	21.420961429572	16.509741701425
C	24.550948841038	18.260320725551	15.361030916123
C	22.916034985902	21.628580883923	15.153268297195
C	27.010177181308	21.458412337873	17.558955574888
C	28.247964694155	20.832502421246	17.426757757761
C	21.926333682644	22.594197593396	15.320149295910

C	28.572914452322	20.173658418324	16.240612234132
H	27.922264849759	19.621097782145	14.265192533364
H	25.127882162678	21.919817951577	16.620524390349
H	22.915400200300	20.728445364159	15.771558652151
H	26.756946279096	21.980607825562	18.483307966296
H	28.965001771691	20.858805504908	18.249475996229
H	21.156465266100	22.462614244045	16.081902615572
H	29.542400893112	19.683583803541	16.132119028488
H	25.098775551682	18.663632481386	16.215455329586

ISC between the ground state and linker-centered T1
Electronic energy = -1989.344500144439 (hartree)
Geometry of the high level region. That of the low level region is the same as S0.

O	21.471836097672	14.669089292331	15.734981227905
O	25.897479882950	20.785087247213	12.556063415290
P	25.252849497368	20.602015085079	13.916139202090
P	22.442467269044	14.797314986100	14.581551043717
C	21.666160484821	14.355741829482	12.992060806409
C	20.269597898645	14.307033223723	12.970411848233
C	22.395016802772	14.058384762381	11.836381601089
C	23.023916208176	17.192977189256	13.168563362531
C	25.137166385182	13.937721217214	14.229496349606
C	21.727251196519	13.735578061318	10.658019474678
C	20.332238854279	13.699345426263	10.634204884922
C	26.154746379494	12.994798719055	14.371390209672
C	19.604575378895	13.980254148537	11.789682535142
H	19.720131176343	14.517645986132	13.889996964049
H	23.487459943940	14.074067256767	11.852920410675
H	25.335759357043	14.878031870964	13.708718789914
H	22.298901436138	13.512418395106	9.754992333556
H	19.812209269739	13.445107888692	9.708732619541
H	27.145428567132	13.194105055520	13.960650195573
H	18.513894824130	13.939918740570	11.773111844828
H	22.475341119360	16.790166501860	12.314290574467
C	23.910036308030	21.807374944402	14.185930020659
C	24.498508520788	18.979525109878	14.157233697326
C	23.677521118282	18.390342195361	13.032983326810
C	21.919187907018	23.729514136742	14.509277998440
C	23.893461844862	22.939075124898	13.366145769446
C	22.898144406963	23.899678227246	13.530790988826
H	21.140287892263	24.483883851451	14.637419201470
H	24.659846383031	23.047101772737	12.597313061386
H	22.883375850836	24.782957835398	12.889455106914
H	23.644490956499	18.905131526016	12.081005094044
C	23.872138525961	13.678777595563	14.765344747770
C	23.129208938679	16.443989611026	14.352413322437
C	23.856212744644	17.085488389170	15.511201660828
C	25.910664414385	11.797410864581	15.042232715459
C	23.638057119039	12.488191151939	15.460873946661
C	24.653185440607	11.545210200913	15.591636823013
H	26.710224059906	11.062082299487	15.148868554426
H	22.652861529443	12.319860489583	15.898235092223
H	24.467954708770	10.614761474329	16.131296923284
H	23.791300577166	16.621026930699	16.487483025420
C	26.408939168656	20.752188758290	15.323783559430
C	27.660294478478	20.136237529191	15.190738665189
C	26.092051296039	21.420961429572	16.509741701425
C	24.550948841038	18.260320725551	15.361030916123
C	22.916034985902	21.628580883923	15.153268297195
C	27.010177181308	21.458412337873	17.558955574888
C	28.247964694155	20.832502421246	17.426757757761
C	21.926333682644	22.594197593396	15.320149295910
C	28.572914452322	20.173658418324	16.240612234132
H	27.922264849759	19.621097782145	14.265192533364
H	25.127882162678	21.919817951577	16.620524390349
H	22.915400200300	20.728445364159	15.771558652151
H	26.756946279096	21.980607825562	18.483307966296
H	28.965001771691	20.858805504908	18.249475996229
H	21.156465266100	22.462614244045	16.081902615572
H	29.542400893112	19.683583803541	16.132119028488
H	25.098775551682	18.663632481386	16.215455329586

EET between Tb $^{5}D_{4}$ and linker-centered T1
Electronic energy = -1989.343662389175 (hartree)

Geometry of the high level region. That of the low level region is the same as S0.

O	21.418377109644	14.644518376742	15.711376944465
O	25.912344001267	20.762747568871	12.562446011387
P	25.289675912511	20.535760451834	13.926312745349
P	22.392497414670	14.799666378859	14.562178171574
C	21.626529008269	14.363600283441	12.967201890197
C	20.231536080470	14.281498107745	12.943770193019
C	22.363526116577	14.100594608407	11.808750423804
C	22.861111746919	17.273163859934	13.205480413669
C	25.100080215897	13.979944682076	14.221039533648
C	21.704812145272	13.775040135280	10.626085446313
C	20.311097381606	13.705149066656	10.600234326709
C	26.127657268319	13.048112154707	14.365555974066
C	19.575632934305	13.954569695538	11.757995822339
H	19.676913753202	14.466263822900	13.865744876288
H	23.455110163205	14.143888995743	11.827318461085
H	25.288808857934	14.924238079014	13.703399552088
H	22.282127042242	13.575981906772	9.721089104644
H	19.798252656153	13.449390901963	9.671188028333
H	27.118290834196	13.257413220348	13.960721960444
H	18.486263281603	13.888112378862	11.739502643429
H	22.238150338473	16.924933927062	12.378401059819
C	23.934386359321	21.720524824957	14.223567275074
C	24.570133676985	18.893501755941	14.137079424372
C	23.628850607270	18.380944792220	13.040468937832
C	21.936121706370	23.628036528812	14.585742490007
C	23.891400885730	22.851044944368	13.402352607633
C	22.892472069485	23.804038083034	13.586267181396
H	21.154897444892	24.376993656298	14.730416692727
H	24.642430175315	22.965222731691	12.619274525204
H	22.858006042581	24.686491360172	12.944566308822
H	23.624251134112	18.894183241984	12.086138732370
C	23.836570293613	13.703630884764	14.751056919388
C	23.016057416584	16.468315651543	14.368976767179
C	23.782632098456	17.094527850122	15.535149587824
C	25.894826972323	11.845959160596	15.031713448575
C	23.613718359369	12.509209226207	15.444064815814
C	24.638739355555	11.577468930596	15.576201617348
H	26.702714715426	11.119902888715	15.139050208996
H	22.628997972213	12.328901056617	15.877730204779
H	24.462347134727	10.643589427628	16.112870993085
H	23.585353324803	16.719982883880	16.533441518125
C	26.456884097690	20.676630906937	15.325949762456
C	27.710731856288	20.069255134655	15.178530167266
C	26.146348141849	21.333680819621	16.520366573063
C	24.603748073400	18.172527870225	15.350569472276
C	22.962337074640	21.535056400930	15.211369823865
C	27.072536436647	21.367587074150	17.562445337725
C	28.312446650955	20.749052479223	17.415838338415
C	21.969533290258	22.494279297303	15.397987452905
C	28.631209072872	20.102084646822	16.221720512937
H	27.970101372820	19.564641554148	14.246815235248
H	25.180862808374	21.826955332086	16.643231613365
H	22.983812375968	20.636389765740	15.831248636510
H	26.823912741587	21.881286065511	18.492767320637
H	29.035668388276	20.772135727977	18.233176912050
H	21.217882517385	22.361335039104	16.177186544412
H	29.602046253435	19.617493322243	16.101342116600
H	25.172948368514	18.561261424960	16.198158730679

EET between Eu $^5D_0$ and linker-centered T1

Electronic energy = -1989.343622239615 (hartree)

Geometry of the high level region. That of the low level region is the same as S0.

O	21.541301221170	14.554496363053	15.665629655539
O	25.896208404658	20.715688258950	12.574769270505
P	25.294236880738	20.504013224679	13.949384589719
P	22.525196763118	14.645012932469	14.518445422344
C	21.757188638699	14.207725708861	12.924916378741
C	20.360232415897	14.179641107351	12.886856592012
C	22.495591353573	13.908610674120	11.776046152061
C	23.035363199729	17.089263247667	13.198110513278
C	25.227986649705	13.815905772204	14.223942089169
C	21.837210204081	13.603187954659	10.587623262203
C	20.442308904849	13.587546727976	10.547244235183

C	26.260024374836	12.894544193860	14.397117927941
C	19.704799837418	13.870745572429	11.696124381138
H	19.803439478434	14.393506776331	13.801369370295
H	23.587773691782	13.908221242607	11.807712944092
H	25.421116911065	14.759376576237	13.707050604567
H	22.415620244731	13.378161820383	9.689460346131
H	19.929656099946	13.348001841292	9.613773028188
H	27.257415939578	13.115080642878	14.013515376973
H	18.613933304901	13.846943928986	11.665731783170
H	22.504801636548	16.708941291708	12.320655117751
C	23.965978426847	21.712304183477	14.268850723043
C	24.488918666093	18.881641755872	14.204696038885
C	23.611022950416	18.384290543329	13.135332707659
C	21.997867576553	23.650936336012	14.637082203081
C	23.940274731689	22.845647911302	13.450799650887
C	22.956166032956	23.813554921904	13.637436239478
H	21.227127227645	24.410588756213	14.782235885100
H	24.689913737024	22.949285965787	12.664987699033
H	22.934288222954	24.696849639507	12.996358599537
H	23.525835970407	18.948421082402	12.212442514790
C	23.954142115637	13.534178721602	14.726027604928
C	23.223880138421	16.295290634440	14.296019022182
C	23.835883802891	16.933727488714	15.501483155818
C	26.021390130967	11.695227825899	15.066825885290
C	23.723750320041	12.340973248139	15.417313218431
C	24.753872801329	11.419437807031	15.580855361558
H	26.832136158016	10.976701182092	15.200208615233
H	22.731224087597	12.153873446176	15.829546575176
H	24.571741514881	10.487791246639	16.119495919110
H	23.238810080752	16.868372863414	16.415836938478
C	26.488958514252	20.624654353640	15.327994587743
C	27.752955989618	20.051269244408	15.139407386016
C	26.185917762672	21.235357852530	16.549081896718
C	24.662447628729	18.131265627349	15.318244226003
C	22.993996460684	21.542954061046	15.259136076993
C	27.129350883610	21.256097460596	17.575550629645
C	28.379506930505	20.669964417405	17.387724306346
C	22.015621129512	22.516063959991	15.448267774944
C	28.690782041214	20.069974985758	16.167566851406
H	28.007038733163	19.583136334556	14.187905215699
H	25.213138375653	21.704416741239	16.704329253619
H	23.003206069602	20.642398654309	15.875741172343
H	26.886494920339	21.734346667799	18.526016089193
H	29.116142095935	20.682117482311	18.193166041717
H	21.261448866556	22.390930329641	16.226549288870
H	29.669163423786	19.610235764148	16.015116424053
H	25.346432329466	18.438045284257	16.113927801188

3.5. Model complex with dppcz linker

S0

Electronic energy = -2504.814318180580 (hartree)

Geometry of the high level region.

O	1.922220547154	25.353880489292	12.388657471945
P	2.533447001757	24.026853415579	11.987271952819
C	3.906463314846	23.434627249700	13.034028178311
C	5.094063730500	24.169908384846	12.994196879995
C	6.163134624974	23.788475519270	13.797359455631
C	6.031661985356	22.664002449719	14.643576419883
C	4.852248975590	21.920399519709	14.699600270068
C	3.796076864308	22.322933493716	13.890320630308
H	5.186280127836	25.034551017816	12.335969063387
H	4.762639837918	21.053620344566	15.353401007662
H	2.861509422036	21.760753383893	13.924483752027
C	1.296523890344	22.683140981693	11.986220284685
C	1.509002218401	21.447135742947	11.367094695088
C	0.096783602816	22.924032860273	12.662158875953
C	-0.647300652036	20.686191738545	12.139920984915
C	0.541878849839	20.449170084690	11.448726491279
H	-1.806354988633	22.115157442176	13.269996231334
H	2.429177357272	21.263940891349	10.809576132589
H	-0.071740204494	23.898332892117	13.123884338015
H	-1.405584300627	19.903336446566	12.203384910586

H	0.713924943066	19.484656903067	10.967444824287
C	-0.871413962987	21.924195231512	12.739800835086
C	3.276530364098	24.092681399200	10.318784450364
H	3.126816646078	26.081208776660	7.558918370241
H	2.128826015915	25.847347611106	9.834066799603
H	4.851884020351	24.462578573865	6.789264884283
H	6.660578453814	22.537647851993	17.934914767106
H	5.577554305329	22.610017488160	8.288006450188
H	4.597311435836	22.395976003985	10.552913077729
C	2.872809118780	25.133099517229	9.477622655577
C	4.404704761113	24.354478791919	7.779417882278
C	4.811630834366	23.315052750460	8.616699732015
C	3.437715111092	25.262306250376	8.210238283959
C	4.251999636551	23.187575351589	9.885218450541
N	7.217852746197	22.478832315589	15.337026628139
C	7.464780711838	21.433032868246	16.272326009797
C	7.110247173241	21.598936016678	17.611097641986
C	8.045153803374	20.241769795475	15.835061167689
C	8.258288836160	19.207072347843	16.742847597584
C	7.885169506545	19.360558600433	18.077537858319
C	7.321450009213	20.558489713556	18.513139913280
H	8.317213947192	20.136082383397	14.783784844730
H	8.719912839783	18.279444832448	16.404611382142
H	8.033400805297	18.546403675584	18.787971944948
H	7.038219853432	20.681219154822	19.559970354279
C	8.116934615754	23.466741619871	14.959110693580
C	7.507024911901	24.300586093705	13.993720872877
H	9.884947401660	23.035431913373	16.143772721252
H	11.141671321600	24.956396616902	15.147707200653
C	10.117525597527	24.751578164471	14.840317037176
H	7.783491896999	25.963161663927	12.646229386378
C	9.424749139847	23.682815621556	15.396212967808
C	9.539439080736	25.581570336878	13.862180790550
C	8.232994928777	25.347194061668	13.427707624069
P	10.580130121629	26.911019101439	13.161666867170
O	12.040025614449	26.820207702982	13.561491792210
C	9.817619058250	28.508468724813	13.620035610156
H	10.692867387284	31.802419677841	13.570691739425
H	11.590949255713	29.560782324733	12.977696128732
C	10.088947571986	30.909685375202	13.739576002501
C	10.592037063554	29.655937750436	13.405630088463
H	7.942548674730	27.744983225646	14.368632373431
H	8.421663374696	32.012733174340	14.556344296564
H	7.047486479294	29.975466363162	14.955762400101
C	8.813931063096	31.028267928097	14.294407387555
C	8.043198093867	29.889669754872	14.516747562814
C	8.545866158013	28.632736397956	14.182811553861
C	10.318960894065	26.762410461859	11.356332341928
C	9.390036706329	27.533245990245	10.652405505404
H	11.832913419625	25.228096292638	11.215525347960
H	11.539295560628	24.903980531454	8.773740337173
C	10.929054053302	25.638929390349	9.301927447385
C	11.094121326769	25.818195732704	10.671502285161
H	8.799167899730	28.290271115420	11.172015097608
H	8.495907911344	27.954152843039	8.736239855102
H	9.861859551484	26.260989663335	7.530230048661
C	9.990773735814	26.401991403846	8.604973439210
C	9.223687879548	27.349046986644	9.279745040289

Geometry of the low level region (not optimized)

Tb	1.832053	27.065527	13.636223
Eu	14.190053	27.065527	13.636223
P	5.975054	18.404351	10.335777
O	6.668676	17.156803	9.880374
C	4.637770	18.912855	9.251895
C	7.161142	19.751610	10.410459
C	4.156884	18.892531	12.397171
C	5.219374	18.127871	11.943319
C	8.248969	19.693181	9.553059
C	6.996742	20.861977	11.240158
C	5.009615	16.711598	13.879504
C	3.546288	18.585143	13.598284
C	9.155651	20.751681	9.504749
C	3.969355	17.502509	14.340669

C	5.651760	17.032535	12.689028
H	3.848126	19.628824	11.882598
H	8.375698	18.932331	8.999484
H	6.259710	20.907492	11.836060
H	5.284442	15.949478	14.375683
H	2.829821	19.122861	13.914739
H	9.894815	20.716962	8.910841
H	3.548886	17.300124	15.166822
H	6.382436	16.506249	12.383431
P	2.379126	16.427920	20.050832
F	6.609506	16.201220	17.349568
F	6.774491	14.306937	16.357018
F	7.603444	16.068763	15.446053
O	9.063065	15.996258	18.230671
O	0.936724	16.240566	19.676093
C	8.779483	15.216756	17.298477
C	2.606408	16.402516	21.831892
C	3.355491	15.079179	19.380690
C	3.003591	17.978411	19.406397
C	7.424136	15.447371	16.623045
C	2.455139	19.172822	19.889501
C	4.279231	19.244800	17.813703
C	3.918722	18.026255	18.361295
C	3.736048	20.415078	18.302126
C	2.831940	20.379936	19.340579
H	1.821515	19.150382	20.596208
H	4.903058	19.275285	17.099240
H	4.296545	17.221795	18.023344
H	9.213630	13.676002	16.046654
Eu	11.002969	16.455143	19.580892
F	12.218375	12.057563	17.301709
F	12.775993	13.506695	15.916280
F	11.091899	12.325521	15.481259
F	13.113528	17.529068	15.300274
F	13.155459	19.664864	15.356667
F	11.390757	18.637368	14.731456
O	9.068157	16.851108	20.930835
O	11.719665	17.439921	21.700666
O	11.570536	17.412506	17.432186
O	11.460779	14.540344	18.129819
O	10.719246	18.842182	19.626158
C	10.858189	13.972398	17.203823
C	11.130838	19.811476	18.945051
C	11.870081	18.551450	17.040621
C	11.721072	19.766590	17.691441
C	9.565775	14.211179	16.748045
C	11.691244	12.910995	16.486124
C	12.406384	18.604954	15.602687
H	12.024090	20.567725	17.278885
C	7.917549	21.896766	11.188745
C	8.979709	21.846593	10.317384
H	7.813628	22.647666	11.760714
H	9.594425	22.569337	10.278159
P	-1.880585	27.012920	13.190304
F	0.657439	28.827189	8.613000
F	-0.257112	29.798680	10.160929
F	1.443510	30.593826	9.248792
F	5.814761	28.740180	9.818767
F	6.197127	28.832058	11.898554
F	6.192945	26.777298	15.926493
F	0.542367	22.690429	15.917850
F	5.995773	24.889146	16.924835
F	6.052481	26.961054	10.919713
F	-0.018792	24.151159	17.289601
F	1.647409	22.955054	17.751875
F	0.890357	32.097107	12.854569
F	2.972462	31.818510	12.801605
F	-0.323771	28.179387	17.883953
F	2.089457	32.769678	14.450587
F	5.172726	26.663192	17.817249
F	-0.343381	30.315228	17.817914
F	1.402462	29.272818	18.470770
O	3.788384	27.436108	12.310301
O	1.153332	28.048133	11.502984

O	1.245921	28.037971	15.773141
O	3.749264	26.593119	15.013913
O	-0.438183	26.825566	13.565043
O	1.335847	25.161815	15.089260
O	2.140442	29.449375	13.584544
C	4.012652	25.814910	15.953086
C	1.920417	24.591919	16.025552
C	1.774493	28.720069	10.660211
C	1.729722	30.425736	14.256015
C	4.036436	28.124768	11.301321
C	5.361241	26.034866	16.645388
C	0.952762	29.181575	16.155857
C	1.122599	30.392287	15.501893
C	3.161294	28.811735	10.478049
C	3.209059	24.819708	16.497354
C	1.067299	23.542099	16.736690
C	0.398127	29.246778	17.586334
C	5.544434	28.183833	10.974007
C	0.892631	29.505688	9.688240
C	-1.509093	25.971356	9.315496
C	1.919084	31.789931	13.574572
H	3.987714	21.248329	17.921404
H	2.467673	21.189053	19.678752
H	0.822278	31.198229	15.906992
H	3.505954	29.354322	9.777326
H	3.546509	24.284107	17.205612
C	-2.107867	26.987516	11.409244
C	-2.856950	25.664179	13.860446
C	-2.505050	28.563411	13.834739
C	-3.208344	27.659875	9.387962
P	14.953973	23.935649	11.824980
F	13.015439	28.827189	8.613000
F	12.100888	29.798680	10.160929
F	13.801510	30.593826	9.248792
F	18.172761	28.740180	9.818767
F	18.555127	28.832058	11.898554
F	18.550945	26.777298	15.926493
F	12.900367	22.690429	15.917850
F	18.353773	24.889146	16.924835
F	18.410481	26.961054	10.919713
F	12.339208	24.151159	17.289601
F	14.005409	22.955054	17.751875
F	13.248357	32.097107	12.854569
F	12.005400	21.480239	20.327962
F	15.330462	31.818510	12.801605
F	9.921483	21.222913	20.409578
F	12.034229	28.179387	17.883953
F	14.447457	32.769678	14.450587
F	10.792121	22.158082	18.744943
F	17.530726	26.663192	17.817249
F	12.014619	30.315228	17.817914
F	13.760462	29.272818	18.470770
O	16.146384	27.436108	12.310301
O	13.511332	28.048133	11.502984
O	13.603921	28.037971	15.773141
O	16.107264	26.593119	15.013913
O	14.260352	25.183197	12.280384
O	13.693847	25.161815	15.089260
O	14.498442	29.449375	13.584544
C	16.370652	25.814910	15.953086
C	14.278417	24.591919	16.025552
C	16.291258	23.427145	12.908863
C	13.767886	22.588390	11.750298
C	14.132493	28.720069	10.660211
C	15.709653	24.212129	10.217439
C	14.087722	30.425736	14.256015
C	16.394436	28.124768	11.301321
C	17.719241	26.034866	16.645388
C	13.310762	29.181575	16.155857
C	13.480599	30.392287	15.501893
C	15.519294	28.811735	10.478049
C	15.567059	24.819708	16.497354
C	13.425299	23.542099	16.736690
C	12.756127	29.246778	17.586334

C	17.902434	28.183833	10.974007
C	13.250631	29.505688	9.688240
C	14.277084	31.789931	13.574572
C	10.964211	21.180404	19.622932
H	13.180278	31.198229	15.906992
H	15.863954	29.354322	9.777326
H	15.904509	24.284107	17.205612
H	4.954779	18.833714	8.233002
H	3.789297	18.280793	9.411560
H	4.372038	19.927191	9.464987
H	-1.810523	29.346912	13.614152
H	-3.448232	28.784480	13.380387
H	-2.629378	28.484923	14.894589
H	-2.317892	25.194994	14.656801
H	-3.784049	26.046213	14.233861
H	-3.050970	24.947278	13.090181
H	-1.286386	26.476542	10.952108
H	-3.019636	26.479696	11.173244
H	-2.153011	27.991173	11.041095
H	17.139730	24.059207	12.749197
H	15.974249	23.506286	13.927755
H	16.556990	22.412809	12.695770
H	16.652296	23.707630	10.174937
H	15.066870	23.832129	9.451065
H	15.859583	25.261192	10.069491
H	14.188590	21.717086	12.207177
H	12.876229	22.868911	12.271025
H	13.532409	22.376751	10.728212
H	3.518177	16.903399	22.082280
H	1.784927	16.900245	22.303416
H	2.651552	15.388767	22.171266
H	4.282590	15.012329	19.910709
H	2.816433	14.161110	19.487768
H	3.549512	15.260046	18.344088
C	8.834330	17.387068	22.033367
C	11.156971	17.918733	22.706458
C	9.785799	17.867749	22.926974
H	9.430097	18.230793	23.868555
C	7.356409	17.529353	22.442150
C	12.035186	18.607752	23.767398
F	12.959347	19.378241	23.155211
F	12.654934	17.670797	24.516081
F	11.261138	19.378229	24.560945
F	7.278710	18.137956	23.644675
F	6.791155	16.305841	22.519663
F	6.703781	18.268992	21.520460

Linker-centered T1
Electronic energy = -2504.690432468605 (hartree)
Geometry of the high level region. That of the low level region is the same as S0.

O	1.919391708606	25.356354443697	12.385299244003
P	2.512415072249	24.020376499771	11.987145819623
C	3.894078788654	23.430469973371	13.025004753496
C	5.087814999507	24.199832537182	12.981741241000
C	6.213411647270	23.784555820547	13.831709351822
C	6.058656020455	22.641329621266	14.673243686380
C	4.910814998524	21.896964012520	14.717414604379
C	3.788049879952	22.333658769485	13.847915881033
H	5.170102177156	25.087231350923	12.358545231297
H	4.810438893719	21.012550749224	15.342544345559
H	2.860712259663	21.761106274441	13.886797989473
C	1.265554957919	22.687196614638	12.005030383089
C	1.465451493341	21.444031326517	11.395772418054
C	0.069764021364	22.944961900242	12.681682063058
C	-0.695677328579	20.709536886533	12.180792410135
C	0.489446116728	20.455716746627	11.488535874823
H	-1.838845724835	22.158574801828	13.301617792559
H	2.382414891578	21.247814300789	10.837545275116
H	-0.089055764440	23.924510597184	13.135423192243
H	-1.460680813098	19.933989608521	12.253084059952
H	0.651447482724	19.485633515283	11.015028541008
C	-0.907214146561	21.954632803828	12.770463093404
C	3.254971942171	24.067435156406	10.319015211938
H	3.136871217355	26.037424998783	7.544926499263

H	2.127449849211	25.830833372746	9.818109384603
H	4.851239323719	24.398364107288	6.796240706406
H	6.663136673191	22.522656324545	17.954126146304
H	5.555219840342	22.551754030576	8.312211650414
H	4.559324671185	22.361380089204	10.574241437104
C	2.865315393417	25.106259624169	9.469702285129
C	4.398324851065	24.301313263200	7.784911054615
C	4.792932926384	23.264816828467	8.631837064685
C	3.437235784140	25.220831328065	8.204016135601
C	4.225584342292	23.151137566464	9.898186447389
N	7.256121848991	22.456838977271	15.366631255169
C	7.492213002912	21.413293573705	16.307560655424
C	7.116878059354	21.582734829987	17.639763071999
C	8.075799118339	20.221289655135	15.878599797057
C	8.272140744305	19.187112597172	16.790500513448
C	7.879360409165	19.342972471774	18.119237276501
C	7.312530521948	20.542782207771	18.545652855490
H	8.362248633381	20.114144858362	14.831449275283
H	8.735924712119	18.258086274913	16.459461776054
H	8.014700327337	18.529111752799	18.832489353396
H	7.013752837545	20.666893024419	19.587961057142
C	8.149724804550	23.433399401081	14.994688108125
C	7.497421742915	24.271993325136	14.006694914843
H	9.919555216988	23.030951234495	16.169694157424
H	11.150193535499	24.970970993110	15.156400269243
C	10.128716310587	24.749253616040	14.854580344928
H	7.794287799111	25.941386593852	12.629571012830
C	9.447503133062	23.668211865974	15.421181396911
C	9.534388748293	25.570954664480	13.862406946217
C	8.242590554078	25.340934490026	13.422308953368
P	10.576301691510	26.905959531373	13.160530185125
O	12.037084290996	26.817650183712	13.559002519828
C	9.810670133810	28.499777986554	13.626433821086
H	10.711542293500	31.787059688057	13.637779307738
H	11.601995716058	29.546280452671	13.028400645636
C	10.097557374932	30.897072862223	13.783123269045
C	10.596980627800	29.644058950480	13.440844930831
H	7.914803620942	27.740534598666	14.325614754327
H	8.424837758754	32.001389713137	14.586822272808
H	7.027531245328	29.969697274174	14.930540313167
C	8.814076630814	31.017760485086	14.317286357678
C	8.030538188182	29.882189714832	14.508741390138
C	8.529148632297	28.625981884111	14.166399492046
C	10.315637976175	26.758848090448	11.355800026974
C	9.388491623527	27.532273378839	10.652586530744
H	11.831240387556	25.226112679175	11.213673337199
H	11.542901030295	24.909665710126	8.770217866254
C	10.931187613555	25.642609393646	9.299460428406
C	11.093103834879	25.817328874846	10.669983594468
H	8.796455867296	28.287382974005	11.173554913712
H	8.499301010243	27.959700969631	8.735665146823
H	9.868172466611	26.270545717224	7.527418636629
C	9.994454734081	26.407920363215	8.602940249939
C	9.225637051424	27.352703273015	9.278963892538

ISC between the ground state and linker-centered T1
Electronic energy = -2504.669514551686 (hartree)
Geometry of the high level region. That of the low level region is the same as S0.

O	1.940602242543	25.351068314292	12.395019462799
P	2.541412161155	24.023637195958	11.978670401528
C	3.916918470172	23.411588251573	13.013028503334
C	5.071448413933	24.191220757674	13.037879294555
C	6.149666163166	23.795920569661	13.832429410557
C	6.044625988090	22.598201088341	14.584967177664
C	4.896172932079	21.804705785452	14.566796049242
C	3.833098256367	22.232572069462	13.784261596696
H	5.134680375490	25.108849168996	12.452011118996
H	4.831809013377	20.892503951323	15.160095885291
H	2.912524659268	21.648142166704	13.772092120287
C	1.293618433640	22.689971990915	11.966519376324
C	1.494011383270	21.457784634606	11.335634063549
C	0.096482742552	22.935940574981	12.645166667123
C	-0.669803594266	20.710869323042	12.101650662030
C	0.517146649771	20.468578690759	11.408429543658

H	-1.814607156767	22.140432611555	13.245503595175
H	2.413577103272	21.269630236910	10.778449416175
H	-0.062399151479	23.907402493825	13.116079235801
H	-1.435709510846	19.934917736572	12.157688537799
H	0.680045928195	19.506636199760	10.918900534173
C	-0.881705050629	21.945274159652	12.713213629443
C	3.277945637253	24.097994490261	10.307767633408
H	3.109915635535	26.085257004339	7.548334634630
H	2.119595972279	25.846714021820	9.826175735254
H	4.840207866044	24.474694217222	6.773552194923
H	6.651358493182	22.506554076006	17.903840576225
H	5.580706243755	22.626863237665	8.270938336223
H	4.609557702016	22.409995609065	10.540309683199
C	2.866512247530	25.136378311809	9.467857331545
C	4.396996892459	24.364484206100	7.765303477513
C	4.812378564871	23.328078783111	8.602068887073
C	3.426777929998	25.267796791478	8.198640563976
C	4.257408801069	23.198422829663	9.872403502819
N	7.214109147148	22.437699096738	15.313419389185
C	7.466125445488	21.393002548801	16.251843358422
C	7.109451552330	21.568914785793	17.588401494773
C	8.047067344559	20.201749551017	15.820272865107
C	8.263055035876	19.174818587701	16.736227935899
C	7.892906418621	19.337842810778	18.070415837822
C	7.326925331080	20.537374408679	18.498446732721
H	8.319919588444	20.090352396513	14.770000192913
H	8.728609630372	18.246972351271	16.404831674529
H	8.044857032190	18.529913098477	18.786975573148
H	7.044540801204	20.667316269322	19.544568665652
C	8.029411789656	23.509729241526	15.046883907376
C	7.437600920613	24.365320896454	14.138894244699
H	8.881680522469	24.708300597991	16.597213946496
H	11.219194539382	24.628516266104	14.837636120838
C	10.141474645030	24.781697256620	14.844558757781
H	7.668952390292	26.210649225933	12.971215046536
C	9.209956767079	24.063163033749	15.756409589002
C	9.521979428190	25.703753567311	14.011780165282
C	8.148030718806	25.543467631611	13.689723066476
P	10.582156080356	26.957659574512	13.203119830536
O	12.049192310053	26.815836676397	13.552737913171
C	9.873809987011	28.588002956364	13.642139481751
H	10.723904783567	31.871526173908	13.294020261201
H	11.581547587136	29.595942204900	12.781561786710
C	10.145896088392	30.992032054563	13.580184755458
C	10.622203559841	29.717806186437	13.286336866618
H	8.083756789401	27.884801998849	14.623206615075
H	8.555607118940	32.152814738029	14.466437733206
H	7.238028649691	30.148287678110	15.137772776103
C	8.927086992471	31.151594272253	14.240216252547
C	8.188598954044	30.032238749592	14.614087181094
C	8.663092234351	28.755148299118	14.318507218661
C	10.268297528872	26.766328279117	11.411474824520
C	9.309749755432	27.508590246024	10.717361226337
H	11.805266957466	25.256653012155	11.261259609246
H	11.445443166822	24.870404859060	8.836283459163
C	10.838557914094	25.607210048108	9.365733691599
C	11.039305362367	25.819479078020	10.725969239513
H	8.725906257793	28.270820786106	11.237009794153
H	8.358636469674	27.876282829758	8.817811440673
H	9.713970151089	26.175711785244	7.611506020877
C	9.870071622529	26.341006250713	8.679171621525
C	9.108685162920	27.292632088438	9.354266706690

EET between Tb $^5D_4$ and linker-centered T1
Electronic energy = -2504.689281714510 (hartree)
Geometry of the high level region. That of the low level region is the same as S0.

O	1.879947007064	25.359977359837	12.378384857970
P	2.473256803669	24.019680315484	11.995904144745
C	3.838911816075	23.428899904545	13.056067777066
C	5.018891351377	24.156997717766	13.000615263245
C	6.104428755125	23.768742733410	13.805580860773
C	5.953937348525	22.636650253503	14.662581192820
C	4.765219406410	21.907472817341	14.730441745043
C	3.712521500828	22.315932157577	13.924469666520

H	5.118001357794	25.014293253309	12.334002415229
H	4.670325668305	21.043281774714	15.386668672835
H	2.771274799212	21.766326026276	13.960938315824
C	1.221442800880	22.690077377715	12.010473992032
C	1.424692441957	21.441749834758	11.413173213242
C	0.022110696523	22.953491590785	12.678490495393
C	-0.742158832999	20.714969220260	12.189418859872
C	0.447328174596	20.454942026845	11.507009152853
H	-1.890636643670	22.172778792842	13.293211098174
H	2.345802282056	21.239504401628	10.863742911223
H	-0.137437077274	23.936275250082	13.125183411419
H	-1.508267690435	19.940583387351	12.262617934389
H	0.611644455934	19.481094736099	11.042053822445
C	-0.956120472041	21.964500924245	12.768809484779
C	3.219813787886	24.054420516969	10.328056115420
H	3.094330562307	26.013694676331	7.546238571542
H	2.093753443336	25.817753365643	9.824154489728
H	4.800705859247	24.366785939516	6.795876269324
H	6.620078574077	22.505400474135	17.914888470389
H	5.505996307926	22.524175018901	8.316159343788
H	4.523500966351	22.347460025286	10.583903446398
C	2.828595481836	25.090203227964	9.475443610873
C	4.351898216617	24.274907956921	7.786924671304
C	4.747265378968	23.240806904783	8.636442825876
C	3.395307800004	25.198574514183	8.206872077409
C	4.185856069069	23.133857534917	9.906046022322
N	7.151068391171	22.445024526483	15.316550971284
C	7.421922046828	21.401689116925	16.250908280760
C	7.079717825035	21.571359616467	17.591795215656
C	8.015321201214	20.219130382642	15.810160021304
C	8.255607896928	19.193408072104	16.721319662043
C	7.895042572754	19.347847601389	18.059309576365
C	7.317709301973	20.538784209446	18.495987524871
H	8.276173352747	20.112975655140	14.756219379515
H	8.728627623868	18.271788517587	16.382713796339
H	8.063684131398	18.539740077440	18.771847838059
H	7.045189773376	20.662681222177	19.545449693047
C	8.048523699383	23.411349354809	14.891271115479
C	7.428842857224	24.238658254765	13.962204461792
H	9.838245570076	22.979871994752	16.100552114014
H	11.142950746402	24.912822911380	15.063286464623
C	10.124285499198	24.718400276819	14.728532578157
H	7.989005287791	25.511274977256	12.230983232123
C	9.393428865647	23.592295454685	15.317176369491
C	9.568484638479	25.538828188232	13.808888405818
C	8.198389014346	25.287817219092	13.281158525710
P	10.591653699903	26.890729057092	13.145732589521
O	12.045068094417	26.841477836542	13.571703161366
C	9.768897528019	28.452879403854	13.603512164301
H	10.612367491166	31.743611923799	13.868016641263
H	11.579091786368	29.549775550729	13.195546783761
C	10.002792187435	30.839472516985	13.916533190702
C	10.546101488554	29.614931075316	13.538672436766
H	7.835783024208	27.622675723125	14.100943010889
H	8.258493644451	31.868516132337	14.666160638238
H	6.874134248886	29.804003733062	14.783953653378
C	8.683413035383	30.908898073217	14.365513852318
C	7.906516615147	29.753381427203	14.432743725989
C	8.448059744584	28.526195379908	14.053415062026
C	10.359206424586	26.718916636388	11.337361492572
C	9.454661988279	27.495159644088	10.607797239385
H	11.838095650142	25.147528199299	11.245554073589
H	11.579272695106	24.793089061756	8.804141582292
C	10.977741609076	25.550277698012	9.310495491175
C	11.124157038825	25.747584274364	10.679888781747
H	8.863619931826	28.264757701131	11.108357416454
H	8.600940033406	27.904694722465	8.671187468010
H	9.953356109723	26.168328372044	7.512492661985
C	10.067973120134	26.322754327514	8.587016837478
C	9.308546558742	27.294562712023	9.235362403782

EET between Eu $^5D_0$ and linker-centered T1
Electronic energy = -2504.688222256850 (hartree)
Geometry of the high level region. That of the low level region is the same as S0.

O	1.865931556613	25.365580434424	12.371391408347
P	2.460077511302	24.022930021504	11.999298438949
C	3.824414024639	23.440803167632	13.064324658295
C	5.006328093842	24.167255040196	13.003200081440
C	6.093048445096	23.779087868170	13.803041018831
C	5.947215683118	22.650024237869	14.661314335648
C	4.755710731174	21.925923304912	14.740643874018
C	3.700154916800	22.333442253893	13.938243244488
H	5.107244048300	25.019569183452	12.330397380968
H	4.662930401218	21.062719620072	15.398489911791
H	2.758827305350	21.784163467723	13.980446185721
C	1.208959658210	22.692580011960	12.021885110923
C	1.412455592148	21.441048345732	11.431408166919
C	0.009912465237	22.958919676910	12.689266618116
C	-0.753316365955	20.717190581173	12.213359801330
C	0.435775911667	20.454163507848	11.531427668789
H	-1.901765956728	22.180394267193	13.310050432471
H	2.333149701781	21.236382935742	10.882221794468
H	-0.149927917879	23.944054392951	13.130604031613
H	-1.518894409049	19.942740987848	12.291489586642
H	0.600310595032	19.477916479156	11.071600589872
C	-0.967585914424	21.969800564762	12.785965730984
C	3.208353338314	24.046387576700	10.331914447882
H	3.089038427375	25.989417005465	7.538437277797
H	2.086406611040	25.809112430332	9.817009156199
H	4.792834275299	24.334955683295	6.799377061346
H	6.620772835785	22.495055610528	17.890420970225
H	5.493502356732	22.500093497351	8.330975801558
H	4.508705855556	22.338711714412	10.599249486074
C	2.819958148766	25.077997981505	9.472988503849
C	4.343037439905	24.249777413151	7.790569776202
C	4.735909331658	23.220025982354	8.646553258983
C	3.387897438100	25.177737390338	8.204237808177
C	4.173197901398	23.121648333541	9.916284992476
N	7.154160317772	22.442519870490	15.293811199356
C	7.428073722105	21.397641785843	16.225007562675
C	7.083761403156	21.563081369122	17.566090417154
C	8.026045063064	20.217630247539	15.783281554082
C	8.269426233124	19.191287851768	16.693077988451
C	7.906514852283	19.341896016078	18.030853693247
C	7.324017038303	20.529836186400	18.468796098454
H	8.287783434367	20.113605597951	14.729287365153
H	8.745853841660	18.271735560657	16.353495631038
H	8.077249731580	18.533368089699	18.742372463663
H	7.049624280092	20.650902000567	19.518125707338
C	8.055507494097	23.395475146281	14.846627740620
C	7.427567476387	24.236512171171	13.937916500799
H	9.864195788379	22.927605325762	16.012393096881
H	11.163820515152	24.859846231719	15.004744649629
C	10.146726519435	24.666987194874	14.663263042987
H	8.147862697699	25.221112428638	12.098653869029
C	9.418173765051	23.550216510103	15.237215291929
C	9.585790156066	25.512898889560	13.765021102193
C	8.225852567797	25.228473234223	13.194359539189
P	10.593000208477	26.891162288620	13.137247561059
O	12.044849658930	26.848897045549	13.569595215633
C	9.746218316167	28.430163893092	13.623078964851
H	10.542403226488	31.721653799577	13.994858641189
H	11.547756564585	29.559597255347	13.273774496670
C	9.944137956161	30.808650564122	14.008270367418
C	10.509130267873	29.602757613928	13.603051468059
H	7.817175378370	27.558801645380	14.061982510053
H	8.175341507697	31.791434462007	14.762021700872
H	6.816081564944	29.707676109524	14.796634699298
C	8.617422062843	30.846898751249	14.439084538776
C	7.854411803416	29.680132257130	14.460647541653
C	8.417645948106	28.471756631152	14.053589556384
C	10.370982301710	26.751544468175	11.324277735456
C	9.449237767636	27.521376549690	10.609794270292
H	11.882107368681	25.212974573060	11.201997513204
H	11.632302545577	24.902472167377	8.754184230491
C	11.014244745379	25.636143844256	9.275034208497
C	11.156045661342	25.809493288003	10.648361006801
H	8.840192112726	28.266300455445	11.125416549789

H	8.586886082498	27.950466012164	8.681544822082
H	9.977492031417	26.267496567396	7.488930739591
C	10.088513915017	26.403198077671	8.566352264236
C	9.307931544351	27.345070119081	9.233652695889

3.6. Model complex with dpbt linker

S0

Electronic energy = -2861.423936552327 (hartree)

Geometry of the high level region.

S	23.581310489707	16.948925232033	14.737343544665
C	24.209741666838	18.141842470067	13.649161619323
C	24.680866313263	17.436693934126	15.991691091502
C	25.184357446333	18.899006018307	14.249605339654
H	25.694677412647	19.721016027539	13.747098875368
C	25.452301749090	18.499649141082	15.584795206134
H	26.193580898639	18.979652908253	16.221844081867
P	23.639742116331	18.155935123636	11.929503663495
O	22.183169112957	17.798473458778	11.756737651546
C	24.720163411823	16.956730684702	11.080875405649
C	26.104469968017	16.872731040017	11.262628067550
C	24.067158852704	16.034443067334	10.258091717229
C	26.832569208025	15.890759591131	10.595449969357
C	24.797190134976	15.041765081141	9.607667207680
C	26.179374927312	14.973469991433	9.771984251571
H	26.615892397228	17.552882522940	11.948235073773
H	22.982504782239	16.099582029768	10.161233009027
H	27.913261029459	15.827713317072	10.731641346843
H	26.752841883666	14.194397028808	9.266167350591
H	24.283438403788	14.314085178284	8.977362633052
C	24.386347954836	22.507617566240	10.619764665907
C	22.953443670882	20.753063773469	11.451419910079
C	24.019613393729	19.849460324333	11.375314104584
C	25.266703006694	20.277644348696	10.912426177193
H	24.529677917562	23.547827973208	10.322441402250
C	25.448743694055	21.608196933035	10.539152924764
C	23.139715640234	22.080328150632	11.076306504317
H	26.096199684066	19.573409852070	10.825049234123
H	21.980231169807	20.397746573152	11.796155116431
H	22.306453142610	22.782815025428	11.134092618827
H	26.422360038210	21.940789472691	10.174982542481
S	25.756814950908	17.285407742270	18.548913553805
C	25.174182441546	16.055706197782	19.619693758799
C	24.705680740215	16.745487258462	17.273388333016
C	24.244248022561	15.255708733781	19.004300398340
C	23.976485055020	15.647168196534	17.665930213126
H	23.771165860796	14.404530503666	19.494848849666
H	23.274827223681	15.128175514189	17.012942864332
P	25.760282875048	16.063830313022	21.333662013912
O	27.199512059329	16.501029733874	21.477472759484
C	24.629422410089	17.202215575117	22.199838190737
C	25.234327746026	18.133114255344	23.049178573084
C	23.244407692463	17.230900699564	22.006277574547
C	22.468019373269	18.164852731533	22.687525058947
C	24.455616970048	19.077233839947	23.715307768338
C	23.073171906543	19.089682204260	23.538842578741
H	22.770284715398	16.541471685052	21.303176312211
H	26.319507238856	18.113610310452	23.158024583498
H	21.386551151863	18.181698208704	22.543721987659
H	22.461447542607	19.828440426490	24.060017915342
H	24.931076765382	19.808825654035	24.370802303219
H	27.207866985395	11.435476498948	21.967223256883
C	25.142240201241	11.664174360371	22.556212474664
H	25.019128845722	10.611869558983	22.818618499165
H	23.115371277589	12.187294845176	23.084164905901
C	24.072629464781	12.546267587657	22.701944007457
C	26.368310731317	12.124490281431	22.076248839576
C	26.525374952210	13.465721980122	21.738930766408
H	23.390417462458	14.579343600528	22.505011046616
H	27.482720483542	13.839272592086	21.371319009250
C	24.226187013805	13.890934417916	22.368226157662
C	25.450982962817	14.352100947032	21.877312445812

Geometry of the low level region (not optimized)

Tb	29.368537	17.317346	20.806910
Eu	19.899722	16.777904	12.484146
F	16.516087	13.014839	12.805072
F	17.651840	11.491327	11.899888
F	16.931955	13.122443	10.777647
C	18.715117	13.610824	12.234630
C	17.457227	12.785719	11.931515
F	28.847870	13.349245	16.620227
P	27.740538	11.113142	12.994997
F	23.283358	13.014839	12.163220
F	22.147604	11.491327	13.068404
F	22.867490	13.122443	14.190646
O	29.154522	10.631172	13.057584
C	26.671999	10.067782	13.988902
C	27.120223	11.117779	11.325784
C	21.084328	13.610824	12.733663
C	27.574194	12.808494	13.533147
C	19.899722	12.927555	12.484146
C	26.491751	13.239867	14.291517
C	22.342217	12.785719	13.036778
H	29.305712	13.396706	12.632291
H	19.899722	11.976979	12.484146
H	25.826839	12.622062	14.571495
P	27.740538	24.751242	12.994997
F	20.234678	21.259207	14.603122
F	30.848410	19.009329	16.369212
F	21.379595	15.085921	8.046448
F	22.089597	20.493836	15.355500
F	28.773317	19.112570	15.998516
F	29.954461	17.597650	15.065535
O	20.420512	18.681879	13.871273
O	29.154522	24.269272	13.057584
O	19.793548	16.129644	14.800205
O	21.279198	14.834025	12.742318
C	26.671999	23.705882	13.988902
C	19.769018	16.730403	15.883995
C	27.120223	24.755879	11.325784
C	20.764294	20.378595	15.440558
C	25.300678	23.641646	13.808797
C	20.350270	18.945094	15.079850
C	28.558928	13.700017	13.134320
C	27.574194	26.446594	13.533147
C	29.779447	18.269599	16.193103
C	27.342063	15.468333	14.211952
C	27.263480	22.937920	14.975649
C	20.001116	18.086439	16.125855
C	24.523736	22.852000	14.649230
C	25.110547	22.134636	15.648294
C	28.441792	15.041460	13.476386
C	26.469348	22.151002	15.811587
H	24.890988	24.139437	13.110018
H	27.248756	16.388905	14.423350
H	28.207933	22.946103	15.084178
H	29.348139	15.667449	16.282656
H	23.583694	22.812450	14.526552
H	24.571576	21.619116	16.236048
H	25.660600	14.876439	15.162412
H	29.108372	15.661994	13.209891
H	26.872556	21.625935	16.494054
P	16.347538	17.932192	12.994997
F	19.379055	20.746005	8.297463
F	19.564767	21.259207	10.365170
F	17.709848	20.493836	9.612792
F	19.304502	14.982680	7.675752
F	20.485646	16.497600	6.742771
O	19.378933	18.681879	11.097019
O	17.761522	17.450222	13.057584
O	20.005896	16.129644	10.168087
O	18.520247	14.834025	12.225974
C	26.391364	14.576401	14.633084
C	15.278999	16.886832	13.988902
C	20.030427	16.730403	9.084297
C	15.727223	17.936829	11.325784

C	19.035151	20.378595	9.527734
C	19.449175	18.945094	9.888442
C	16.181194	19.627544	13.533147
C	20.310633	15.825651	7.870339
C	19.798328	18.086439	8.842437
H	19.879325	18.427801	7.959892
F	29.033582	12.836043	18.687934
F	27.178662	13.601414	17.935556
P	21.527722	9.344008	20.296059
F	29.889205	13.349245	24.993593
F	29.703493	12.836043	22.925886
F	31.558412	13.601414	23.678264
O	20.113737	9.825978	20.233472
C	22.596261	10.389368	19.302154
C	22.148037	9.339371	21.965272
C	23.967581	10.453604	19.482259
C	22.004780	11.157330	18.315407
C	24.744523	11.243250	18.641826
C	24.157713	11.960614	17.642762
C	22.798912	11.944248	17.479469
H	21.060327	11.149147	18.206879
H	25.684566	11.282800	18.764504
H	24.696683	12.476134	17.055008
H	22.395703	12.469315	16.797002
P	32.920722	16.163058	20.296059
F	27.888665	19.009329	25.244608
F	29.963758	19.112570	25.615304
F	28.782614	17.597650	26.548286
F	32.752172	21.080411	20.485984
F	31.616419	22.603923	21.391168
F	32.336304	20.972807	22.513410
O	28.847748	15.413371	19.419783
O	31.506737	16.645028	20.233472
O	29.262363	17.965606	23.122969
O	30.748013	19.261225	21.065082
C	33.989261	17.208418	19.302154
C	29.237833	17.364847	24.206759
C	33.541037	16.158421	21.965272
C	28.503965	13.716655	17.850498
C	28.917990	15.150156	18.211206
C	30.553143	20.484426	21.056427
C	33.087066	14.467706	19.757909
C	28.957627	18.269599	25.420718
C	29.368537	21.167695	20.806910
C	29.469931	16.008811	24.448619
C	31.811032	21.309531	21.359542
H	29.368537	22.118271	20.806910
H	29.388935	15.667449	25.331165
P	21.527722	22.982108	20.296059
F	20.420390	20.746005	16.670829
F	20.494943	14.982680	17.292540
F	19.313799	16.497600	18.225522
F	25.984902	21.080411	21.127836
F	27.120655	22.603923	20.222652
F	26.400770	20.972807	19.100411
O	29.889327	15.413371	22.194037
O	20.113737	23.464078	20.233472
O	29.474711	17.965606	18.490851
O	27.989062	19.261225	20.548738
C	22.596261	24.027468	19.302154
C	29.499241	17.364847	17.407061
C	22.148037	22.977471	21.965272
C	30.233109	13.716655	23.763322
C	29.819085	15.150156	23.402614
C	20.709331	20.395233	20.156736
C	28.183932	20.484426	20.557394
C	21.694066	21.286756	19.757909
C	19.488812	15.825651	17.097953
C	21.926196	18.626917	19.079104
C	29.267143	16.008811	17.165201
C	22.776508	20.855383	18.999539
C	22.876896	19.518849	18.657972
C	26.926042	21.309531	20.254279
C	20.826468	19.053790	19.814670

C	21.600727	7.679836	19.654904
H	19.962548	20.698544	20.658765
H	22.019503	17.706345	18.867706
H	19.920120	18.427801	17.008401
H	23.441421	21.473188	18.719561
H	23.607660	19.218811	18.128645
H	20.159887	18.433256	20.081165
F	18.419850	15.085921	16.921844
H	34.903580	16.691679	19.097460
H	33.499554	17.445447	18.380794
H	34.201567	18.111232	19.835777
H	34.484211	16.662738	21.996670
H	32.846348	16.661048	22.605330
H	33.665280	15.148690	22.296796
H	33.990132	14.054160	20.155843
H	32.250420	13.899089	20.106620
H	33.120276	14.434446	18.688942
H	17.017839	20.196161	13.184437
H	15.278128	20.041090	13.135213
H	16.147983	19.660804	14.602114
H	16.421912	17.434202	10.685726
H	14.784048	17.432512	11.294386
H	15.602980	18.946560	10.994260
H	15.768706	16.649803	14.910262
H	14.364679	17.403571	14.193596
H	15.066693	15.984018	13.455279
H	23.510580	23.510729	19.097460
H	22.106554	24.264497	18.380794
H	22.808567	24.930282	19.835777
H	23.091211	23.481788	21.996670
H	21.453348	23.480098	22.605330
H	22.272280	21.967740	22.296796
H	27.814912	10.615152	10.685726
H	26.177048	10.613462	11.294386
H	26.995980	12.127510	10.994260
H	27.161706	9.830753	14.910262
H	25.757679	10.584521	14.193596
H	26.459693	9.164968	13.455279
H	24.444637	9.869624	20.259341

Linker-centered T1

Electronic energy = -2861.343085130246 (hartree)

Geometry of the high level region. That of the low level region is the same as S0.

S	23.488513390812	16.921650491152	14.756864903592
C	24.157223818059	18.116723442143	13.668171186946
C	24.629463793453	17.413649745439	16.039812698791
C	25.142128423612	18.911933918524	14.292802191331
H	25.634124913044	19.734822490341	13.772655405002
C	25.413023687661	18.554995657050	15.589054537120
H	26.140637849299	19.048707903841	16.230586830834
P	23.651928671057	18.100031046790	11.933502025908
O	22.221667679829	17.664187610666	11.722610647735
C	24.822705540496	16.964646881337	11.117150696808
C	26.209918027042	17.006547062037	11.293737107413
C	24.249309075255	15.962576428576	10.328898982451
C	27.019658119471	16.074796161703	10.649901747353
C	25.062228595784	15.015760586791	9.708492576881
C	26.445973097299	15.076414973415	9.862403172024
H	26.663394806323	17.747946146001	11.956332868986
H	23.162772812466	15.930719481131	10.232630022410
H	28.102444070796	16.115150816456	10.778314513112
H	27.083853393959	14.335380727044	9.377114211135
H	24.612601548071	14.223590746126	9.107531269981
C	24.212725153745	22.505700338754	10.700620742274
C	22.868985856773	20.684456555476	11.538204010088
C	23.959937118692	19.817129091016	11.401303695572
C	25.173003165802	20.298212169836	10.901651494535
H	24.313843967135	23.557654532647	10.427943183612
C	25.298181544543	21.643109953986	10.556432306389
C	22.998252582971	22.025893470059	11.191174116146
H	26.020756131242	19.625480152088	10.762201707206
H	21.919737671164	20.291557568178	11.907915130339
H	22.145251838726	22.698301308341	11.298013473488
H	26.246033701048	22.015557124755	10.164065733260

S	25.850320970472	17.271422123364	18.510624438813
C	25.204532964195	16.061541214929	19.594367301858
C	24.722609788206	16.756754468278	17.223269309788
C	24.232558767105	15.250953643684	18.970695718869
C	23.953738268878	15.606083064924	17.674407947665
H	23.758960053323	14.414879406763	19.487106316633
H	23.242648634640	15.093213833912	17.027998019981
P	25.757921862762	16.080253225960	21.313817199570
O	27.194817751748	16.520755652626	21.472299640351
C	24.616642996842	17.216700352016	22.169466235677
C	25.213160038037	18.168432706241	23.001610872823
C	23.229808424012	17.220566630202	21.986325375925
C	22.443258367350	18.148849316045	22.663584037687
C	24.423914967459	19.107611263171	23.662311370545
C	23.039895151674	19.093857868779	23.498496945325
H	22.759828048797	16.514812780914	21.296569372458
H	26.299327503383	18.166740066211	23.103482696385
H	21.360356657658	18.144484777227	22.530103375235
H	22.419977468742	19.827432692953	24.017256459731
H	24.892220061480	19.855016107644	24.304993202259
H	27.213527583839	11.454037385625	21.937147474881
C	25.151183403984	11.677871501810	22.539489155950
H	25.032084088749	10.625377854410	22.802944366297
H	23.127058075738	12.196996211962	23.081384545579
C	24.080858056159	12.557775127237	22.692296094839
C	26.372976875056	12.140979072009	22.051413175766
C	26.524810209612	13.482537174705	21.713078359682
H	23.392823082896	14.588721940005	22.501627960785
H	27.479700880292	13.857830304551	21.341003423250
C	24.229262619220	13.902827916275	22.357417166170
C	25.449442813818	14.367191254193	21.857942425274

ISC between the ground state and linker-centered T1

Electronic energy = -2861.314857572385 (hartree)

Geometry of the high level region. That of the low level region is the same as S0.

S	23.318816866148	17.182755486366	14.774206036957
C	24.607295577564	17.788397880192	13.709065406358
C	24.531670856023	17.486874053212	16.107529427369
C	25.421537914244	18.853673302388	14.409860127443
H	25.938384562952	19.645596028452	13.865073208325
C	25.367019846459	18.719699913093	15.719199344091
H	25.819577659443	19.365366802719	16.470312564413
P	24.488740746283	17.537351400519	11.917750537489
O	23.332914573463	16.643584469638	11.536439480137
C	26.090660856688	16.958301746132	11.278378887839
C	27.311069556913	17.612612693243	11.488715303051
C	26.051028496821	15.785476276004	10.518915981284
C	28.473645895074	17.119298426552	10.902651231931
C	27.221910448286	15.280682520730	9.956647629198
C	28.428818800710	15.953536135890	10.136259724181
H	27.366021896360	18.503732252473	12.117515208012
H	25.092198294006	15.281979758355	10.382631140389
H	29.422874341445	17.632547276540	11.064539513111
H	29.344146385498	15.560571669532	9.690271518945
H	27.190381205869	14.357954063774	9.374953573782
C	23.762510147038	22.011249898726	10.989629973924
C	23.038821318420	19.856215404535	11.793659869819
C	24.274147727883	19.297534590500	11.437249211077
C	25.239493883278	20.103665650346	10.827212599870
H	23.569339928907	23.072750843423	10.825274810360
C	24.982032220830	21.457055626414	10.606648902197
C	22.786165066648	21.206436608531	11.577087098837
H	26.193241581015	19.681958851139	10.508804117903
H	22.274572657164	19.219349461184	12.238435967430
H	21.825324404453	21.631973859749	11.871396157048
H	25.741524885438	22.080834382663	10.132236483906
S	25.941516038599	17.150999601846	18.430086661648
C	25.289279791100	15.940485898115	19.510163357827
C	24.748261064739	16.699712758491	17.157243591077
C	24.299773830108	15.147508967188	18.886361860345
C	23.988914990622	15.527443712064	17.606469493730
H	23.816128693340	14.319515047009	19.407504114133
H	23.237878938908	15.059355228735	16.971761031020
P	25.787517866017	15.995401716915	21.245954707258

O	27.203697643660	16.484677772813	21.443345141490
C	24.591985524029	17.101446681517	22.067881007111
C	25.142818848159	18.091460057405	22.887151894859
C	23.206591110699	17.036530533162	21.886699465220
C	22.376432110880	17.932064955418	22.555947821829
C	24.309635514454	19.002010197690	23.534151943736
C	22.927576135419	18.917543198300	23.375118301901
H	22.770603041087	16.300113618296	21.206755468206
H	26.227824949527	18.140268303171	22.992186686652
H	21.294661800035	17.868951904783	22.428540769391
H	22.273376982409	19.626623014976	23.885814661779
H	24.741735046400	19.780529414126	24.165205793293
H	27.328754630076	11.399088424633	21.894207226358
C	25.270710965115	11.592875081956	22.521589901035
H	25.173955190067	10.541856635996	22.799802590193
H	23.245192922448	12.083587629460	23.083941054158
C	24.187334387667	12.455562089546	22.677374117284
C	26.477216917712	12.071821309317	22.011142361998
C	26.600101949145	13.411590470937	21.655024059477
H	23.460156000355	14.470346229648	22.472331442243
H	27.544740199292	13.797686052010	21.268502652279
C	24.306891206062	13.798739749950	22.323679458600
C	25.511467157699	14.279694806723	21.802755732070

EET between Tb $^5D_4$ and linker-centered T1
Electronic energy = -2861.329222320205 (hartree)
Geometry of the high level region. That of the low level region is the same as S0.

S	23.566998031067	16.935711740372	14.737081082678
C	24.199513538167	18.133812433194	13.650891081036
C	24.669766377998	17.429053534241	16.000759611276
C	25.173435959571	18.901436286690	14.258781631958
H	25.677838953769	19.725559645096	13.753594519461
C	25.441567144690	18.512253810421	15.583900011558
H	26.178002465153	18.997520921698	16.222407685649
P	23.640991343579	18.144345035827	11.928976621190
O	22.188020253377	17.775652001613	11.748850203606
C	24.734906450138	16.954955805924	11.083777940278
C	26.120080383626	16.888106550714	11.266361737303
C	24.093315181385	16.022817499675	10.263083073269
C	26.860436836353	15.914255600277	10.600884099134
C	24.835729370951	15.037664186857	9.615185122524
C	26.218712487100	14.987154471562	9.779416584962
H	26.623121116601	17.575395064529	11.951094546927
H	23.007956398093	16.074917551104	10.165362566987
H	27.941886307964	15.865232431292	10.736954096380
H	26.801923720670	14.214435454193	9.275047514168
H	24.331184485759	14.302167062960	8.986511432199
C	24.361738190416	22.505803981823	10.635119701710
C	22.941119192469	20.740624516492	11.465510515416
C	24.011032589414	19.842127535100	11.380035740204
C	25.253606782852	20.278414211901	10.912623242463
H	24.499205849386	23.548174332677	10.342590384339
C	25.427661122998	21.611586601237	10.544816332439
C	23.119361661255	22.070475910664	11.095797483836
H	26.085924452678	19.578651863482	10.817231637529
H	21.971120730193	20.379512011962	11.813270960107
H	22.283111047860	22.768741578561	11.161089129055
H	26.397874686731	21.950289532736	10.177232844004
S	25.778020871644	17.282650846992	18.536013590967
C	25.180893497777	16.056938407884	19.609476794166
C	24.707980971423	16.751234751410	17.258354036467
C	24.237415013548	15.258978154280	18.993024860197
C	23.965712276181	15.643882912917	17.666591009322
H	23.761964203927	14.412414240684	19.489478050832
H	23.258145096639	15.129267447502	17.016839266983
P	25.759916218974	16.065773283457	21.324366608177
O	27.197298174751	16.507765637503	21.472437333644
C	24.623193146158	17.199131063894	22.189887585769
C	25.222925089066	18.135463811082	23.036981116005
C	23.237715720059	17.218048803221	21.998223627254
C	22.455712961481	18.147186655834	22.679555664102
C	24.438449233227	19.074984543009	23.702909100978
C	23.055645376802	19.077364670657	23.528710050032
H	22.766937828141	16.524363025704	21.297010624440

H	26.308368032312	18.123364522552	23.144870234206
H	21.373903258806	18.155823389500	22.537597107656
H	22.439484246909	19.812248902529	24.050121058436
H	24.909622938526	19.810686956754	24.356907803109
H	27.222201771782	11.441518414087	21.953307567704
C	25.156877501108	11.663328674276	22.546101694519
H	25.037785777818	10.610712916971	22.809111660308
H	23.129315107658	12.179861029531	23.077772229583
C	24.084617989190	12.541867712846	22.693552842968
C	26.380467839670	12.127654888121	22.063687140339
C	26.532429657513	13.469347745818	21.725954357148
H	23.395346577571	14.572551311025	22.498304464892
H	27.488114840117	13.845904183771	21.357195444406
C	24.233087259010	13.887016205446	22.359376195667
C	25.455383641606	14.352339237861	21.866156797468

EET between Eu $^{5}D_{0}$ and linker-centered T1
Electronic energy = -2861.338192640550 (hartree)
Geometry of the high level region. That of the low level region is the same as S0.

S	23.539627981639	16.924533542148	14.743805723502
C	24.181604106450	18.126178728034	13.658959757503
C	24.653173445501	17.420277016094	16.017210196530
C	25.158356387446	18.905105206177	14.274608743182
H	25.656526039494	19.730242854752	13.764565413642
C	25.428320318550	18.527977353029	15.586898766391
H	26.160680019661	19.017353108090	16.226705929467
P	23.644183133634	18.126677654272	11.931971205715
O	22.201357300111	17.725973476670	11.738470040422
C	24.773952787099	16.963525938298	11.097175381869
C	26.160858608945	16.945826825571	11.278953468347
C	24.163825073822	16.001055850618	10.287490494667
C	26.934134144235	15.992543793348	10.621631088672
C	24.939607710699	15.034617961419	9.650292749479
C	26.323837397217	15.034434591516	9.811917045748
H	26.640903983203	17.656327427188	11.956541385779
H	23.077208772262	16.015090077442	10.188947975638
H	28.016961095827	15.984059159337	10.755641272703
H	26.932930180013	14.277206708014	9.314764673492
H	24.460351671537	14.274601272654	9.031025458342
C	24.287864378901	22.509845761663	10.666030879559
C	22.903020773553	20.717387162027	11.498886007938
C	23.984136466976	19.834277370575	11.391912919711
C	25.213705561739	20.292433479812	10.911293230581
H	24.407925568796	23.556810277063	10.382200345347
C	25.364363442211	21.631153315861	10.553244461992
C	23.057793802646	22.052787373652	11.138933886186
H	26.054375063126	19.605837348651	10.797219407567
H	21.942296715049	20.340571084006	11.855589145416
H	22.212615515401	22.738399421054	11.221447658756
H	26.324697170983	21.986251659786	10.175350825566
S	25.803841795582	17.280239610827	18.525965611490
C	25.188856199109	16.059904707160	19.603532819427
C	24.711088719078	16.755591353435	17.244602259070
C	24.231834741177	15.259384878003	18.985162763017
C	23.956038667365	15.632881237311	17.672114562857
H	23.756068294256	14.417726965103	19.489894085745
H	23.244651045098	15.120391097283	17.025269216849
P	25.759651725350	16.071274589434	21.319390998414
O	27.197980676824	16.509313631452	21.470616347598
C	24.625191761058	17.209390351451	22.181940405742
C	25.227714009095	18.150115105526	23.022183811276
C	23.239032709218	17.227077783357	21.994946353656
C	22.459161583746	18.159015845085	22.674938130256
C	24.445322344460	19.092461460076	23.686508429534
C	23.061866528300	19.093285680670	23.517588702513
H	22.765402014882	16.529897852663	21.299088436673
H	26.313507427707	18.138417167811	23.126556394390
H	21.376797006253	18.166364061190	22.537243279305
H	22.447265208327	19.830048907019	24.038177707604
H	24.918630507021	19.831330201163	24.335366415655
H	27.209287706949	11.443562424410	21.949609075576
C	25.145454046643	11.671037207170	22.545423088403
H	25.024070080052	10.618911947313	22.809318294031
H	23.120223869857	12.193290185988	23.080281659995

C	24.075793050019	12.552499181324	22.694094813084
C	26.369466947642	12.131913255731	22.060765805220
C	26.524333615326	13.472970324052	21.721809802181
H	23.391443810298	14.584665776156	22.499080449874
H	27.480577475335	13.846779032963	21.351655798193
C	24.227207440333	13.897049934662	22.358730013355
C	25.449790616073	14.358990589182	21.862977676191
